# Retina as a potential biomarker in schizophrenia spectrum disorders: a systematic review and meta-analysis of optical coherence tomography and electroretinography

**DOI:** 10.1038/s41380-023-02340-4

**Published:** 2023-12-11

**Authors:** Hiroshi Komatsu, Goh Onoguchi, Steven M. Silverstein, Stefan Jerotic, Atsushi Sakuma, Nobuhisa Kanahara, Yoshihisa Kakuto, Takashi Ono, Takeshi Yabana, Toru Nakazawa, Hiroaki Tomita

**Affiliations:** 1 Department of Psychiatry, Tohoku University Hospital, Sendai, Japan; 2 Miyagi Psychiatric Center, Natori, Japan; 3 Department of Psychiatry, Tohoku University Graduate School of Medicine, Sendai, Japan; 4 Department of Psychiatry, University of Rochester Medical Center, Rochester, NY USA; 5 Clinic for Psychiatry, University Clinical Centre of Serbia, Belgrade, Serbia; 6 Faculty of Medicine, University of Belgrade, Belgrade, Serbia; 7 Department of Psychiatry, Chiba University Graduate School of Medicine, Chiba, Japan; 8 Division of Medical Treatment and Rehabilitation, Chiba University Center for Forensic Mental Health, Chiba, Japan; 9 Department of Community Psychiatry, Tohoku University Graduate School of Medicine, Sendai, Japan; 10 Department of Ophthalmology, Tohoku University Graduate School of Medicine, Sendai, Japan; 11 Department of Ophthalmic Imaging and Information Analytics, Tohoku University Graduate School of Medicine, Sendai, Japan; 12 Department of Retinal Disease Control, Tohoku University Graduate School of Medicine, Sendai, Japan; 13 Department of Advanced Ophthalmic Medicine, Tohoku University Graduate School of Medicine, Sendai, Japan

**Keywords:** Schizophrenia, Prognostic markers

## Abstract

**Introduction:**

Abnormal findings on optical coherence tomography (OCT) and electroretinography (ERG) have been reported in participants with schizophrenia spectrum disorders (SSDs). This study aims to reveal the pooled standard mean difference (SMD) in retinal parameters on OCT and ERG among participants with SSDs and healthy controls and their association with demographic characteristics, clinical symptoms, smoking, diabetes mellitus, and hypertension.

**Methods:**

Using PubMed, Scopus, Web of Science, and PSYNDEX, we searched the literature from inception to March 31, 2023, using specific search terms. This study was registered with PROSPERO (CRD4202235795) and conducted according to PRISMA 2020.

**Results:**

We included 65 studies in the systematic review and 44 in the meta-analysis. Participants with SSDs showed thinning of the peripapillary retinal nerve fiber layer (pRNFL), macular ganglion cell layer- inner plexiform cell layer, and retinal thickness in all other segments of the macula. A meta-analysis of studies that excluded SSD participants with diabetes and hypertension showed no change in results, except for pRNFL inferior and nasal thickness. Furthermore, a significant difference was found in the pooled SMD of pRNFL temporal thickness between the left and right eyes. Meta-regression analysis revealed an association between retinal thinning and duration of illness, positive and negative symptoms. In OCT angiography, no differences were found in the foveal avascular zone and superficial layer foveal vessel density between SSD participants and controls. In flash ERG, the meta-analysis showed reduced amplitude of both a- and b-waves under photopic and scotopic conditions in SSD participants. Furthermore, the latency of photopic a-wave was significantly shorter in SSD participants in comparison with HCs.

**Discussion:**

Considering the prior report of retinal thinning in unaffected first-degree relatives and the results of the meta-analysis, the findings suggest that retinal changes in SSDs have both trait and state aspects. Future longitudinal multimodal retinal imaging studies are needed to clarify the pathophysiological mechanisms of these changes and to clarify their utility in individual patient monitoring efforts.

## Introduction

Schizophrenia is in most cases a chronic mental illness with varying degrees of positive and negative symptoms, cognitive dysfunction, and decline in real-world functioning. No clinically applicable state/trait biomarkers for use in monitoring and prediction efforts for participants with schizophrenia have been identified, and further research in this field is warranted. Prior brain imaging studies suggest that schizophrenia participants show volume loss in gray and white matter and abnormalities in the microstructure of white matter [1]. However, brain imaging is an expensive technique that is not yet feasible to incorporate into everyday clinical practice. In contrast, the retina is the part of the central nervous system that can be directly observed noninvasively and with high accuracy, using a retinal imaging technique known as optical coherence tomography (OCT). The retina consists of a layered structure composed of neurons such as ganglion cells, bipolar cells, photoreceptor cells, horizontal cells, and amacrine cells, as well as glial cells such as Müller cells [1]. Previous brain imaging studies have shown that reduced thickness of the retinal layers is associated with decreased brain volume and abnormal white matter integrity in population-based cohort studies [2, 3].

Recently, there has been an increasing number of studies examining the thickness of the retinal layer using OCT in schizophrenia participants. Prior meta-analytic (MA) studies have supported the hypothesis of thinning of retinal neural layers and structures such as the pRNFL, macula region, and ganglion cell layer-inner plexiform layer (GCL-IPL) measured at the macula, in addition to enlargement of the optic disc (presumed to be due to neurodegeneration of surrounding neural tissue) [4–9]. The latest MA includes studies through January 31, 2023 [8]. The results of the MA of pRNFL average thickness and pRNFL thickness in the four quadrants are generally highly heterogeneous. Several studies have indicated correlations between retinal thickness in both macular and peripapillary regions and duration of disease, as well as positive and negative symptoms [10–16]. However, as with brain imaging, there are potential confounds from factors associated with schizophrenia that can affect neural and vascular health, such as smoking, diabetes, and hypertension [17–20]. Silverstein et al. previously reported that after adjusting for diabetes and hypertension, the difference in retinal thickness among participants with schizophrenia and healthy controls (HCs) was no longer significant (although some macula findings were at the trend level) [21]. However, studies that have excluded participants with diabetes or hypertension have generally reported evidence of retinal neurodegeneration in SSDs [9].

In addition to relatively consistent evidence of retinal neural layer thinning in SSDs, recent evidence indicates pathology of the retinal microvasculature as well, using a recently developed extension of OCT called OCT angiography (OCTA) [22]. OCTA allows noninvasive measurement of retinal perfusion density, in addition to characteristics of retinal capillaries (e.g., width, total vessel length, extent of branching, tortuosity, and fractal dimension). Several studies have used OCTA to investigate these characteristics in participants with schizophrenia. Abnormalities in the density of retinal blood vessels, in vessel width, and fractal dimension [23], in addition to enlargement of the foveal avascular zone (FAZ) (due to loss of blood vessels at the fovea) and change in vascular tortuosity and branching, have been reported in participants with schizophrenia [14, 24–27]. While the findings are not consistent across all studies, this may be due to differences in participants (younger, more acutely ill vs. older, more chronically ill participants) and differences in which retinal vascular layers were imaged (e.g., superficial versus deep) across studies. Overall, however, OCTA findings in SSDs parallel brain imaging and postmortem brain studies that indicated microvascular abnormalities in participants with schizophrenia [28–31]. As with OCT findings, some of the OCTA findings were primarily attributable to the higher prevalence of diabetes and hypertension in this population, rather than to schizophrenia itself, illustrating the need to consider these confounding variables when exploring the potential role of retinal features as biomarkers in SSDs. Importantly, though, even after controlling for medical illness or excluding SSD participants with those conditions, independent effects of SSDs can be observed on OCTA (e.g., reduced FD in both eyes) [25].

Finally, several studies have indicated asymmetry of retinal thickness in normal individuals [32, 33], and some of the retinal findings in SSD participants have been stronger in one eye [15, 34]. This parallels findings of asymmetry in brain structure in participants with schizophrenia [35, 36]. Considering the association between retinal thickness and brain structure in participants with psychotic disorders [37], asymmetry in retinal thickness in schizophrenia participants is also assumed to influence heterogeneity in the estimates in each study in the MA. This finding, and the literature reviewed above, emphasizes the complexity of retinal changes in schizophrenia and sets the stage for our systematic review (SR) and MA, aiming to disentangle the effects of schizophrenia from underlying health conditions on retinal integrity.

In SSDs, changes in retinal functioning can be observed in addition to those in retinal structure. Retinal function in SSDs has most often been measured using electroretinography (ERG), particularly the flash ERG (fERG). The most commonly studied waveforms are the negative a-wave, reflecting the hyper-polarization of photoreceptors. In photopic conditions, a-wave mainly reflects the function of cone cells. On the other hand, in scotopic conditions, the a-wave reflects the function of rod cells. The positive b-wave that follows the a-wave, reflects the function of bipolar cells and Müller cells. The photopic negative response (PhNR) is a negative wave following the b-wave and is thought to originate mainly from retinal ganglion cells. Several prior fERG studies have shown changes in amplitudes and latencies of a-wave, b-wave, and PhNR in schizophrenia participants [38–40].

The first aim of this study was to provide an updated MA based on more recent studies of retinal thickness in participants with SSDs and to determine the association between pooled estimates of the retinal thicknesses and the following characteristics: demographic data, symptom severity, diabetes, hypertension, and smoking. We also sought to clarify the degree of asymmetry in OCT findings in SSDs. The second aim was to conduct an MA on OCTA findings in SSD participants relative to HCs. Finally, we conducted the MA and meta-regression to investigate the difference in amplitude and latency of a- and b-wave in fERG under photopic and scotopic conditions in addition to PhNR amplitude among SSD participants and HCs, and the association of clinical factors with their overall estimates.

## Methods

### Search strategy

We performed an SR and MA according to the Preferred Reporting Items for Systematic reviews and Meta-Analyses PRISMA2020 guidelines [41]. The protocol was registered in the International Prospective Register of Systematic Reviews (PROSPERO) database (CRD4202235795). Two investigators (HK and GO) independently searched using PubMed, Scopus, Web of Science, and PSYNDEX for retina-related reports in participants with SSDs from the database inception to March 31, 2023. We used the following terms to search the reports: (macula* OR retina* OR “optical coherence” OR electroretinograph*) and (schizophreni* OR psychosis OR “treatment resistant schizophrenia” OR “treatment resistant psychosis” OR “treatment refractory schizophrenia” OR “treatment refractory psychosis” OR clozapine). All reports identified in the search were imported into EndNote (version X9.3.3, Clarivate Analytics, Philadelphia, PA, USA) as RIS-formatted files. Two investigators (HK and GO) independently screened and assessed the eligibility of the reports identified in the search using EndNote.

### Selection criteria

Inclusion criteria were: 1) cross-sectional or prospective studies using OCT, OCTA, or fERG to measure retinal parameters in both HCs and participants with SSDs (ICD-10 code F20–29 and DSM criteria-based schizophrenia, schizoaffective disorder, brief psychotic disorder, and delusional disorder); 2) studies with a score on the Newcastle–Ottawa Scale (NOS) of ≥ 6 points; 3) studies included means and standard deviations of retinal parameters, and reported the number of participants in both SSD and HC groups. Exclusion criteria were 1) inclusion of cases overlapping with those in other papers; 2) lack of necessary data on the retinal parameters; 3) combining of schizophrenia and bipolar disorder participants in case groups; 4) non-inclusion of HCs group; and 5) a study containing only choroidal data. For articles in which data required for MA were not included, we contacted the corresponding authors by e-mail and requested the data needed for MA.

### Assessments of quality of studies and certainty in the body of evidence

We used the NOS to assess the quality of each study included in the SR and MA [42]. NOS is a nine-point scale, with four points for selection, two points for comparability, and three points for exposure; a higher NOS score indicates a higher-quality study. Using the NOS, two researchers (HK and GO) independently assessed the quality of each study. When the NOS scores differed among the two researchers, the final NOS score was determined by discussion. We used the GRADE profiler v3.6 to assess the certainty in the body of evidence [43].

### Statistical analysis

The supplementary information provides a detailed description of the statistical analysis.

We performed an MA to investigate the difference in OCT retinal parameters [(pRNFL average thickness, pRNFL thickness in four quadrants, macular average thickness (MAT), macular volume (MV), macular thickness (MT) in Early Treatment Diabetic Retinopathy Study (ETDRS) grid [44], macular GCL-IPL thickness (mGCL-IPL), optic cup volume (OCV), cup-to-disk area ratio (CDR), FAZ, and superficial foveal vessel density (VD)] among SSD participants and HCs. We also evaluated the differences in amplitude and latency of fERG a- and b-waves under scotopic and photopic conditions and PhNR amplitude among the two groups in an MA.

We adopted a random-effects model to calculate the pooled standardized mean difference (SMD) [45]. Influence analysis was performed using leave-one-out (LOO) analysis [46], and we created a graphical display of study heterogeneity (GOSH) analysis of outliers influencing statistical heterogeneity [47]. To assess publication bias, contour-enhanced funnel plots were plotted, and if the number of studies included in the MA was ≥10, we also performed Egger’s regression analysis [48]. If publication bias was suspected, we recalculated the pooled SMD after adjusting for publication bias using the trim-and-fill method [49] after removing outliers.

We performed a meta-regression analysis to evaluate the effects of SSD participant age, duration of illness, percentage of male SSD participants, OCT device type (time domain [TD]–OCT, spectral domain (SD)–OCT, or swept source [SS]–OCT), psychiatric symptoms, antipsychotic dosage (chlorpromazine equivalent [mg/day]), NOS, smoking (%), and body mass index (kg/m^2^) on the pooled estimates of the retinal parameters.

In the subgroup analysis, we compared the pooled estimates of retinal thickness between the left and right eyes, and performed a MA for only studies that excluded diabetes and hypertension (exclusion group) to assess the effect of the diabetes and hypertension on the retinal parameters.

We used “meta” [50], “metafor” [51], and “dmetar” [52] packages in R version 4.2.0 for MA. Statistical significance was set at *P* < 0.05 for all analyses.

## Results

**Fig. 1:**
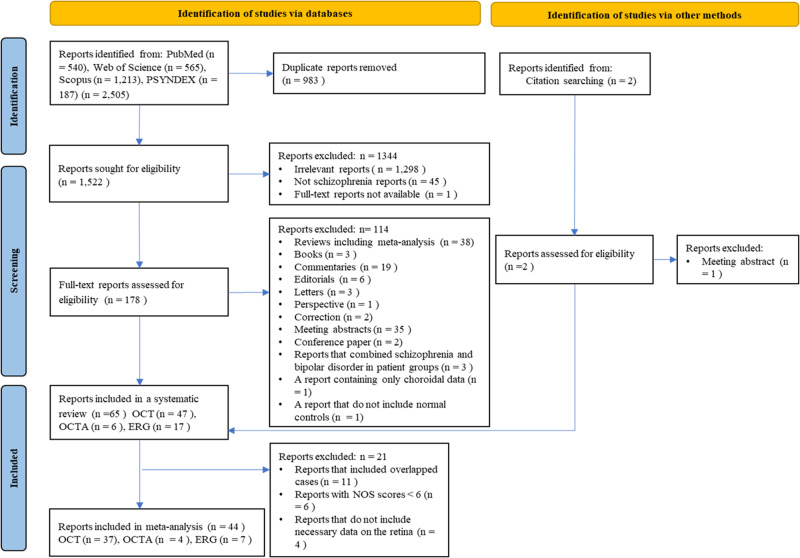
Flow diagram of the systematic review and meta-analysis according to PRISMA 2020. PRISMA preferred reporting items for systematic reviews and meta-analyses, OCT optical coherence tomography, OCTA optical coherence tomography angiography, ERG electroretinography, NOS Newcastle-Ottawa Scale.

After searching the four databases, we identified a total of 2505 reports. After removing duplicates and excluding irrelevant reports (i.e., reports that did not include schizophrenia) and reports for which full text was unavailable, we screened the abstracts of 1522 reports. We assessed the full text of the remaining 178 reports. We included 64 reports in the SR [10–16, 21, 23–25, 34, 38–40, 53–101], after excluding 38 reviews [4–9, 102–133] including meta-analyses [4–9], three books [134–136], 19 commentaries [137–155], six editorials [156–161], three letters [162–164], one perspective [22], two corrections [165, 166], 35 meeting abstracts [167–201], two conference papers [202, 203], three reports including both schizophrenia and bipolar disorder in participant groups [37, 204, 205], one report that included only choroidal data [206], and one report that did not include HCs [207]. In addition, after carefully reading the citations, we added to the SR one report on retinas in participants with schizophrenia not identified in the database search [208]. From a total of 65 reports included in the SR (Fig. 1, Tables S1–3), 44 reports were included in the MA, excluding 11 reports with overlapping cases [38, 71, 72, 76, 80, 89–91, 93, 95, 97], six reports with NOS score < 6 [66, 73, 75, 99–101], and four reports without the required numerical retinal data [10, 24, 59, 81] (Fig. 1). Tables S1–3 describe the NOS score of all reports included in the SR. Since 10 of the 64 reports in the SR had NOS scores that differed between the two researchers, the final NOS score was determined through discussion.

### OCT Findings

Table S1 shows the main findings of the OCT studies included in the SR. Thirty-seven studies with a total of 368,420 eyes of 202,982 participants (2680 participants with SSDs and 200,302 HCs) were included in the MA (Fig. 1).

#### pRNFL thickness

**Fig. 2:**
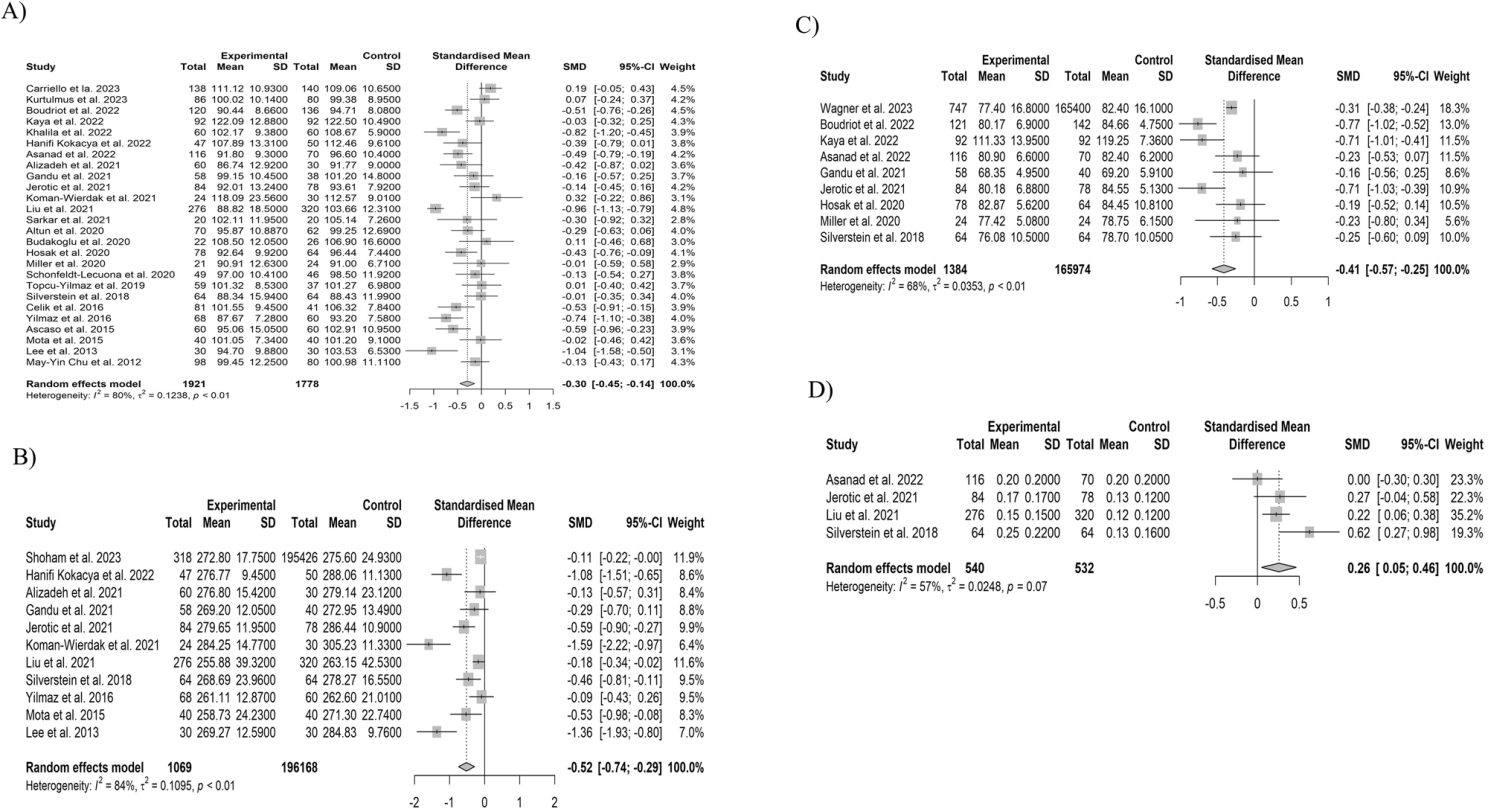
Results of the meta-analysis of pRNFL average thickness, macular average thickness, mGCL-IPL, and optic cup volume. **A** pRNFL average. **B** Macular average thickness. **C** Macular GCL-IPL. **D** Optic cup volume. Horizontal bars indicate 95% confidence intervals (95% CIs). Total indicates the total number of participants’ eyes for which the mean and standard deviation were calculated. SMD standardized mean difference, SD standard deviation, pRNFL peripapillary retinal nerve fiber layer, mGCL-IPL macular ganglion cell layer-inner plexiform layer.

**Fig. 3:**
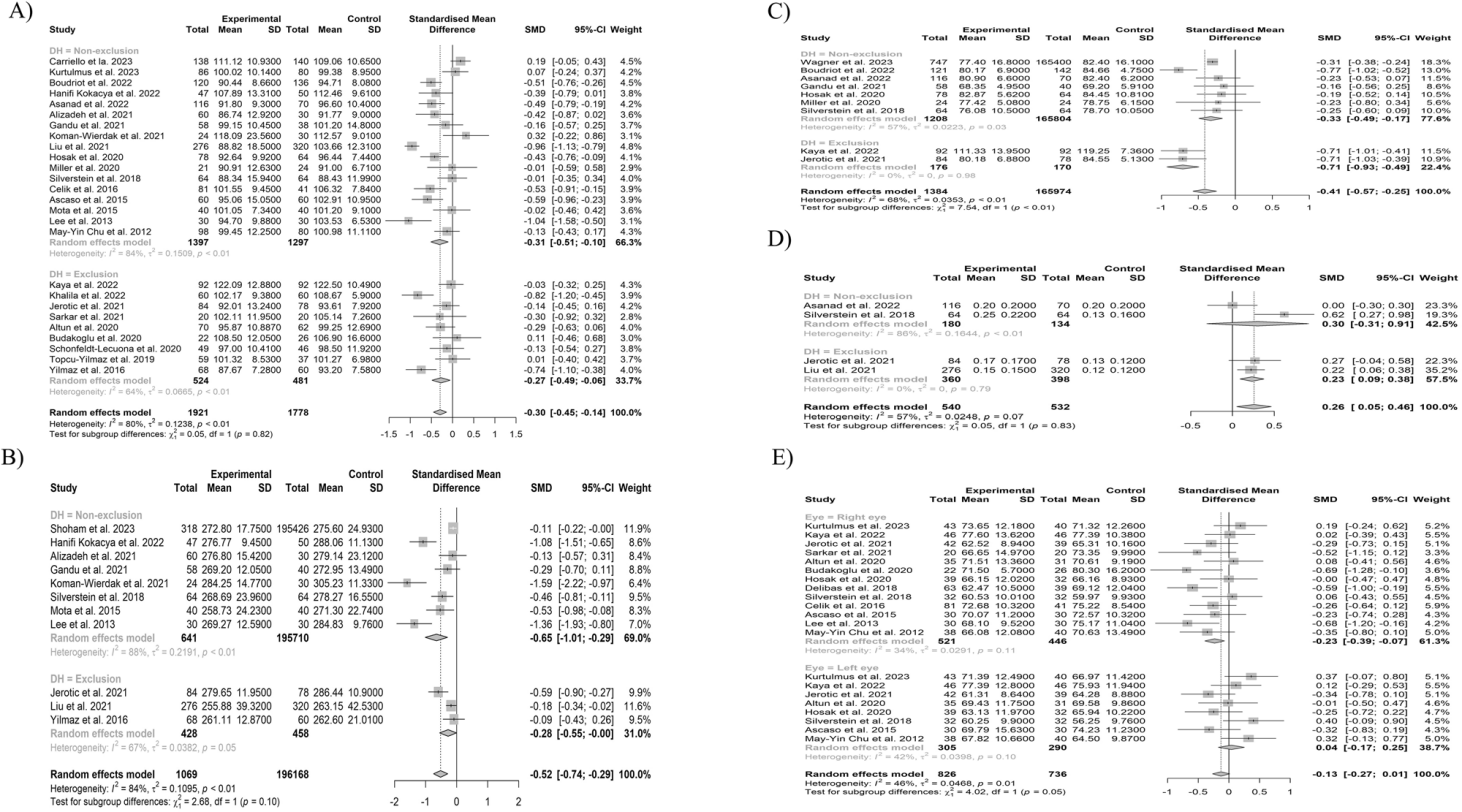
Results of the subgroup analysis. **A** The results of the subgroup analysis between the studies in which diabetes mellitus and hypertension were excluded (exclusion) and not excluded (non-exclusion) in pRNFL average thickness. **B** The results of the subgroup analysis between exclusion and non-exclusion in macular average thickness. **C** The results of the subgroup analysis between exclusion and non-exclusion in mGCL-IPL. **D** The results of the subgroup analysis between exclusion and non-exclusion in optic cup volume. **E** The results of the subgroup analysis between right and left eyes in pRNFL temporal thickness. Horizontal bars indicate 95% confidence intervals (95% CIs). Total indicates the total number of participants’ eyes for which the mean and standard deviation were calculated. SMD standardized mean difference, SD standard deviation, pRNFL peripapillary retinal nerve fiber layer, mGCL-IPL macular ganglion cell layer-inner plexiform layer.

In the MA of pRNFL average thickness and superior, inferior, temporal, and nasal thickness, we included 26 studies (1921 eyes in 1083 SSD participants and 1778 eyes in 995 HCs), 17 studies (1389 eyes in 755 SSD participants and 1343 eyes in 733 HCs), 17 studies (1389 eyes of 755 SSD participants and 1343 eyes of 855 HCs), 21 studies (1449 eyes of 855 SSD participants and 1283 eyes of 742 HCs), and 22 studies (1496 eyes of 902 SSD participants and 1333 eyes of 792 HCs), respectively. The pRNFL average thickness and pRNFL thickness in four quadrants were significantly thinner in SSD participants (Figs. 2, S1). The LOO analysis showed that the difference remained significant for pRNFL thicknesses, except for pRNFL nasal thickness (Figs. S2–6). GOSH analysis identified one outlier [65] in pRNFL average thickness (Fig. S7). After removing the outlier, statistical significance remained for pRNFL average thickness (Table S4). For pRNFL superior, inferior, temporal and nasal thickness, we identified four [13, 21, 63, 65], three [12, 55, 65], six [11, 21, 34, 53, 82, 83], and two outliers [13, 85], respectively (Figs. S8–11). After removing the outliers, the significant difference was lost for only pRNFL nasal thickness (Table S4). For pRNFL average thickness and pRNFL thickness in four quadrants, a counter-enhanced funnel plot and Egger’s regression test showed no significant publication bias (Figs. S12, S13 and Table S5). The meta-regression analysis showed no association between any of the explanatory variables and the overall effect size for pRNFL average thickness (Table 1). On the other hand, we observed a negative correlation between the pooled estimates of pRNFL superior thickness, duration of illness, and Positive and Negative Symptom Scale (PANSS) negative scale score (Table 1). The pooled estimate of pRNFL inferior thickness was negatively associated with SSD participant age and duration of illness (Table 1). Results of the subgroup analysis showed that the differences remained significant except for pRNFL inferior and nasal thickness, even in the exclusion group (Figs. 3,S14). We found a significant difference between the right and left eyes in the pooled estimates of pRNFL temporal thickness (Figs. 3, S15). Due to high heterogeneity for all pRNFL thicknesses, the GRADE rating result was “very low” (Table S6).

**Table 1:** Meta-regression analysis of the association between the explanatory variables and pooled estimates of pRNFL thicknesses.

Retinal parameter	No. of studies	Explanatory variable	Coefficient	95% CI	SE	*p* value
pRNFL average thickness	26	SSD participant age	−0.0144	[−0.0412; 0.0125]	0.0137	0.2941
	26	Sex (% of male SSD participants)	−0.0058	[−0.0183; 0.0068]	0.0064	0.368
	19	Duration of illness (M)	−0.0012	[−0.0034; 0.0010]	0.0011	0.274
	12	PANSS total score	−0.0111	[−0.0253; 0.0032]	0.0073	0.1271
	7	PANSS positive scale score	−0.0356	[−0.0927; 0.0215]	0.0291	0.2215
	7	PANSS negative scale score	−0.0284	[−0.0936; 0.0367]	0.0332	0.3925
	6	PANSS general psychopathology scale score	−0.0273	[−0.0728; 0.0182]	0.0232	0.2397
	9	Antipsychotic dose (chlorpromazine equivalent [mg/day])	0.0002	[−0.0008; 0.0013]	0.0005	0.657
	8	Smoking (%)	0.0016	[−0.0165; 0.0197]	0.0092	0.8637
	8	Body mass index (kg/m^2^)	0.0607	[−0.1077; 0.2291]	0.0859	0.4798
	26	OCT device type	0.0817	[−0.3799; 0.5433]	0.2355	0.7287
	2	TD-OCT				
	23	SD-OCT				
	1	SS-OCT				
	26	NOS	−0.1093	[−0.2701; 0.0515]	0.0821	0.1828
pRNFL thickness in four quadrants						
Superior thickness	17	SSD participant age	−0.0122	[−0.0397; 0.0152]	0.014	0.3822
	17	Sex (% of male SSD participants)	−0.0124	[−0.0250; 0.0002]	0.0064	0.0533
	12	Duration of illness (M)	−0.0024	[−0.0048; −0.0001]	0.0012	0.0413
	7	PANSS total score	−0.0081	[−0.0168; 0.0006]	0.0044	0.0672
	6	PANSS positive scale score	−0.0249	[−0.0604; 0.0107]	0.0182	0.1708
	6	PANSS negative scale score	−0.0325	[−0.0631; −0.0019]	0.0156	0.0373
	6	PANSS general psychopathology scale score	−0.0265	[−0.0531; 0.0001]	0.0136	0.0508
	5	Antipsychotic dose (chlorpromazine equivalent [mg/day])	−0.0008	[−0.0022; 0.0007]	0.0007	0.2873
	4	Smoking (%)	−			
	4	Body mass index (kg/m^2^)	−			
	17	OCT device type	0.0027	[−0.4774; 0.4829]	0.245	0.9911
	2	TD-OCT				
	15	SD-OCT				
	0	SS-OCT				
	17	NOS	−0.115	[−0.2687; 0.0387]	0.0784	0.1427
Inferior thickness	17	SSD participant age	−0.0261	[−0.0487; −0.0034]	0.0115	0.0239
	17	Sex (% of male SSD participants)	−0.0003	[−0.0125; 0.0120]	0.0063	0.966
	12	Duration of illness (M)	−0.0029	[−0.0043; −0.0016]	0.0007	<0.0001
	7	PANSS total score	−0.0022	[−0.0142; 0.0098]	0.0061	0.7142
	6	PANSS positive scale score	−0.0021	[−0.0486; 0.0443]	0.0237	0.928
	6	PANSS negative scale score	−0.0047	[−0.0495; 0.0400]	0.0228	0.8364
	6	PANSS general psychopathology scale score	−0.0045	[−0.0426; 0.0336]	0.0194	0.8176
	5	Antipsychotic dose (chlorpromazine equivalent [mg/day])	−0.0006	[−0.0015; 0.0002]	0.0004	0.1404
	4	Smoking (%)	−			
	4	Body mass index (kg/m^2^)	−			
	17	OCT device type	−0.125	[−0.5774; 0.3274]	0.2308	0.5881
	2	TD-OCT				
	15	SD-OCT				
		SS-OCT				
	17	NOS	−0.111	[−0.2574; 0.0354]	0.0747	0.1371
Temporal thickness	21	SSD participant age	0.0007	[−0.0236; 0.0250]	0.0124	0.9574
	21	Sex (% of male SSD participants)	−0.0059	[−0.0157; 0.0039]	0.005	0.2359
	15	Duration of illness (M)	0.001	[−0.0012; 0.0033]	0.0011	0.3781
	9	PANSS total score	−0.0017	[−0.0138; 0.0104]	0.0062	0.7811
	5	PANSS positive scale score	−0.0135	[−0.0566; 0.0297]	0.022	0.5411
	5	PANSS negative scale score	0.0022	[−0.0418; 0.0462]	0.0225	0.9212
	5	PANSS general psychopathology scale score	0.0017	[−0.0372; 0.0407]	0.0199	0.931
	7	Antipsychotic dose (chlorpromazine equivalent [mg/day])	0.0000	[−0.0012; 0.0012]	0.0006	0.9473
	5	Smoking (%)	0.0002	[−0.0116; 0.0120]	0.006	0.9698
	4	Body mass index (kg/m^2^)	−			
	21	OCT device type	−0.0484	[−0.4543; 0.3574]	0.2071	0.8151
	2	TD-OCT				
	19	SD-OCT				
	0	SS-OCT				
	21	NOS	−0.0869	[−0.2153; 0.0415]	0.0655	0.1845
Nasal thickness	22	SSD participant age	0.0018	[−0.0273; 0.0309]	0.0148	0.9041
	22	Sex (% of male SSD participant)	−0.0092	[−0.0208; 0.0023]	0.0059	0.1162
	15	Duration of illness (M)	−0.0003	[−0.0034; 0.0028]	0.0016	0.8609
	10	PANSS total score	−0.0122	[−0.0244; 0.0001]	0.0063	0.052
	5	PANSS positive scale score	−0.0408	[−0.1007; 0.0191]	0.0306	0.1816
	5	PANSS negative scale score	−0.0492	[−0.1163; 0.0179]	0.0342	0.1506
	5	PANSS general psychopathology scale score	−0.0433	[−0.1029; 0.0164]	0.0304	0.1551
	7	Antipsychotic dose (chlorpromazine equivalent [mg/day])	−0.0006	[−0.0013; 0.0002]	0.0004	0.148
	5	Smoking (%)	0.0043	[−0.0064; 0.0149]	0.0054	0.4316
	4	Body mass index (kg/m^2^)	−			
	22	OCT device type	0.1172	[−0.3749; 0.6093]	0.2511	0.6407
	2	TD-OCT				
	20	SD-OCT				
	0	SS-OCT				
	22	NOS	−0.0299	[−0.1962; 0.1365]	0.0849	0.725

*CI* confidence interval, *NOS* Newcastle–Ottawa Scale, *PANSS* Positive and Negative Symptom Scale, *pRNFL* peripapillary retinal nerve fiber layer, *SD-OCT* spectral domain–optical coherence tomography, *SS-OCT* swept source–optical coherence tomography, *TD-OCT* time domain–optical coherence tomography.

#### MAT and MV

For MA of MAT and MV, we included 11 studies (1069 eyes of 573 SSD participants and 196,168 eyes of 98,124 HCs) and 14 studies (1312 eyes of 660 SSD participants and 103,998 eyes of 52,014 HCs), respectively, in the MA. Participants with SSDs showed a significant thinning of MAT and a significant reduction in MV (Figs. 2,S16). The LOO analysis showed that a significant difference remained after removing each study for MAT and MV (Figs. S17, S18). In the GOSH analysis of MAT and MV, we identified [54] and three studies [11, 34, 87], respectively, as outliers (Figs. S19, S20). The significant difference remained after removing outliers for MAT and MV (Table S7). Counter-enhanced funnel plot and Egger’s regression test showed significant publication bias in MAT (Fig. S21, Table S5). The significant difference was lost after adjusting for publication bias in MAT (Table S5). No significant publication bias appeared to exist in MV (Fig. S21, Table S5). SSD participant age and duration of illness were positively associated with the overall estimates of MAT (Table 2). There was no association between any of the explanatory variables and the pooled estimate of MV (Table 2). The subgroup analysis showed that differences remained significant, even in the exclusion group, for both MAT and MV (Figs. 3,S22). We found no significant difference between the right and left eyes in the pooled estimates of MAT and MV (Fig. S23). Due to the high heterogeneity and publication bias for MAT and the high heterogeneity for MV, the GRADE rating results were “very low” (Table S6).

**Table 2:** Meta-regression analysis of the association between the explanatory variables and pooled estimates of macular thicknesses.

Retinal parameter	No. of studies	Explanatory variable	Coefficient	95% CI	SE	*p* value
Macular average thickness	11	SSD participant age	0.0409	[0.0152; 0.0667]	0.0131	0.0018
	11	Sex (% of male SSD participants)	0.0036	[−0.0119; 0.0190]	0.0079	0.6496
	5	Duration of illness (M)	0.0046	[0.0005; 0.0086]	0.0021	0.0289
	3	PANSS total score	—			
	2	PANSS positive scale score	—			
	2	PANSS negative scale score	—			
	2	PANSS general psychopathology scale score	—			
	3	Antipsychotic dose (chlorpromazine equivalent [mg/day])	—			
	2	Smoking (%)	—			
	2	Body mass index (kg/m^2^)	—			
	11	OCT device type	0.2468	[−0.5782; 1.0719]	0.421	0.5576
	0	TD-OCT				
	10	SD-OCT				
	1	SS-OCT				
	11	NOS	−0.0341	[−0.0052; 0.0002]	0.1632	0.0673
Macular volume	14	SSD participant age	−0.0154	[−0.0362; 0.0054]	0.0106	0.1469
	14	Sex (% of male SSD participants)	0.0024	[−0.0106; 0.0155]	0.0067	0.7136
	9	Duration of illness (M)	−0.0025	[−1.8300; 0.0673]	0.0014	0.0673
	4	PANSS total score	—			
	3	PANSS positive scale score	—			
	3	PANSS negative scale score	—			
	2	PANSS general psychopathology scale score	—			
	6	Antipsychotic dose (chlorpromazine equivalent [mg/day])	−0.0002	[−0.0010; 0.0005]	0.0004	0.5449
	3	Smoking (%)	—			
	5	Body mass index (kg/m^2^)	0.0753	[−0.1390; 0.2896]	0.1094	0.4911
	14	OCT device type	−0.2168	[−0.5128; 0.0792]	0.1510	0.1512
	2	TD-OCT				
	11	SD-OCT				
	1	SS-OCT				
	14	NOS	−0.1585	[−0.3336; 0.0166]	0.0893	0.0761
Macular thickness in ETDRS grid						
Macular central foveal thickness	20	SSD participant age	0.034	[0.0011; 0.0669]	0.0168	0.0426
	20	Sex (% of male SSD participants)	−0.0021	[−0.0152; 0.0109]	0.0066	0.7472
	13	Duration of illness (M)	0.0038	[0.0007; 0.0070]	0.0016	0.0175
	11	PANSS total score	−0.0043	[−0.0213; 0.0126]	0.0087	0.6156
	6	PANSS positive scale score	−0.1911	[−0.2955; −0.0868]	0.0532	0.0003
	6	PANSS negative scale score	0.0859	[−0.0453; 0.2171]	0.067	0.1996
	5	PANSS general psychopathology scale score	0.0257	[−0.1103; 0.1617]	0.0694	0.7112
	10	Antipsychotic dose (chlorpromazine equivalent [mg/day])	0.0002	[−0.0007; 0.0011]	0.0004	0.6636
	7	Smoking (%)	0.0038	[−0.0082; 0.0159]	0.0061	0.5356
	5	Body mass index (kg/m^2^)	−0.0426	[−0.1677; 0.0825]	0.0638	0.5047
	20	OCT device type	0.2285	[−0.2974; 0.7543]	0.2683	0.3945
	18	TD-OCT				
	1	SD-OCT				
	1	SS-OCT				
	20	NOS	0.0002	[−0.1816; 0.1820]	0.0927	0.9982
Inner ring						
Superior thickness	11	SSD participant age	0.0113	[−0.0288; 0.0515]	0.0205	0.5801
	11	Sex (% of male SSD participants)	0.0055	[−0.0064; 0.0174]	0.0061	0.3624
	6	Duration of illness (M)	−0.0007	[−0.0026; 0.0012]	0.001	0.4577
	5	PANSS total score	−0.0038	[−0.0120; 0.0044]	0.0042	0.3644
	3	PANSS positive scale score	—			
	3	PANSS negative scale score	—			
	3	PANSS General psychopathology scale score	—			
	2	Antipsychotic dose (chlorpromazine equivalent [mg/day])	—			
	4	Smoking (%)	—			
	2	Body mass index (kg/m^2^)	—			
	11	OCT device type	—			
	0	TD-OCT				
	11	SD-OCT				
	0	SS-OCT				
	11	NOS	0.0572	[−0.1058; 0.2202]	0.0832	0.4916
Inferior thickness	11	SSD participant age	0.0178	[−0.0165; 0.0521]	0.0175	0.3089
	11	Sex (% of male SSD participants)	0.0061	[−0.0044; 0.0166]	0.0053	0.2556
	6	Duration of illness (M)	0.0004	[−0.0015; 0.0023]	0.001	0.6979
	5	PANSS total score	−0.0051	[−0.0148; 0.0046]	0.005	0.3069
	3	PANSS positive scale score	—			
	3	PANSS negative scale score	—			
	3	PANSS general psychopathology scale score	—			
	2	Antipsychotic dose (chlorpromazine equivalent [mg/day])	—			
	4	Smoking (%)	—			
	2	Body mass index (kg/m^2^)	—			
	11	OCT device type	—			
	0	TD-OCT				
	11	SD-OCT				
	0	SS-OCT				
	11	NOS	0.085	[−0.0485; 0.2185]	0.0681	0.2122
Temporal thickness	10	SSD participant age	0.0386	[0.0025; 0.0747]	0.0184	0.036
	10	Sex (% of male SSD participants)	0.006	[−0.0071; 0.0191]	0.0067	0.3677
	6	Duration of illness (M)	0.0024	[−0.0008; 0.0056]	0.0017	0.1469
	5	PANSS total score	−0.0058	[−0.0179; 0.0064]	0.0062	0.3514
	3	PANSS positive scale score	—			
	3	PANSS negative scale score	—			
	3	PANSS General psychopathology scale score	—			
	2	Antipsychotic dose (chlorpromazine equivalent [mg/day])	—			
	4	Smoking (%)	—			
	2	Body mass index (kg/m^2^)	—			
	10	OCT device type	—			
	0	TD-OCT				
	10	SD-OCT				
	0	SS-OCT				
	10	NOS	0.0373	[−0.1375; 0.2121]	0.0892	0.6757
Nasal thickness	10	SSD participant age	0.0553	[0.0228; 0.0879]	0.0166	0.0009
	10	Sex (% of male SSD participants)	0.0002	[−0.0147; 0.0150]	0.0076	0.9818
	6	Duration of illness (M)	0.0041	[0.0010; 0.0072]	0.0016	0.009
	5	PANSS total score	−0.0068	[−0.0308; 0.0173]	0.0123	0.5827
	3	PANSS positive scale score	—			
	3	PANSS negative scale score	—			
	3	PANSS general psychopathology scale score	—			
	2	Antipsychotic dose (chlorpromazine equivalent [mg/day])	—			
	4	Smoking (%)	—			
	2	Body mass index (kg/m^2^)	—			
	10	OCT device type	—			
	0	TD-OCT				
	10	SD-OCT				
	0	SS-OCT				
	10	NOS	0.0337	[−0.1621; 0.2294]	0.0999	0.736
Outer ring						
Superior thickness	11	SSD participant age	−0.0015	[−0.0555; 0.0525]	0.0275	0.9562
	11	Sex (% of male SSD participants)	−0.0007	[−0.0174; 0.0160]	0.0085	0.9346
	5	Duration of illness (M)	−0.0016	[−0.0072; 0.0040]	0.0029	0.5859
	4	PANSS total score	—			
	2	PANSS positive scale score	—			
	2	PANSS negative scale score	—			
	2	PANSS general psychopathology scale score	—			
	2	Antipsychotic dose (chlorpromazine equivalent [mg/day])	—			
	2	Smoking (%)	—			
	1	Body mass index (kg/m^2^)	—			
	11	OCT device type	—			
	0	TD-OCT				
	11	SD-OCT				
	0	SS-OCT				
	11	NOS	0.0628	[−0.2111; 0.3366]	0.1397	0.6533
Inferior thickness	11	SSD participant age	0.0362	[−0.0063; 0.0788]	0.0217	0.0949
	11	Sex (% of male SSD participants)	0.001	[−0.0132; 0.0151]	0.0072	0.8955
	5	Duration of illness (M)	0.0019	[−0.0006; 0.0044]	0.0013	0.1276
	4	PANSS total score	—			
	2	PANSS positive scale score	—			
	2	PANSS negative scale score	—			
	2	PANSS general psychopathology scale score	—			
	2	Antipsychotic dose (chlorpromazine equivalent [mg/day])	—			
	2	Smoking (%)	—			
	1	Body mass index (kg/m^2^)	—			
	11	OCT device type	—			
	0	TD-OCT				
	11	SD-OCT				
	0	SS-OCT				
	11	NOS	0.1451	[−0.0663; 0.3565]	0.1079	0.1786
Temporal thickness	9	SSD participant age	0.0436	[0.0189; 0.0683]	0.0126	0.0005
	9	Sex (% of male SSD participants)	−0.0029	[−0.0156; 0.0099]	0.0065	0.6584
	5	Duration of illness (M)	0.0028	[0.0006; 0.0050]	0.0011	0.0113
	3	PANSS total score	—			
	2	PANSS positive scale score	—			
	2	PANSS negative scale score	—			
	2	PANSS general psychopathology scale score	—			
	2	Antipsychotic dose (chlorpromazine equivalent [mg/day])	—			
	2	Smoking (%)	—			
	1	Body mass index (kg/m^2^)	—			
	9	OCT device type	—			
	0	TD-OCT				
	9	SD-OCT				
	0	SS-OCT				
	9	NOS	0.0457	[−0.1936; 0.2850]	0.1221	0.7083
Nasal thickness	10	SSD participant age	0.0401	[−0.0020; 0.0822]	0.0215	0.062
	10	Sex (% of male SSD participants)	0.007	[−0.0083; 0.0223]	0.0078	0.3698
	5	Duration of illness (M)	0.002	[−0.0014; 0.0055]	0.0018	0.2462
	4	PANSS total score	—			
	2	PANSS positive scale score	—			
	2	PANSS negative scale score	—			
	2	PANSS general psychopathology scale score	—			
	2	Antipsychotic dose (chlorpromazine equivalent [mg/day])	—			
	3	Smoking (%)	—			
	1	Body mass index (kg/m^2^)	—			
	10	OCT device type	—			
	0	TD-OCT				
	10	SD-OCT				
	0	SS-OCT				
	10	NOS	0.0171	[−0.2596; 0.2937]	0.1411	0.9038
Macular GCL-IPL	9	SSD participant age	0.0054	[−0.0102; 0.0210]	0.008	0.4968
	9	Sex (% of male SSD participants)	0.0049	[−0.0136; 0.0233]	0.0094	0.607
	5	Duration of illness (M)	0.0012	[−0.0022; 0.0047]	0.0018	0.4923
	3	PANSS total score	—			
	3	PANSS positive scale score	—			
	3	PANSS negative scale score	—			
	3	PANSS general psychopathology scale score	—			
	5	Antipsychotic dose (chlorpromazine equivalent [mg/day])	0.0007	[−0.0017; 0.0030]	0.0012	0.5879
	3	Smoking (%)	—			
	4	Body mass index (kg/m^2^)	—			
	8	OCT device type	0.308	[−0.3258; 0.9419]	0.3234	0.3409
	0	TD-OCT				
	7	SD-OCT				
	1	SS-OCT				
	9	NOS	−0.0487	[−0.2010; 0.1036]	0.0777	0.5308

*CI* confidence interval, *GCL-IPL* ganglion cell layer–inner plexiform layer, *NOS* Newcastle–Ottawa Scale, *PANSS* Positive and Negative Symptom Scale, *SD-OCT* spectral domain–optical coherence tomography, *SS-OCT* swept source–optical coherence tomography, *TD-OCT* time domain–optical coherence tomography.

#### MT in ETDRS grid

We included 20 studies (1867 eyes of 1031 SSD participants and 1572 eyes of 872 HCs) in the MA for macular central foveal thickness (MCFT). Participants with SSDs had significantly thinner MCFT (Fig. S24). The LOO analysis showed that the difference remained significant for MCFT after removing each study one by one (Fig. S25). The GOSH analysis identified three outliers [60, 61, 67] (Fig. S26). After removing the outliers, the statistical significance remained for MCFT (Table S7). The counter-enhanced funnel plot and Egger’s regression test showed significant publication bias in MCFT (Fig. S27, Table S5). However, after adjusting for publication bias, the differences remained statistically significant (Table S5). The pooled estimate of MCFT was positively associated with SSD participant age and duration of illness (Table 2). On the other hand, we found a negative correlation between PANSS positive scale scores and pooled estimates of MCFT (Table 2). Results of the subgroup analysis indicated that the differences remained significant, even in the exclusion group (Fig. S28). We found no significant difference between the right and left eyes in the pooled estimate of MCFT (Fig. S29).

In the MA of superior, inferior, temporal, and nasal thickness in the outer ring of the macula, we included 11 studies (850 eyes of 503 SSD participants and 783 eyes of 460 HCs), 11 studies (850 eyes of 503 SSD participants and 783 eyes of 460 HCs), nine studies (687 eyes of 398 SSD participants and 663 eyes of 375 HCs), and ten studies (734 eyes of 445 SSD participants and 713 eyes of 425 HCs), respectively. All MTs in the outer ring were significantly thinner in participants with SSDs (Fig. S24). The LOO analysis showed that the difference remained significant after we removed each study for all MTs in the outer ring (Figs. S30–S33). In the superior, inferior, temporal, and nasal thickness in the outer ring, we identified one [60], one [60], one [67], and two outliers [61, 87], respectively (Fig. S34-37). After removing the outliers, statistical significance remained for the four segmental MTs in the outer ring (Table S7). The counter-enhanced funnel plot and Egger’s regression test showed no significant publication bias in the superior, inferior, and nasal thicknesses in the outer ring (Figs. S27, 38, Table S5). For temporal thickness in the outer ring, we did not perform Egger’s regression test because there were fewer than ten studies. Assuming the existence of publication bias, we performed the trim-and-fill method in the temporal thickness, and the differences remained statistically significant (Table S5). We found a positive correlation between SSD participant age and duration of illness and the pooled estimate of temporal thickness (Table 2). In all MTs in the outer ring, the subgroup analysis showed that differences remained significant, even in the exclusion group (Fig. S28). We found no significant difference in pooled estimates between the right and left eyes in all MTs in the outer ring (Fig. S29).

In the MA of superior, inferior, temporal, and nasal thickness in the inner ring of the macula, we included 11 studies (907 eyes of 536 SSD participants and 861 eyes of 500 HCs), 11 studies (907 eyes of 536 SSD participants and 861 eyes of 500 HCs), ten studies (721 eyes of 478 SSD participants and 729 eyes of 465 HCs), and ten studies (791 eyes of 478 SSD participants and 791 eyes of 465 HCs), respectively. We observed a significant thinning in SSD participants for all MTs in the inner ring (Fig. S2). In the LOO analysis, the statistical significance remained after we removed each study for all MTs in the inner ring (Figs. S39-42). In the superior, inferior, temporal, and nasal thickness in the inner ring, we identified one [60], one [60], one [61], and two outliers [61, 67], respectively (Figs. S43-46). After we removed the outliers, the statistical significance remained for four segmental thicknesses in the inner ring (Table S7). The counter-enhanced funnel plot and Egger’s regression test showed no significant publication bias in all MTs in the inner ring (Figs. S38, 47, Table S7). There was a positive association between SSD participant age, duration of illness, and the pooled estimate of the macular inner nasal thickness, in addition to the same association between SSD participant age and overall effect size of macular inner temporal thickness (Table 2). For MT in four segments of the inner ring, the results of the subgroup analyses revealed that the differences remained significant, even in the exclusion group (Fig. S28). In MT in four segments of the inner ring, we observed no significant differences in the pooled estimates between the right and left eyes (Fig. S29). Due to high heterogeneity and publication bias for MCFT and high heterogeneity for all MTs in the outer and inner rings, the GRADE rating results were “very low” (Table S6).

#### mGCL-IPL thickness

Nine studies (1384 eyes of 808 SSD participants and 165,974 eyes of 101,219 HCs) were included in the MA for mGCL-IPL. The mGCL-IPL thickness was thinner in participants with SSDs (Fig. 2). The LOO analysis revealed that significant differences remained after each study was omitted (Fig. S48). We identified one outlier [14] (Fig. S49). After the outlier was removed, a significant difference remained (Table S8). Fig. S47 shows the counter-enhanced funnel plot. Significant differences remained after adjusting for publication bias (Table S5). None of the explanatory variables (*N* ≥ 5) was associated with the pooled estimate of mGCL-IPL thickness (Table 2). Although only two studies excluded diabetes and hypertension, the subgroup analyses revealed that the difference remained significant, even in the exclusion group (Fig. 3). We also observed no significant differences in the pooled estimates between the right and left eyes (Fig. S50). The GRADE rating results were “very low” due to high heterogeneity for mGCL-IPL (Table S6).

#### Optic cup

We included five studies (306 eyes of 164 SSD participants and 258 eyes of 142 HCs) and four (540 eyes of 270 SSD participants and 532 eyes of 266 HCs) in the MA, respectively, for CDR and OCV. OCV was significantly enlarged in participants with SSDs (Fig. 2). On the other hand, no significant difference in CDR was found between SSD participants and HCs (Fig. S16). In OCV, the LOO analysis revealed significant differences were lost after omitting the study by Jerotic et al. [63] or the study by Liu et al. [65] (Fig. S51). No outliers were detected in both CDR and OCV (Fig. S52). We show the counter-enhanced funnel plots in CDR and OCV in Fig. S52. In OCV, significant differences diminished after adjusting for publication bias (Table S5). We did not meta-regression due to the small number of studies included in the MA of CDR and OCV. The difference remained significant, even in the exclusion group in CDR (Fig. S53). Subgroup analyses were not performed in OCV because the number of studies is 2 in both the exclusion and non-exclusion groups. No significant differences in pooled estimates between the right and left eyes were observed in CDR and OCV (Fig. S50). The GRADE rating result was “very low” due to high heterogeneity for OCV (Table S6).

### OCTA findings

Table S2 shows the main findings of the OCTA studies included in the SR. We included OCTA studies with a total of 488 eyes of 320 participants (148 SSD participants and 172 HCs) in the MA (Fig. 1). In the MA of the FAZ and superficial foveal VD, we included three studies (202 eyes of 136 SSD participants and 231 eyes of 157 HCs) and three studies (118 eyes of 83 SSD participants and 150 eyes of 100 HCs), respectively. The FAZ and superficial foveal VD were not significantly different between SSD participants and HCs (Fig. S54). GOSH identified no outlier (Table S9). Figure S55 shows the counter-enhanced funnel plots. There was no change in the results after adjusting for publication bias (Table S5).

### Findings of photopic and scotopic fERG

**Fig. 4:**
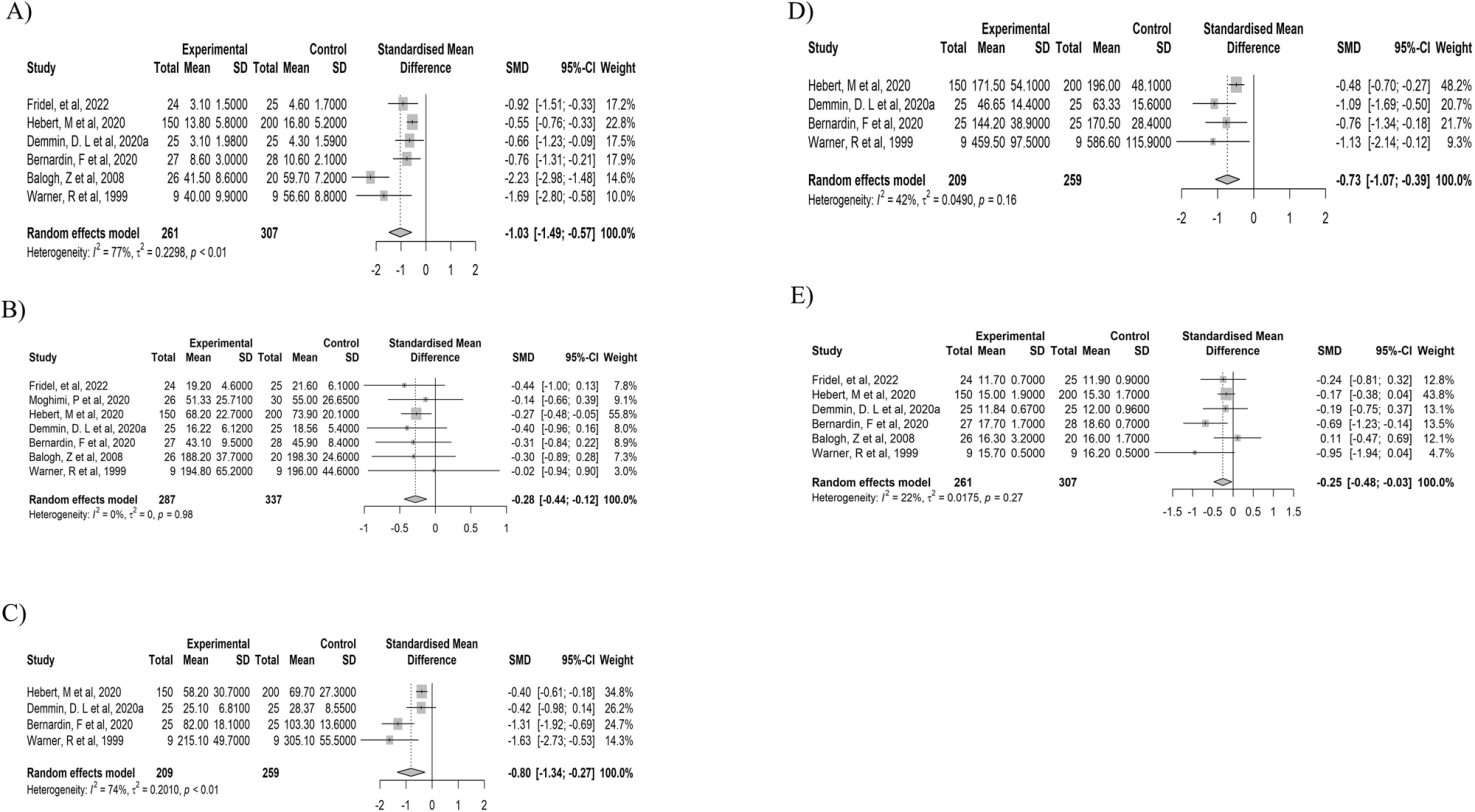
Results of the meta-analysis of fERG in the photopic and scotopic conditions. **A** The a-wave amplitude of photopic fERG. **B** The b-wave amplitude of photopic fERG. **C** The a-wave amplitude of scotopic fERG. **D** The b-wave amplitude of scotopic fERG. **E** The a-wave latency of photopic fERG. Horizontal bars indicate 95% confidence intervals (95% CIs). Total indicates the total number of participants for which the mean and standard deviation were calculated. fERG flash electroretinography, SMD standardized mean difference, SD standard deviation.

Table S3 shows the main findings of the fERG studies included in the SR. Seven studies (311 SSD participants and 362 HCs) were included in the MA of fERG in the photopic and scotopic conditions (Fig. 1). In the a-wave amplitude of photopic and scotopic fERG, we included six studies (261 SSD participants and 307 HCs) and four studies (209 SSD participants and 259 HCs), respectively. The amplitude of the a-wave was significantly reduced in SSD participants in photopic and scotopic fERG (Fig. 4). In the a-wave amplitude of both conditions, no significant differences were lost after we removed each study in the LOO analysis (Figs. S56, 57). Outliers were not identified in the GOSH analysis (Table S10). After we adjusted for publication bias, significant differences remained (Fig. S58, Table S11). In the photopic a-wave amplitude, we found no association between age, sex, and overall effect size (Table S12). In the b-wave amplitude of photopic and scotopic fERG, we included seven studies (287 SSD participants and 337 HCs) and four studies (209 SSD participants and 259 HCs), respectively. The b-wave amplitude was significantly reduced in participants with SSDs in photopic and scotopic fERG (Fig. 4). In the b-wave amplitude of both conditions, the significant differences remained after we removed each study in the LOO analysis (Figs. S59, 60). Outliers were not identified in the GOSH analysis (Table S10). Significant differences remained after adjusting for publication bias (Table S11). There was no association between age, sex, and pooled estimates of photopic b-wave amplitude (Table S12). The GRADE rating results were “moderate” to “low” for a- and b-wave amplitudes in photopic and scotopic fERG (Table S6). In PhNR amplitude, we observed no significant differences between SSD participants and HCs. In photopic and scotopic a-wave latency time, we included six studies (261 SSD participants and 307 HCs) and four studies (209 SSD participants and 259 HCs), respectively. Photopic a-wave latency time was significantly shorter in participants with SSDs (Fig. 4). We found no significant differences in photopic b-wave and scotopic a- and b-wave latency time (Fig. S61). In photopic a-wave latency time, the LOO analysis revealed that the significant difference was lost after we omitted the study by Fridel et al. [56] (Fig. S62), and the GOSH analysis identified no outliers (Table S10). After adjusting for publication bias, we found that the difference was no longer significant (Fig. S63, Table S11). We found no association between any of the explanatory variables (*N* ≥ 5) and the pooled estimate of photopic a-wave latency time (Table S12). The GRADE rating result was “low” for photopic a-wave latency time (Table S6).

## Discussion

The MA for retinal thickness, which includes more studies than any previously reported MA, shows that SSD participants have thinning of pRNFL average thickness, pRNFL thicknesses in all four quadrants, MAT, MTs in all sectors of the ETDRS grid, and mGCL-IPL, in addition to reduced MV, and enlarged optic cup values. In addition, the exclusion of participants with diabetes and hypertension did not change the results, except in the case of pRNFL inferior and nasal thickness. Furthermore, there was a significant difference between the right and left eye in pRNFL temporal thickness, although, for the most part, there is not asymmetry. There was a positive correlation between disease duration and MAT, in contrast to a negative association between disease duration and pRNFL average thickness. Also, a negative correlation was found between the severity of positive and negative symptoms, and MCFT and pRNFL superior thickness, respectively. The MA for fERG revealed that SSD participants had reduced amplitudes of light- and dark-adaptive a- and b-waves and a shortened light-adaptive a-wave latency.

Although the mechanism of retinal thinning remains unclear in SSD participants, several hypotheses have been postulated. One hypothesis is retrograde transsynaptic degeneration [5, 128, 209]. In rodents with damaged occipital lobes, neurodegeneration of the retina has been reported to occur after degeneration of neurons in the lateral geniculate nucleus of the thalamus that project to V1, and abnormalities in the structure and function of the occipital lobes and thalamus have been reported in schizophrenia participants [210, 211]. In a more recent study involving a larger sample size, participants with psychosis showed significant reductions in area, thickness, and volume in the primary visual area (Brodmann area 17/V1), secondary visual area (Brodmann area 18/V2), and middle temporal (V5/MT) region, with gender-dependent changes in area and volume in V1 and V2 areas (i.e., reduction in area and volume of these regions limited to female probands) [212]. In a follow-up study of untreated participants with first-episode schizophrenia presenting with visual impairment, the authors found a significant correlation between decreased volume of gray matter in the visual cortex and retinal thinning [69]. However, not all studies reported that participants with schizophrenia have structural abnormalities in the occipital lobe or thalamus, which raises questions that cannot be explained by this hypothesis alone [213]. Another hypothesis is that amacrine cells in the retina, which synapse with ganglion cells in the inner reticular formation, synthesize and release dopamine [214], and that changes in dopamine signaling between amacrine cells and ganglion cells may cause ganglion cell damage, which is reportedly caused by changes in dopamine signaling between amacrine cells and ganglion cells [10]. An additional consideration is the potential link between these observed effects and the process of excessive synaptic pruning [215]. This phenomenon, known for its association with the reduction of cortical grey matter in schizophrenia, could underlie some of the retinal changes. Another possibility is that the genetic factors involved in the risk of developing schizophrenia affect the brain and retina, which are the same embryologically and have shared functional and structural characteristics. This genetic linkage is supported by genome-wide single nucleotide polymorphisms identified in the whole genome association analysis of macular retinal thickness that were at risk for developing schizophrenia [216]. Future neuro-retinal imaging genetics studies to identify the shared genetic basis for brain volume reduction and retinal thinning in SSD participants may be useful in elucidating the pathogenesis of retinal thinning. Also, further investigation into these genetic markers could provide deeper insights into both the development of schizophrenia and the correlated retinal changes. The discovery of common genetic pathways between the retina and brain may open new avenues for early detection and targeted therapies in schizophrenia spectrum disorders. Despite these promising leads, our understanding of the underlying mechanisms for retinal cell loss in psychosis is, at this stage, largely theoretical and requires further investigation.

It is not clear when retinal thinning occurs, i.e., whether SSD participants have an inherently thinner retina or whether retinal thinning occurs after the onset of the disease. A study by Kurtulmus et al., reported that even unaffected first-degree relatives of participants with schizophrenia show retinal thinning compared to HCs, suggesting that retinal thinning is a trait marker [74]. Given the preliminary evidence of retinal thinning in unaffected first-degree relatives, as well as the evidence that OCT findings are related to the level of symptoms, OCT findings show characteristics of both vulnerability markers and episode markers may thus represent mediating vulnerability markers [217]. Recently, a longitudinal study investigated whether macular retinal thickness is related to the risk of developing SSDs [54]. Interestingly, the study found no association between macular retinal thickness and the development of SSDs. It should be noted, however, that the participants in the study were in their 40 s, which is not typically the age of onset of schizophrenia. As such, this age discrepancy might have affected the findings. On the other hand, thinning of the retina has been reported in participants with first-episode untreated schizophrenia with visual disturbances [69], and a decrease in retinal thickness was observed for a period of time after treatment, suggesting that thinning of retinal thickness may reflect both an early onset of pathological processes and the effects of antipsychotic treatment in specific subtypes of schizophrenia. On the other hand, Lai et al. reported no significant differences in macular retinal thickness and pRNFL thickness among age-matched participants with first psychotic episodes and HCs, with the exclusion of diabetes and hypertension. The results suggest that retinal structure is not affected early in the onset of SSDs [75]. However, Lai and colleagues’ study should be interpreted with caution due to the relatively small number of cases and unmatched sexes. Of note, however, a follow-up study observed significant atrophy in the retinal microvasculature in the same first episode participants [25], suggesting evidence of retinal changes at the first episode, and possibly a sequence wherein vascular changes precede neural changes. To determine whether thinning of the retina reflects the pathological process at the onset of schizophrenia or the effect of treatment, a longitudinal study based on young adults in the age at which schizophrenia occurs may be warranted to examine retinal thickness before and after the onset of schizophrenia. We found a negative correlation between estimates of pRNFL thickness and duration of illness. This finding suggests that the degree of thinning of pRNFL thickness in SSD participants increase with the duration of illness compared to HCs, which supports the hypothesis that schizophrenia is a neurodegenerative disease. On the other hand, unexpectedly, MT was positively correlated with the duration of illness. Different from the peripapillary region, the macula is an area with a high density of neuronal cells. Therefore, the results suggest that neuronal loss in the macular retina is more pronounced in the early stages of disease onset, followed by a slowing of the degree of loss due to treatment and other factors. This pattern emphasizes the dynamic nature of the disease, which is in line with contemporary thinking about the course of schizophrenia [218]. Another potential explanation is that increased macular thickness could result from macular edema, which is seen in diseases such as diabetic retinopathy and age-related macular degeneration [219, 220]. Thus, the positive correlation between MT and the duration of illness may suggest the presence of a progressive condition, such as macular edema, that intensifies with longer disease duration rather than indicating a slowing of neuronal loss. However, this is a very tentative hypothesis as there are no prior studies reporting that older individuals with SSD have more edema than younger individuals with SSD. A third possible explanation is based on the fact that individuals with schizophrenia have a life expectancy approximately 15–20 years shorter than that of non-psychiatrically ill peers [221]. Much of this premature mortality is due to diseases such as cardiovascular disease and diabetes, which could exacerbate macular thinning. This means that older SSD individuals who, if they lived, would have a thinner macula are dying due to comorbid medical conditions. Thus, those who are still alive (and participate in studies) are, on average, physically healthier than younger individuals with SSD. A fourth explanation is that older SSD individuals have been treated with first-generation antipsychotics (e.g., Haloperidol), which have fewer metabolic side effects. Many of these individuals will have been maintained on these antipsychotics (if effective) as they aged. In contrast, younger individuals with SSD are more likely to have been initially treated with second-generation antipsychotics, which we have major metabolic side effects (weight/body mass index gain, diabetes, etc.) [222, 223] associated with macular thinning. In other words, it could be that older individuals have thicker MT simply because they have had lower exposure to a class of medications likely to have caused macula thinning. On the other hand, more recent studies have accelerated age-related decline in SSD participants compared to HCs [23, 224, 225]. Further studies are needed to clarify the discrepancy and to examine how the retinal findings vary with age and disease duration (e.g., are these relationships linear or non-linear, or do they vary over decades).

A prior study reported that significant differences in retinal thinning in schizophrenia participants disappeared when hypertension and diabetes were excluded [21]. However, we found that most retinal thicknesses were significantly thinner in SSD participants, even in an MA, including only studies that excluded hypertension and diabetes. We also found significant differences between the left and right eyes in pRNFL temporal thickness. This result indicates the importance of bilateral analysis when comparing retinal thickness between SSD participants and HCs.

Several studies have reported abnormalities of VD in the peripapillary and macula region in SSD participants [24, 25, 58, 64, 70]. However, the results are inconsistent. Silverstein et al. reported lower VD in the superficial vascular layer at the macula in the left eye in SSD participants [25]. On the other hand, Bannai et al. reported higher superficial skeletonized VD (SVD), choriocapillaris VD, and choriocapillaris SVD in the macular region, and in participants with disease duration <5 years, higher superficial VD, choriocapillaris SVD, and choriocapillaris fractal dimension in the right eye [24]. These discrepancies may be due to differences in the area of measurement for VD, as well as in the characteristics of the participants, but further investigation is required. Although the number of studies included in the MA is limited, the present study revealed no significant difference between SSD participants and HCs in FAZ and superficial foveal VD. Budakolglu et al. reported thinning of the pRNFL temporal thickness and decreased VD in the same area [70]. Silverstein et al. also reported a significant positive correlation between macular PD and central retinal thickness [25], and MV. These results suggest that retinal neural changes in SSD participants reflect microvascular abnormalities.

The a- and b-waves amplitudes were significantly attenuated in SSD participants. Further, we also revealed a shortened a-wave latency under photopic conditions in participants with SSDs. Fridel et al. found a reduction in fERG a-wave amplitude and thinning of the outer nuclear layer (ONL) in participants with schizophrenia, and a significant positive correlation between a-wave amplitude and ONL thickness [56]. The findings suggest that structural changes in the retina partially contribute to the reduced a-wave amplitude. Furthermore, b-waves reflect bipolar cell functions, and thinning of the inner nuclear layer containing the cell bodies of bipolar cells has been previously reported in SSD participants [78], which may contribute to b-wave attenuation. Ultimately, the intricate connections between functional loss and structural changes in the retina are yet to be fully understood. The dynamic interactions between variations in the photoreceptor layer and higher-order structures such as mGCL-IPL [56], along with their correlations with distinct dopaminergic states in different stages (acute vs. chronic) and proposed classifications (hyperdopaminergic vs. normodopaminergic) [226], add layers of complexity that keep this subject an open and intriguing question. These observed changes may not merely reflect neuronal degeneration tied to the disease but could also be indicative of early markers or factors predisposing to the condition.

Retinal structure and function can be measured noninvasively, making these potentially useful biological indicators for predicting prognosis and functional decline, and assessing treatment response. However, interpretation of retinal data can be complicated due to the increase of various confounding factors (e.g., smoking, metabolic factors, etc.) with aging, and the association of these factors (as well as others such as sleep disturbance) with SSDs. Therefore, it may be useful to conduct longitudinal studies in young adults or adolescents with at-risk mental states and with first-episode psychosis to examine the applicability of these measures for predicting the transition to psychosis and treatment response, and for assessing the severity of symptoms and cognitive dysfunction. Furthermore, most of the studies so far have been using only either OCT or ERG, and therefore, a multimodal approach that simultaneously measures OCT, OCTA, and fERG, combined with other genetic factors and neuroimaging findings, may accelerate the understanding of the pathology underlying retinal abnormality and the development of more accurate prediction models for prognosis, treatment response, and neurodegeneration in the brain, cognitive decline, and decline in real-world functioning. In addition, studies have also reported the association between other psychiatric disorders, such as bipolar disorder [8, 227, 228], major depressive disorders [229], autism spectrum disorders [230], attention-deficit/hyperactivity disorder [231, 232], and retinal thinning. To clarify the pathophysiology of shared retinal thinning across several psychiatric disorders, a dimensional approach examining the association between clinical features common in all psychiatric disorders (e.g., cognitive impairment) and retinal thinning would be useful.

This study has several limitations. First, because there were fewer OCTA and fERG studies relative to OCT studies, we were unable to assess associations with clinical indicators such as psychiatric symptoms for these variables in the meta-regression analysis. Furthermore, we did not perform a subgroup analysis in OCTA and fERG studies. The small number of studies, especially for OCTA, suggests that further studies and an MA including more studies would be needed to draw conclusions. Second, we excluded from the MA studies that did not include necessary numerical data for the MA.

In conclusion, the study revealed that pRNFL thickness and retinal thickness in macular regions were thinner in SSD participants, even after excluding the effects of hypertension and diabetes. Furthermore, the fERG a- and b-waves amplitude in photopic and scotopic conditions was attenuated, and the latency of the a-wave in photopic conditions was shortened. These results suggest that functional and structural abnormalities in the retina may be potential state/trait markers for predicting prognosis, assessing treatment response, and severity of disease in SSD participants. Future longitudinal multimodal neuro-retinal imaging genetics studies are needed to clarify the pathological mechanisms of retinal abnormalities and to establish the retina as a state/trait marker.

### Supplementary information

Supplementary information

## Data Availability

The data supporting the findings of this study are available from the corresponding author, HK, upon reasonable request.

## References

[1] Komatsu H, Takeuchi H, Kikuchi Y, Ono C, Yu Z, Iizuka K (2020). Ethnicity-Dependent Effects of Schizophrenia Risk Variants of the OLIG2 Gene on OLIG2 Transcription and White Matter Integrity. Schizophr Bull.

[2] Mauschitz MM, Lohner V, Koch A, Stöcker T, Reuter M, Holz FG (2022). Retinal layer assessments as potential biomarkers for brain atrophy in the Rhineland Study. Sci Rep.

[3] Mutlu U, Bonnemaijer PWM, Ikram MA, Colijn JM, Cremers LGM, Buitendijk GHS (2017). Retinal neurodegeneration and brain MRI markers: the Rotterdam Study. Neurobiol Aging.

[4] Pan J, Zhou Y, Xiang Y, Yu J (2018). Retinal nerve fiber layer thickness changes in Schizophrenia: A meta-analysis of case-control studies. Psychiatry Res.

[5] Lizano P, Bannai D, Lutz O, Kim LA, Miller J, Keshavan M (2020). A Meta-analysis of Retinal Cytoarchitectural Abnormalities in Schizophrenia and Bipolar Disorder. Schizophr Bull.

[6] Kazakos CT, Karageorgiou V (2020). Retinal Changes in Schizophrenia: A Systematic Review and Meta-analysis Based on Individual Participant Data. Schizophr Bull.

[7] Gonzalez-Diaz JM, Radua J, Sanchez-Dalmau B, Camos-Carreras A, Zamora DC, Bernardo M (2022). Mapping Retinal Abnormalities in Psychosis: Meta-analytical Evidence for Focal Peripapillary and Macular Reductions. Schizophr Bull.

[8] Prasannakumar A, Kumar V, Mailankody P, Appaji A, Battu R, Berendschot TT, et al. A systematic review and meta-analysis of Optical coherence tomography studies in Schizophrenia, Bipolar disorder and Major depressive disorder. World J Biol Psychiatry. 2023;24:1–16. 10.1080/15622975.2023.2203231 37070475

[9] Komatsu H, Onoguchi G, Jerotic S, Kanahara N, Kakuto Y, Ono T (2022). Retinal layers and associated clinical factors in schizophrenia spectrum disorders: a systematic review and meta-analysis. Mol Psychiatry.

[10] Samani NN, Proudlock FA, Siram V, Suraweera C, Hutchinson C, Nelson CP (2018). Retinal Layer Abnormalities as Biomarkers of Schizophrenia. Schizophr Bull.

[11] Chu EMY, Kolappan M, Barnes TRE, Joyce EM, Ron MA (2012). A window into the brain: An in vivo study of the retina in schizophrenia using optical coherence tomography. Psychiatry Res-Neuroimaging.

[12] Kaya H, Ayık B, Tasdelen R, Sevimli N, Ertekin E (2022). Comparing retinal changes measured by optical coherence tomography in patients with schizophrenia and their siblings with healthy controls: Are retinal findings potential endophenotype candidates?. Asian J Psychiatr.

[13] Khalil DH, Aziz K, Khalil M, Khowyled A (2022). Optical coherence tomography in Egyptian schizophrenics and its correlation to disease parameters. Delta J Ophthalmol.

[14] Boudriot E, Schworm B, Slapakova L, Hanken K, Jäger I, Stephan M, et al. Optical coherence tomography reveals retinal thinning in schizophrenia spectrum disorders. Eur Arch Psychiatry Clin Neurosci. 2023;273:575–88. 10.1007/s00406-022-01455-z PMC10085905 35930031

[15] Alizadeh M, Delborde Y, Ahmadpanah M, Seifrabiee MA, Jahangard L, Bazzazi N (2021). Non-linear associations between retinal nerve fibre layer (RNFL) and positive and negative symptoms among men with acute and chronic schizophrenia spectrum disorder. J Psychiatr Res.

[16] Carriello MA, Costa DFB, Alvim PHP, Pestana MC, Bicudo DDS, Gomes EMP, et al. Retinal layers and symptoms and inflammation in schizophrenia. Eur Arch Psychiatry Clin Neurosci 2023. Online ahead of print. 10.1007/s00406-023-01583-0 36928482

[17] Dervişoğulları MS, Totan Y, Tenlik A, Yüce A, Güler E (2015). Effect of smoking on retina nerve fiber layer and ganglion cell-inner plexiform layer complex. Cutan Ocul Toxicol.

[18] Teberik K (2019). The Effect of Smoking on Macular, Choroidal, and Retina Nerve Fiber Layer Thickness. Turk J Ophthalmol.

[19] Xie H, Pan Z, Xue CC, Chen D, Jonas JB, Wu X, et al. Arterial hypertension and retinal layer thickness: the Beijing Eye Study. Br J Ophthalmol. 2022. Online ahead of print. 10.1136/bjo-2022-322229 36428008

[20] Shahidi AM, Sampson GP, Pritchard N, Edwards K, Vagenas D, Russell AW (2012). Retinal nerve fibre layer thinning associated with diabetic peripheral neuropathy. Diabet Med.

[21] Silverstein SM, Paterno D, Cherneski L, Green S (2018). Optical coherence tomography indices of structural retinal pathology in schizophrenia. Psychol Med.

[22] Green KM, Choi JJ, Ramchandran RS, Silverstein SM (2022). OCT and OCT Angiography Offer New Insights and Opportunities in Schizophrenia Research and Treatment. Front Digit Health.

[23] Wagner SK, Cortina-Borja M, Silverstein SM, Zhou Y, Romero-Bascones D, Struyven RR, et al. Association Between Retinal Features From Multimodal Imaging and Schizophrenia. JAMA Psychiatry. 2023;80:478–87. 10.1001/jamapsychiatry.2023.0171 PMC10034669 36947045

[24] Bannai D, Adhan I, Katz R, Kim LA, Keshavan M, Miller JB (2022). Quantifying Retinal Microvascular Morphology in Schizophrenia Using Swept-Source Optical Coherence Tomography Angiography. Schizophr Bull.

[25] Silverstein SM, Lai A, Green KM, Crosta C, Fradkin SI, Ramchandran RS (2021). Retinal Microvasculature in Schizophrenia. Eye Brain.

[26] Appaji A, Nagendra B, Chako DM, Padmanabha A, Jacob A, Hiremath CV (2019). Retinal vascular tortuosity in schizophrenia and bipolar disorder. Schizophr Res.

[27] Appaji A, Nagendra B, Chako DM, Padmanabha A, Jacob A, Hiremath CV (2019). Examination of retinal vascular trajectory in schizophrenia and bipolar disorder. Psychiatry Clin Neurosci.

[28] Hua J, Brandt AS, Lee S, Blair NIS, Wu Y, Lui S (2017). Abnormal Grey Matter Arteriolar Cerebral Blood Volume in Schizophrenia Measured With 3D Inflow-Based Vascular-Space-Occupancy MRI at 7T. Schizophr Bull.

[29] Katsel P, Roussos P, Pletnikov M, Haroutunian V (2017). Microvascular anomaly conditions in psychiatric disease. Schizophrenia - angiogenesis connection. Neurosci Biobehav Rev.

[30] Harris LW, Wayland M, Lan M, Ryan M, Giger T, Lockstone H (2008). The cerebral microvasculature in schizophrenia: a laser capture microdissection study. PLoS One.

[31] Kreczmanski P, Schmidt-Kastner R, Heinsen H, Steinbusch HW, Hof PR, Schmitz C (2005). Stereological studies of capillary length density in the frontal cortex of schizophrenics. Acta Neuropathol.

[32] Park JJ, Oh DR, Hong SP, Lee KW (2005). Asymmetry analysis of the retinal nerve fiber layer thickness in normal eyes using optical coherence tomography. Korean J Ophthalmol.

[33] Quach J, Sharpe GP, Demirel S, Girkin CA, Mardin CY, Scheuerle AF (2023). Asymmetry of Peripapillary Retinal Blood Vessel and Retinal Nerve Fiber Layer Thickness Between Healthy Right and Left Eyes. Investig Ophthalmol Vis Sci.

[34] Hosák L, Zeman T, Studnička J, Stepanov A, Ustohal L, Michalec M (2020). Retinal arteriolar and venular diameters are widened in patients with schizophrenia. Psychiatry Clin Neurosci.

[35] Schijven D, Postema MC, Fukunaga M, Matsumoto J, Miura K, de Zwarte SMC (2023). Large-scale analysis of structural brain asymmetries in schizophrenia via the ENIGMA consortium. Proc Natl Acad Sci USA.

[36] Okada N, Fukunaga M, Yamashita F, Koshiyama D, Yamamori H, Ohi K (2016). Abnormal asymmetries in subcortical brain volume in schizophrenia. Mol Psychiatry.

[37] Bannai D, Lizano P, Kasetty M, Lutz O, Zeng V, Sarvode S (2020). Retinal layer abnormalities and their association with clinical and brain measures in psychotic disorders: A preliminary study. Psychiatry Res Neuroimaging.

[38] Demmin DL, Davis Q, Roché M, Silverstein SM (2018). Electroretinographic anomalies in schizophrenia. J Abnorm Psychol.

[39] Balogh Z, Benedek G, Kéri S (2008). Retinal dysfunctions in schizophrenia. Prog Neuropsychopharmacol Biol Psychiatry.

[40] Hébert M, Mérette C, Gagné AM, Paccalet T, Moreau I, Lavoie J (2020). The Electroretinogram May Differentiate Schizophrenia From Bipolar Disorder. Biol Psychiatry.

[41] Page MJ, McKenzie JE, Bossuyt PM, Boutron I, Hoffmann TC, Mulrow CD (2021). The PRISMA 2020 statement: an updated guideline for reporting systematic reviews. Bmj.

[42] Stang A. Critical evaluation of the Newcastle-Ottawa scale for the assessment of the quality of nonrandomized studies in meta-analyses. Eur J Epidemiol. 2010;25:603–5. 10.1007/s10654-010-9491-z 20652370

[43] Guyatt GH, Oxman AD, Vist GE, Kunz R, Falck-Ytter Y, Alonso-Coello P (2008). GRADE: an emerging consensus on rating quality of evidence and strength of recommendations. Bmj.

[44] Xu Q, Li Y, Cheng Y, Qu Y (2018). Assessment of the effect of age on macular layer thickness in a healthy Chinese cohort using spectral-domain optical coherence tomography. BMC Ophthalmol.

[45] DerSimonian R, Kacker R (2007). Random-effects model for meta-analysis of clinical trials: an update. Contemp Clin Trials.

[46] Viechtbauer W, Cheung MW (2010). Outlier and influence diagnostics for meta-analysis. Res Synth Methods.

[47] Olkin I, Dahabreh IJ, Trikalinos TA (2012). GOSH - a graphical display of study heterogeneity. Res Synth Methods.

[48] Egger M, Davey Smith G, Schneider M, Minder C (1997). Bias in meta-analysis detected by a simple, graphical test. Bmj.

[49] Duval S, Tweedie R (2000). Trim and fill: A simple funnel-plot-based method of testing and adjusting for publication bias in meta-analysis. Biometrics.

[50] Balduzzi S, Rücker G, Schwarzer G (2019). How to perform a meta-analysis with R: a practical tutorial. Evid Based Ment Health.

[51] Viechtbauer W (2010). Conducting Meta-Analyses in R with the metafor Package. J Stat Softw.

[52] Harrer M, Cuijpers P, Furukawa T, Ebert D. Doing Meta-Analysis with R: A Hands-On Guide. 1st ed. 2021. p. 500. 10.1201/9781003107347.

[53] Kurtulmus A, Sahbaz C, Elbay A, Guler EM, Sonmez Avaroglu G, Kocyigit A et al. Clinical and biological correlates of optical coherence tomography findings in schizophrenia. Eur Arch Psychiatry Clin Neurosci. 2023. Online ahead of print. 10.1007/s00406-023-01587-w 37022475

[54] Shoham N, Lewis G, Hayes JF, Silverstein SM, Cooper C (2023). Association between visual impairment and psychosis: A longitudinal study and nested case-control study of adults. Schizophr Res.

[55] Bozali E, Yalinbas D (2022). Analysis of the Thickness of the Outer Retinal Layer Using Optical Coherence Tomography - A Predictor of Visual Acuity in Schizophrenia. Klin Monbl Augenheilkd.

[56] Friedel EBN, Hahn HT, Maier S, Küchlin S, Reich M, Runge K (2022). Structural and functional retinal alterations in patients with paranoid schizophrenia. Transl Psychiatry.

[57] Kango A, Grover S, Gupta V, Sahoo S, Nehra R. A comparative study of retinal layer changes among patients with schizophrenia and healthy controls. Acta Neuropsychiatr. 2022;35:1–12. 10.1017/neu.2022.35 36476516

[58] Kokacya MH, Cakmak AI (2022). Optical Coherence Tomography Angiography in Schizophrenia. Alpha Psychiatry.

[59] Kurt A, Zor KR, Kucuk E, Yildirim G, Ersan EE (2022). An Optical Coherence Tomography Study that Supports the Neurovascular Basis of Schizophrenia Disease. Alpha Psychiatry.

[60] Asanad S, O’Neill H, Addis H, Chen S, Wang J, Goldwaser E (2021). Neuroretinal Biomarkers for Schizophrenia Spectrum Disorders. Transl Vis Sci Technol.

[61] Altun IK, Turedi N, Aras N, Atagun MI (2020). Psychopharmacological Signatures in the Retina in Schizophrenia and Bipolar Disorder: An Optic Coherence Tomography Study. Psychiatr Danub.

[62] Gandu S, Bannai D, Adhan I, Kasetty M, Katz R, Zang R, et al. Inter-device reliability of swept source and spectral domain optical coherence tomography and retinal layer differences in schizophrenia. Biomark Neuropsychiatr. 2021;5:100036.

[63] Jerotic S, Lalovic N, Pejovic S, Mihaljevic M, Pavlovic Z, Britvic D (2021). Sex differences in macular thickness of the retina in patients with psychosis spectrum disorders. Prog Neuropsychopharmacol Biol Psychiatry.

[64] Koman-Wierdak E, Róg J, Brzozowska A, Toro MD, Bonfiglio V, Załuska-Ogryzek K, et al. Analysis of the Peripapillary and Macular Regions Using OCT Angiography in Patients with Schizophrenia and Bipolar Disorder. J Clin Med. 2021;10:4131. 10.3390/jcm10184131 PMC8472507 34575242

[65] Liu Y, Chen J, Huang L, Yan S, Bian Q, Yang F (2021). Relationships Among Retinal Nerve Fiber Layer Thickness, Vascular Endothelial Growth Factor, and Cognitive Impairment in Patients with Schizophrenia. Neuropsychiatr Dis Treat.

[66] Murav’eva SV, Kozub KE, Pronin SV (2021). Optical and electrophysiological techniques for functional assessment of vision system neuronal networks. J Optical Technol.

[67] Sarkar S, Rajalakshmi AR, Avudaiappan S, Eswaran S (2021). Exploring the role of macular thickness as a potential early biomarker of neurodegeneration in acute schizophrenia. Int Ophthalmol.

[68] Zhuo C, Xiao B, Chen C, Jiang D, Li G, Ma X (2021). Abberant inverted U-shaped brain pattern and trait-related retinal impairment in schizophrenia patients with combined auditory and visual hallucinations: a pilot study. Brain Imaging Behav.

[69] Zhuo C, Xiao B, Ji F, Lin X, Jiang D, Tian H (2021). Patients with first-episode untreated schizophrenia who experience concomitant visual disturbances and auditory hallucinations exhibit co-impairment of the brain and retinas-a pilot study. Brain Imaging Behav.

[70] Budakoglu O, Ozdemir K, Safak Y, Sen E, Taskale B (2021). Retinal nerve fibre layer and peripapillary vascular density by optical coherence tomography angiography in schizophrenia. Clin Exp Optom.

[71] Huang JJ, Song XQ, Xu Y, Wang LN, Li YC, Tian HJ (2020). Reliability and Diagnostic Validity of A Novel Visual Disturbance Subjective Experience Scale in Chinese Patients with Schizophrenia. Psychiatry Clin Psychopharmacol.

[72] Jerotic S, Ristic I, Pejovic S, Mihaljevic M, Pavlovic Z, Britvic D (2020). Retinal structural abnormalities in young adults with psychosis spectrum disorders. Prog Neuropsychopharmacol Biol Psychiatry.

[73] Kozub KE, Shelepin IE, Chomskii AN, Sharybin EA, Ivanova EA. A structural and functional study of the retina in patients with schizophrenia. Oftalmologicheskii Zhurnal. 2020;4:38–44.

[74] Kurtulmus A, Elbay A, Parlakkaya FB, Kilicarslan T, Ozdemir MH, Kirpinar I (2020). An investigation of retinal layer thicknesses in unaffected first-degree relatives of schizophrenia patients. Schizophr Res.

[75] Lai A, Crosta C, Loftin M, Silverstein SM. Retinal structural alterations in chronic versus first episode schizophrenia spectrum disorders. Biomark Neuropsychiatr. 2020;2:2.

[76] Liu Y, Huang L, Tong Y, Chen J, Gao D, Yang F (2020). Association of retinal nerve fiber abnormalities with serum CNTF and cognitive functions in schizophrenia patients. PeerJ.

[77] Miller M, Zemon V, Nolan-Kenney R, Balcer LJ, Goff DC, Worthington M (2020). Optical coherence tomography of the retina in schizophrenia: Inter-device agreement and relations with perceptual function. Schizophr Res.

[78] Schönfeldt-Lecuona C, Kregel T, Schmidt A, Kassubek J, Dreyhaupt J, Freudenmann RW (2020). Retinal single-layer analysis with optical coherence tomography (OCT) in schizophrenia spectrum disorder. Schizophr Res.

[79] Zhuo CJ, Ji F, Xiao B, Lin XD, Chen C, Jiang DG, et al. Antipsychotic agent-induced deterioration of the visual system in first-episode untreated patients with schizophrenia maybe self-limited: Findings from a secondary small sample follow-up study based on a pilot follow-up study. Psychiatr Res. 2020;286:112906. 10.1016/j.psychres.2020.112906 32151847

[80] Zhuo CJ, Xiao B, Chen C, Jiang DG, Li GY, Ma XY, et al. Antipsychotic agents deteriorate brain and retinal function in schizophrenia patients with combined auditory and visual hallucinations: A pilot study and secondary follow-up study. Brain Behav. 2020;10:e01611. 10.1002/brb3.1611 PMC7303384 32285647

[81] Orum MH, Bulut M, Karadag AS, Dumlupinar E, Kalenderoglu A (2020). Comparison of OCT findings of schizophrenia patients using FGA, Clozapine, and SGA other than Clozapine. Arch Clin Psychiatry.

[82] Topcu-Yilmaz P, Aydin M, Ilhan BC (2019). Evaluation of retinal nerve fiber layer, macular, and choroidal thickness in schizophrenia: spectral optic coherence tomography findings. Psychiatry Clin Psychopharmacol.

[83] Delıbaş DH, Karti Ö, Erdoğan E, Şahın T, Bılgıç Ö, Erol A (2018). Decreases in retinal nerve fiber layer and ganglion cell-inner plexiform layer thickness in schizophrenia, relation to insight: A controlled study. Anadolu Psikiyatr Derg.

[84] Celik M, Kalenderoglu A, Sevgi Karadag A, Bekir Egilmez O, Han-Almis B, Şimşek A (2016). Decreases in ganglion cell layer and inner plexiform layer volumes correlate better with disease severity in schizophrenia patients than retinal nerve fiber layer thickness: Findings from spectral optic coherence tomography. Eur Psychiatry.

[85] Yilmaz U, Kucuk E, Ulgen A, Ozkose A, Demircan S, Ulusoy DM (2016). Retinal nerve fiber layer and macular thickness measurement in patients with schizophrenia. Eur J Ophthalmol.

[86] Ascaso FJ, Rodriguez-Jimenez R, Cabezón L, López-Antón R, Santabárbara J, De la Cámara C (2015). Retinal nerve fiber layer and macular thickness in patients with schizophrenia: Influence of recent illness episodes. Psychiatry Res.

[87] Lee WW, Tajunisah I, Sharmilla K, Peyman M, Subrayan V (2013). Retinal nerve fiber layer structure abnormalities in schizophrenia and its relationship to disease state: evidence from optical coherence tomography. Investig Ophthalmol Vis Sci.

[88] Ascaso FJ, Cabezon L, Quintanilla MA, Galve LG, Lopez-Anton R, Cristobal JA (2010). Retinal nerve fiber layer thickness measured by optical coherence tomography in patients with schizophrenia: A short report. Eur J Psychiatry.

[89] Bernardin F, Schwitzer T, Schwan R, Angioi-Duprez K, Ligier F, Bourion-Bedes S (2022). Altered central vision and amacrine cells dysfunction as marker of hypodopaminergic activity in treated patients with schizophrenia. Schizophr Res.

[90] Bernardin F, Schwitzer T, Angioi-Duprez K, Giersch A, Ligier F, Bourion-Bedes S (2021). Retinal ganglion cell dysfunction is correlated with disturbed visual cognition in schizophrenia patients with visual hallucinations. Psychiatry Res.

[91] Bernardin F, Schwitzer T, Angioi-Duprez K, Giersch A, Ligier F, Bourion-Bedes S (2021). Retinal dysfunctions in a patient with a clinical high risk for psychosis and severe visual disturbances: A single case report. Early Inter Psychiatry.

[92] Moghimi P, Torres Jimenez N, McLoon LK, Netoff TI, Lee MS, MacDonald A (2020). Electoretinographic evidence of retinal ganglion cell-dependent function in schizophrenia. Schizophr Res.

[93] Fradkin SI, Erickson MA, Demmin DL, Silverstein SM (2020). Absence of Excess Intra-Individual Variability in Retinal Function in People With Schizophrenia. Front Psychiatry.

[94] Demmin DL, Netser R, Roché MW, Thompson JL, Silverstein SM (2020). People with current major depression resemble healthy controls on flash Electroretinogram indices associated with impairment in people with stabilized schizophrenia. Schizophr Res.

[95] Demmin DL, Mote J, Beaudette DM, Thompson JL, Silverstein SM (2020). Retinal functioning and reward processing in schizophrenia. Schizophr Res.

[96] Bernardin F, Schwitzer T, Angioi-Duprez K, Giersch A, Jansen C, Schwan R (2020). Retinal ganglion cells dysfunctions in schizophrenia patients with or without visual hallucinations. Schizophr Res.

[97] Hébert M, Mérette C, Paccalet T, Émond C, Gagné AM, Sasseville A (2015). Light evoked potentials measured by electroretinogram may tap into the neurodevelopmental roots of schizophrenia. Schizophr Res.

[98] Warner R, Laugharne J, Peet M, Brown L, Rogers N (1999). Retinal function as a marker for cell membrane omega-3 fatty acid depletion in schizophrenia: a pilot study. Biol Psychiatry.

[99] Gerbaldo H, Thaker G, Tittel PG, Layne-Gedge J, Moran M, Demisch L (1992). Abnormal electroretinography in schizophrenic patients with a history of sun gazing. Neuropsychobiology.

[100] Marmor MF, Hock P, Schechter G, Pfefferbaum A, Berger PA, Maurice R (1988). Oscillatory potentials as a marker for dopaminergic disease. Doc Ophthalmol.

[101] Raese JD, King RJ, Barnes D (1982). Retinal oscillatory potentials in schizophrenia: Implications for the assessment of dopamine transmission in man. Psychopharmacol Bull.

[102] Adámek P, Langová V, Horáček J (2022). Early-stage visual perception impairment in schizophrenia, bottom-up and back again. Schizophrenia.

[103] Adams SA, Nasrallah HA (2018). Multiple retinal anomalies in schizophrenia. Schizophr Res.

[104] Almonte MT, Capellàn P, Yap TE, Cordeiro MF (2020). Retinal correlates of psychiatric disorders. Ther Adv Chronic Dis.

[105] Asanad S, Mohammed I, Sadun AA, Saeedi OJ (2020). OCTA in neurodegenerative optic neuropathies: emerging biomarkers at the eye-brain interface. Ther Adv Ophthalmol.

[106] Bernardin F, Schwan R, Lalanne L, Ligier F, Angioi-Duprez K, Schwitzer T (2017). The role of the retina in visual hallucinations: A review of the literature and implications for psychosis. Neuropsychologia.

[107] Cameron JR, Tatham AJ (2016). A window to beyond the orbit: the value of optical coherence tomography in non-ocular disease. Acta Ophthalmol.

[108] Diamond A, Silverstein SM, Keane BP (2022). Visual system assessment for predicting a transition to psychosis. Transl Psychiatry.

[109] Duraković D, Silić A, Peitl V, Tadić R, Lončarić K, Glavina T (2020). The Use Of Electroretinography And Optical Coherence Tomography In Patients With Schizophrenia. Acta Clin Croat.

[110] García-Portilla MP, García-Álvarez L, de la Fuente-Tomás L, Velasco-Iglesias Á, Sáiz PA, González-Blanco L (2019). Could structural changes in the retinal layers be a new biomarker of mental disorders? A systematic review and thematic synthesis. Rev Psiquiatr Salud Ment.

[111] Hosak L, Hakeem K, Raad M, Studnicka J (2015). Is microvascular abnormality a new endophenotype in schizophrenia?. Psychiatr Danub.

[112] Hosak L, Sery O, Sadykov E, Studnicka J (2018). Retinal abnormatilites as a diagnostic or prognostic marker of schizophrenia. Biomed Pap Med Fac Univ Palacky Olomouc Czech Repub.

[113] Janti SS, Tikka SK (2023). Retinal microvasculature in schizophrenia: A meta-analysis with trial sequential analysis of studies assessing vessel density using Optical Coherence Tomography Angiography. Asian J Psychiatry.

[114] Jerotic S, Ignjatovic Z, Silverstein SM, Maric NP (2020). Structural imaging of the retina in psychosis spectrum disorders: current status and perspectives. Curr Opin Psychiatry.

[115] Jurišić D, Ćavar I, Sesar A, Sesar I, Vukojević J, Ćurković M (2020). New Insights into Schizophrenia: a Look at the Eye and Related Structures. Psychiatr Danub.

[116] Karadaǧ AS, Kalenderoǧlu A (2017). Psychiatric disorders and eye: Optical coherent tomography in psychiatry aspect. Klin Psikiyatr Derg.

[117] Kennedy KG, Mio M, Goldstein BI, Brambilla P, Delvecchio G (2023). Systematic review and meta-analysis of retinal microvascular caliber in bipolar disorder, major depressive disorder, and schizophrenia. J Affect Disord.

[118] Lavoie J, Maziade M, Hébert M (2014). The brain through the retina: the flash electroretinogram as a tool to investigate psychiatric disorders. Prog Neuropsychopharmacol Biol Psychiatry.

[119] Li X, Fan F, Chen X, Li J, Ning L, Lin K (2021). Computer Vision for Brain Disorders Based Primarily on Ocular Responses. Front Neurol.

[120] Meier MH, Hill ML, Breitborde NJK (2016). Retinal imaging: A new tool for studying underlying liability to cardiovascular disease in schizophrenia. Curr Psychiatry Rev.

[121] Nguyen CTO, Hui F, Charng J, Velaedan S, van Koeverden AK, Lim JKH (2017). Retinal biomarkers provide “insight” into cortical pharmacology and disease. Pharmacol Ther.

[122] Schönfeldt-Lecuona C, Kregel T, Schmidt A, Pinkhardt EH, Lauda F, Kassubek J (2016). From Imaging the Brain to Imaging the Retina: Optical Coherence Tomography (OCT) in Schizophrenia. Schizophr Bull.

[123] Schönfeldt-Lecuona C, Schmidt A, Pinkhardt EH, Lauda F, Connemann BJ, Freudenmann RW (2014). [Optical Coherence Tomography (OCT)-a new diagnostic tool in psychiatry?]. Fortschr Neurol Psychiatr.

[124] Silverstein SM, Choi JJ, Green KM, Bowles-Johnson KE, Ramchandran RS (2022). Schizophrenia in Translation: Why the Eye?. Schizophr Bull.

[125] Silverstein SM, Demmin DL, Schallek JB, Fradkin SI. Measures of Retinal Structure and Function as Biomarkers in Neurology and Psychiatry. Biomark Neuropsychiatr. 2020;2:100018.

[126] Silverstein SM, Fradkin SI, Demmin DL (2020). Schizophrenia and the retina: Towards a 2020 perspective. Schizophr Res.

[127] Silverstein SM, Lai A (2021). The Phenomenology and Neurobiology of Visual Distortions and Hallucinations in Schizophrenia: An Update. Front Psychiatry.

[128] Silverstein SM, Rosen R (2015). Schizophrenia and the eye. Schizophr Res Cogn.

[129] Tan A, Schwitzer T, Conart JB, Angioi-Duprez K (2020). Study of retinal structure and function in patients with major depressive disorder, bipolar disorder or schizophrenia: A review of the literature. J Fr Ophtalmol.

[130] Tan A, Schwitzer T, Conart JB, Angioi-Duprez K (2020). [Retinal investigations in patients with major depressive disorder, bipolar disorder or schizophrenia: A review of the literature]. J Fr Ophtalmol.

[131] Vujosevic S, Parra MM, Hartnett ME, O’Toole L, Nuzzi A, Limoli C, et al. Optical coherence tomography as retinal imaging biomarker of neuroinflammation/neurodegeneration in systemic disorders in adults and children. Eye. 2023;37:203–19. 10.1038/s41433-022-02056-9 PMC9012155 35428871

[132] Wójciak P, Stopa M, Rybakowski F (2020). Dysfunctions of the retina and other elements of the visual system in schizophrenia. Psychiatr Pol.

[133] Youssef P, Nath S, Chaimowitz GA, Prat SS (2019). Electroretinography in psychiatry: A systematic literature review. Eur Psychiatry.

[134] Grzybowski A, Ascaso FJ, Mateo J, Cabezón L, Casas P. Other neurological disorders: Migraine, neurosarcoidosis, schizophrenia, obstructive sleep apnea-hypopnea syndrome (OSAHS). OCT in Central Nervous System Diseases: The Eye as a Window to the Brain. Springer Cham; 2016;16:167–83.

[135] Grzybowski A, Barboni P. OCT in central nervous system diseases: The eye as a window to the brain. Springer Cham; 2016;16:1–342.

[136] Grzybowski A, Barboni P. OCT and imaging in central nervous system diseases: The eye as a window to the brain. 2nd ed. Springer Cham; 2020;25:1–561.

[137] Adhan I, Bannai D, Lizano P (2020). Commentary: Can retinal imaging biomarkers inform psychosis pathophysiology?. Schizophr Res.

[138] Ahmad M, Joe P, Malaspina D, Smith RT (2019). Reply to comments on “A pilot study assessing retinal pathology in psychosis using optical coherence tomography: Choroidal and macular thickness. Psychiatry Res.

[139] Bannai D, Lizano P (2020). Identifying retinal layer endophenotypes for schizophrenia. Schizophr Res.

[140] Baytunca MB, Inci SB, Ercan ES (2019). Reply to comments on “A pilot study assessing retinal pathology in psychosis using optical coherence tomography: Choroidal and macular thickness” Reply. Psychiatry Res.

[141] Chen G, Henter ID, Manji HK (2014). Looking ahead: electroretinographic anomalies, glycogen synthase kinase-3, and biomarkers for neuropsychiatric disorders. Biol Psychiatry.

[142] Desideri LF, Barra F, Ferrero S (2019). The importance of avoiding confounding factors when measuring choroid by optical coherence tomography in psychotic patients. Psychiatry Res.

[143] Desideri LF, Vagge A, Nicolo M, Traverso CE (2020). Retinal nerve fiber layer analysis in unaffected first-degree relatives of schizophrenia patients. Schizophrenia Res.

[144] Ferro Desideri L, Vagge A, Nicolò M, Traverso CE (2020). Retinal nerve fiber layer analysis in unaffected first-degree relatives of schizophrenia patients. Schizophr Res.

[145] Fountoulakis KN (2010). Retinal response anomalies in patients with mental illness and high risk relatives. Biol Psychiatry.

[146] Kéri S (2020). The Contribution of Retinal Dysfunctions to Visual Impairments in Schizophrenia. Psychiatr Danub.

[147] Kurtulmus A, Elbay A, Ozdemir MH (2020). Response to commentary “Retinal nerve fiber layer analysis in unaffected first-degree relatives of schizophrenia patients. Schizophr Res.

[148] Malaspina D (2013). Looking schizophrenia in the eye. Am J Psychiatry.

[149] Malaspina D, Butler PD (2020). A vision for psychosis research: Commentary on “New insights into schizophrenia: A look at the eye and related structures”. Psychiatr Danubina.

[150] Schwitzer T, Schwan R, Bernardin F, Jeantet C, Angioi-Duprez K, Laprevote V (2016). Commentary: Anatomical constitution of sense organs as a marker of mental disorders. Front Behav Neurosci.

[151] Silić A, Ostojić D, Karlović D (2020). ERG and OCT as an Effective Screening and Staging Tools for Schizophrenia?. Psychiatr Danub.

[152] Silverstein SM (2020). Issues in the Aggregation of Data on Retinal Structure and Function in Schizophrenia. Schizophr Bull.

[153] Silverstein SM, Keane BP, Demmin DL, Fradkin SI (2020). Visual impairments in schizophrenia: Their significance and unrealized clinical potential. Psychiatr Danubina.

[154] Silverstein SM, Thompson JL (2020). Progress, Possibilities, and Pitfalls in Electroretinography Research in Psychiatry. Biol Psychiatry.

[155] Tan CS, Lim LW, Ting DS (2018). Assessment of choroidal and retinal thickness in psychosis. Psychiatry Res.

[156] Maziade M, Silverstein SM (2020). The place of the retina in psychiatry: Uniting neurobiological and neurodevelopmental research with clinical research in psychiatric disorders. Schizophr Res.

[157] Nguyen CTO, Acosta ML, Di Angelantonio S, Salt TE. Editorial: Seeing Beyond the Eye: The Brain Connection. Front Neurosci. 2021;15:719717. 10.3389/fnins.2021.719717 PMC8276094 34267626

[158] Schwitzer T, Leboyer M, Schwan R. A Reflection Upon the Contribution of Retinal and Cortical Electrophysiology to Time of Information Processing in Psychiatric Disorders. Front Psychiatry. 2022;13:856498. 10.3389/fpsyt.2022.856498 PMC9017967 35449563

[159] Shoham N, Cooper C (2022). Eyes, the window on psychosis?. BJPsych Open.

[160] Silverstein SM, Keane BP, Corlett PR (2021). Oculomics in Schizophrenia Research. Schizophr Bull.

[161] Silverstein SM, Thompson JL (2015). A vision science perspective on schizophrenia. Schizophrenia Res.

[162] Gagrat D, Maggiano J, Belmaker RH (1979). Effect of neuroleptic treatment in schizophrenia on the electroretinogram, electrooculogram, and color vision. Psychiatry Res.

[163] Maziade M, Jomphe V, Bureau A (2022). Little impact of cannabis use on the relation between ERG and preclinical traits in children and adolescents at genetic risk of psychosis or mood disorder. Prog Neuropsychopharmacol Biol Psychiatry.

[164] Schwitzer T, Leboyer M, Laprévote V, Schwan R (2022). Retinal electrophysiology and transition to psychiatric disorders in subjects under the influence of cannabis. Prog Neuropsychopharmacol Biol Psychiatry.

[165] Komatsu H, Onoguchi G, Jerotic S, Kanahara N, Kakuto Y, Ono T (2022). Correction: Retinal layers and associated clinical factors in schizophrenia spectrum disorders: a systematic review and meta-analysis. Mol Psychiatry.

[166] Komatsu H, Onoguchi G, Jerotic S, Kanahara N, Kakuto Y, Ono T, et al. Correction: Retinal layers and associated clinical factors in schizophrenia spectrum disorders: a systematic review and meta-analysis. Mol Psychiatry. 2023;28:2170. 10.1038/s41380-023-01983-7 36759546

[167] Adhan I, Lizano P, Bannai D, Kassety M, Miller J, Keshavan M (2020). A Pilot Study of Retinal RNFL, GCL, and Choroidal Layer in Schizophrenia Using Swept Source Optical Tomography Coherence Imaging. Biol Psychiatry.

[168] Asanad S, Addis H, Chen S, Wu JF, Kochunov P, O’Neill H, et al. Retinal Thickness and Vascular Pathology as Ocular Biomarkers for Schizophrenia: Morphometric Analysis of the Peripapillary and Macular Regions using OCT and OCTA In Vivo. Investig Ophthalmol Vis Sci. 2020;61:5105.

[169] Bannai D, Adhan I, Douglas KAA, Kasetty M, Lutz O, Keshavan M (2020). Shedding Light on Pathophysiologic Mechanisms in Schizophrenia and Bipolar Disorder Through Analysis of Retinal Structural-Vascular-Functional Relationships. Biol Psychiatry.

[170] Bannai D, Adhan I, Kasetty M, Kim LA, Hill S, Tamminga C (2021). Quantitative Retinal Microvascular Analysis in Schizophrenia With Swept Source Optical Coherence Tomography Angiography. Biol Psychiatry.

[171] Bannai D, Lutz O, Keshavan M, Miller JB, Lizano P (2019). Retinal Cytoarchitectural Abnormalities in Schizophrenia and Bipolar Disorder: A Meta-Analysis. Biol Psychiatry.

[172] Berendschot T, Appaji A, Rao N, Webers C (2020). Retinal vascular abnormalities in schizophrenia and bipolar disorder: A window to the brain. Acta Ophthalmologica.

[173] Berendschot T, Appaji A, Rao N, Webers C (2021). Retinal vascular abnormalities in schizophrenia and bipolar disorder: A window to the brain. Acta Ophthalmologica.

[174] Bernadin F, Schwitzer T, Laprevote V, Schwan R (2020). Retinal Ganglion Cells Dysfunctions In Schizophrenia Patients. Schizophrenia Bull.

[175] Bernardin F, Schwan R, Schwitzer T, Laprevote V (2018). Retinal ganglion cells dysfunction in schizophrenia patients with visual hallucinations. Eur Psychiatry.

[176] Chu EM, Kolappan M, Barnes TRE, Joyce EM, Ron MA (2009). Retinal Nerve Fibre Layer Imaging as a Possible Biomarker of Schizophrenia. Biol Psychiatry.

[177] Demmin D, Klein S, Silverstein S (2020). The Relationship Between Retinal and Cognitive Functioning in Schizophrenia. Arch Clin Neuropsychol.

[178] Demmin D, Mote J, Beaudette D, Silverstein S (2019). Electroretinographic Changes In Response To Reward In Schizophrenia. Schizophrenia Bull.

[179] Demmin D, Roche M, Menon A, Davis Q, Silverstein S (2017). Attenuated Retinal Cell Signaling And Response Gain In Schizophrenia. Schizophrenia Bull.

[180] Demmin D, Roche M, Netser R, Silverstein S (2018). Electroretinographic Indices Of Photoreceptor, Bipolar, And Ganglion Cell Functioning Differentiate People With Schizophrenia From Those With Major Depression And Healthy Controls. Schizophrenia Bull.

[181] Douglas KAA, Bannai D, Adhan I, Kasetty M, Miller JB, Keshavan M (2020). A Preliminary Study Using OCT-A to Determine Deep Layer Retinal Vascular Changes in Schizophrenia. Biol Psychiatry.

[182] Gandu S, Bannai D, Kasetty M, Hill S, Kim LA, Clementz B (2021). Inter-Device Reliability of Swept Source and Spectral Domain Optical Coherence Tomography Retinal Structure Measurements in Schizophrenia. Biol Psychiatry.

[183] Gross G, Tursini K, Koessler L, Schwan R, Schwitzer T (2021). Retinal structure and function: New biomarkers for bipolar disorders?. Bipolar Disord.

[184] Gunnarsson E, Chen V, O’Neill H, Hong E, Saeedi O. Assessing Macular Vasculature in Schizophrenia. Investig Ophthalmol Vis Sci. 2022;63:2916–F0069.

[185] Hebert M, Anne-Marie G, Dubois MA, Francis K Electroretinography anomalies in schizophrenia using a portable device. Investig Ophthalmol Vis Sci. 2019;60:5961.

[186] Jerotic S, Ristic I, Ignjatovic Z, Maric N (2020). Macular Thinning In Female Patients With Psychosis Spectrum Disorders: Preliminary Optical Coherence Tomography Findings. Schizophrenia Bull.

[187] Jerotic S, Ristic I, Ignjatovic Z, Maric N (2020). Retinal thinning in psychosis spectrum disorders: optical coherence tomography findings. Eur Neuropsychopharmacol.

[188] Kurtulmus A (2019). An investigation of retinal layer thicknesses in unaffected first degree relatives of schizophrenia patients. Eur Neuropsychopharmacol.

[189] Lizano P, Bannai D, Adhan I, Douglas KAA, Kasetty M, Keshavan M (2020). Superficial Retinal Vascular Abnormalities in Schizophrenia as Shown by Swept Source OCT-Angiography: A Preliminary Study. Biol Psychiatry.

[190] Lizano P, Karmacharya R, Keshavan M (2019). Retinal Imaging and Stem Cell Derived Brain Endothelial Cells: A Framework for Studying Blood Brain Barrier Dysfunction in Psychosis. Biol Psychiatry.

[191] Lizano P, Kasetty M, Zeng R, Wang J, Diaz D, Lutz O (2019). Examining Retinal Nerve Fiber Layer Thickness and Microvascular Abnormalities in Psychosis With Swept Source OCT and OCT-A. Biol Psychiatry.

[192] Maziade M (2018). Retinal Functions Expressed In Retinal Imaging, Contrast Processing And Electroretinography May Decrypt Early Risk Mechanisms And Pathophysiology Of Schizophrenia And Mood Disorders And Accelerate Translation To The Clinic. Schizophrenia Bull.

[193] Maziade M, Paccalet T, Rouleau N, Gilbert E, Gingras N, Jomphe V (2016). Electroretinographic and Cognitive Dysfunctions Predicts Risk of Major Mood and Psychotic Disorders in Children Born to an Affected Parent. Biol Psychiatry.

[194] Meier M, Byrne E, Martin N, Wong T, Sim XL (2018). Phenotypic And Genetic Associations Between Schizophrenia And Retinal Vessel Diameter. Schizophrenia Bull.

[195] Quintanilla MA, De La Camara C, Gutierrez L, Sanz P, Granados B, Cabezon L (2011). Reduction of peripapillary retinal nerve fiber layer thickness in schizophrenic patients. J Psychosom Res.

[196] Roy M, Strippoli MP, Hebert M, Preisig M, Marquet P (2019). Electroretinography For Major Psychiatric Disorders In Multicentric Sites. Schizophrenia Bull.

[197] Sanz P, Quintanilla MA, Granados B, Gutierrez-Galve L, De La Camara C, Cabezon L (2011). Retinal nerve fiber layer thickness as a marker of diffuse brain abnormalities in schizophrenia. Eur Neuropsychopharmacol.

[198] Sarkar S, Rajalakshmi AR, Subramanium E (2019). Retinal Nerve Fibre Layer Thickness In Patients With Schizophrenia And Their First Degree Relatives: An Optical Coherence Tomographic Study. Indian J Psychiatry.

[199] Silverstein S, Demmin D, Erickson M (2019). Retinal Anomalies In Schizophrenia And Their Clinical Significance. Schizophrenia Bull.

[200] Silverstein S, Demmin D, Erickson M, Thompson J, Paterno D, Netser R (2018). Electroretinographic Anomalies In Schizophrenia And Their Relationships With Retinal Structure, Visual Functions, Clinical Symptoms, And Medical Comorbidities. Schizophrenia Bull.

[201] Simon C, Zhang K, Chen V, Gunnarsson E, O’Neill H, Hong E, et al. Peripapillary Retinal Vessel Density as an Ocular Biomarker for Schizophrenia. Investig Ophthalmol Vis Sci. 2022;63:2934–F0087.

[202] Joseph D, Lai A, Silverstein S, Ramchandran R, Bernal EA. Computer Vision-based Classification of Schizophrenia Patients from Retinal Imagery. Electronic Imaging. 2022;34:192.

[203] Swati NV, Indiramma M. Machine learning systems for detecting schizophrenia. 2020 Fourth International Conference on I-SMAC. 2020:877–80.

[204] Joe P, Ahmad M, Riley G, Weissman J, Smith RT, Malaspina D (2018). A pilot study assessing retinal pathology in psychosis using optical coherence tomography: Choroidal and macular thickness. Psychiatry Res.

[205] Peredo R, Hébert M, Mérette C (2022). Developing a clinical decision tool based on electroretinogram to monitor the risk of severe mental illness. BMC Psychiatry.

[206] Li CY, Garg I, Bannai D, Kasetty M, Katz R, Adhan I (2022). Sex-Specific Changes in Choroid Vasculature Among Patients with Schizophrenia and Bipolar Disorder. Clin Ophthalmol.

[207] Taşdelen R, Ayık B, Kaya H, Sevimli N (2023). Investigation of the Relationship Between Cognitive Functions and Retinal Findings From Spectral Optical Coherence Tomography in Patients With Schizophrenia and Their Healthy Siblings. Psychiatry Investig.

[208] Mota M, Pêgo P, Klut C, Coutinho I, Santos C, Pires G (2015). Evaluation of Structural Changes in the Retina of Patients with Schizophrenia. Ophthalmol Res.

[209] Dinkin M (2017). Trans-synaptic Retrograde Degeneration in the Human Visual System: Slow, Silent, and Real. Curr Neurol Neurosci Rep.

[210] Onitsuka T, McCarley RW, Kuroki N, Dickey CC, Kubicki M, Demeo SS (2007). Occipital lobe gray matter volume in male patients with chronic schizophrenia: A quantitative MRI study. Schizophr Res.

[211] Pergola G, Selvaggi P, Trizio S, Bertolino A, Blasi G (2015). The role of the thalamus in schizophrenia from a neuroimaging perspective. Neurosci Biobehav Rev.

[212] Türközer HB, Lizano P, Adhan I, Ivleva EI, Lutz O, Zeng V (2022). Regional and Sex-Specific Alterations in the Visual Cortex of Individuals With Psychosis Spectrum Disorders. Biol Psychiatry.

[213] Vita A, De Peri L, Deste G, Sacchetti E (2012). Progressive loss of cortical gray matter in schizophrenia: a meta-analysis and meta-regression of longitudinal MRI studies. Transl Psychiatry.

[214] Witkovsky P (2004). Dopamine and retinal function. Doc Ophthalmol.

[215] Sekar A, Bialas AR, de Rivera H, Davis A, Hammond TR, Kamitaki N (2016). Schizophrenia risk from complex variation of complement component 4. Nature.

[216] Gao XR, Huang H, Kim H (2019). Genome-wide association analyses identify 139 loci associated with macular thickness in the UK Biobank cohort. Hum Mol Genet.

[217] Nuechterlein KH, Dawson ME (1984). A heuristic vulnerability/stress model of schizophrenic episodes. Schizophr Bull.

[218] Millan MJ, Andrieux A, Bartzokis G, Cadenhead K, Dazzan P, Fusar-Poli P (2016). Altering the course of schizophrenia: progress and perspectives. Nat Rev Drug Discov.

[219] Tranos PG, Wickremasinghe SS, Stangos NT, Topouzis F, Tsinopoulos I, Pavesio CE (2004). Macular edema. Surv Ophthalmol.

[220] Supuran CT (2019). Agents for the prevention and treatment of age-related macular degeneration and macular edema: a literature and patent review. Expert Opin Ther Pat.

[221] Laursen TM, Nordentoft M, Mortensen PB (2014). Excess early mortality in schizophrenia. Annu Rev Clin Psychol.

[222] Huhn M, Nikolakopoulou A, Schneider-Thoma J, Krause M, Samara M, Peter N (2019). Comparative efficacy and tolerability of 32 oral antipsychotics for the acute treatment of adults with multi-episode schizophrenia: a systematic review and network meta-analysis. Lancet.

[223] Pramyothin P, Khaodhiar L (2010). Metabolic syndrome with the atypical antipsychotics. Curr Opin Endocrinol Diabetes Obes.

[224] Blose BA, Lai A, Crosta C, Thompson JL, Silverstein SM. Retinal Neurodegeneration as a Potential Biomarker of Accelerated Aging in Schizophrenia Spectrum Disorders. Schizophr Bull. 2023;49:1316–24. 10.1093/schbul/sbad102 PMC10483469 37459382

[225] Domagała A, Domagała L, Kopiś-Posiej N, Harciarek M, Krukow P (2023). Differentiation of the retinal morphology aging trajectories in schizophrenia and their associations with cognitive dysfunctions. Front Psychiatry.

[226] Howes OD, Kapur S (2014). A neurobiological hypothesis for the classification of schizophrenia: type A (hyperdopaminergic) and type B (normodopaminergic). Br J Psychiatry.

[227] Mustafa A, Turgay U (2022). Optical coherence tomography angiography in patients with euthymic bipolar disorder. J Affect Disord.

[228] Ayık B, Kaya H, Tasdelen R, Sevimli N (2022). Retinal changes in bipolar disorder as an endophenotype candidate: Comparison of OCT-detected retinal changes in patients, siblings, and healthy controls. Psychiatry Res.

[229] Liu Y, Chen J, Huang L, Yan S, Gao D, Yang F (2022). Association between changes in the retina with major depressive disorder and sleep quality. J Affect Disord.

[230] Friedel EBN, Tebartz van Elst L, Schäfer M, Maier S, Runge K, Küchlin S, et al. Retinal Thinning in Adults with Autism Spectrum Disorder. J Autism Dev Disord. 2022. Online ahead of print. 10.1007/s10803-022-05882-8 PMC10907434 36550331

[231] Kaymak D, Gündoğmuş İ, Dalkıran M, Küçükevcilioğlu M, Uzun Ö (2021). Retinal Nerve Fiber Layer Thickness and Its Relationship With Executive Functions in Adult Attention Deficit Hyperactivity Disorder Patients. Psychiatry Investig.

[232] Li SL, Kam KW, Chee ASH, Zhang XJ, Chen LJ, Yip WWK (2021). The association between attention-deficit/hyperactivity disorder and retinal nerve fiber/ganglion cell layer thickness measured by optical coherence tomography: a systematic review and meta-analysis. Int Ophthalmol.

