# Supplementary material for: Retina as a potential biomarker in schizophrenia spectrum disorders: a systematic review and meta-analysis of optical coherence tomography and electroretinography
Source: Mol Psychiatry. 2023 Dec 11;29(2):464–82. doi: 10.1038/s41380-023-02340-4 (PMC11116118; doi:10.1038/s41380-023-02340-4)
Supplement: Supplementary file 1 — Supplementary information [file 41380_2023_2340_MOESM1_ESM.docx]

Supplementary information

**Retina as a potential biomarker in schizophrenia spectrum disorders: a systematic review and meta-analysis of optical coherence tomography and electroretinography.**

**Running title:** Retinal abnormalities and schizophrenia

Supplementary information includes supplementary methods, supplementary Fig. S1–S63, supplementary Tables S1–S12, PRISMA 2020 checklist.

内容

[Supplementary methods	6](#_Toc143726635)

[Fig. S1. Results of the meta-analysis of pRNFL thickness in four quadrants.	7](#_Toc143726636)

[Fig. S2. Leave-one-out analysis in pRNFL average thickness.	8](#_Toc143726637)

[Fig. S3. Leave-one-out analysis in pRNFL superior thickness.	9](#_Toc143726638)

[Fig. S4. Leave-one-out analysis in pRNFL inferior thickness.	10](#_Toc143726639)

[Fig. S5. Leave-one-out analysis in pRNFL temporal thickness.	11](#_Toc143726640)

[Fig. S6. Leave-one-out analysis in pRNFL nasal thickness.	12](#_Toc143726641)

[Fig. S7. GOSH plot showing the results of the combinatorial meta-analysis of pRNFL thickness.	13](#_Toc143726642)

[Fig. S8. GOSH plot showing the results of the combinatorial meta-analysis of pRNFL superior thickness.	14](#_Toc143726643)

[Fig. S9. GOSH plot showing the results of the combinatorial meta-analysis of pRNFL inferior thickness	15](#_Toc143726644)

[Fig. S10. GOSH plot showing the results of the combinatorial meta-analysis of pRNFL temporal thickness.	16](#_Toc143726645)

[Fig. S11. GOSH plot showing the results of the combinatorial meta-analysis of pRNFL nasal thickness.	17](#_Toc143726646)

[Fig. S12. Contour-enhanced funnel plot of pRNFL average thickness.	18](#_Toc143726647)

[Fig. S13. Contour-enhanced funnel plot of pRNFL thickness in four quadrants.	19](#_Toc143726648)

[Fig. S14. Subgroup analyses between the studies in which diabetes mellitus and hypertension were excluded (exclusion) and not (non-exclusion) in pRNFL thickness in four quadrants.	20](#_Toc143726649)

[Fig. S15. Subgroup analyses between the right and left eyes in pRNFL average thickness and thickness in three quadrants.	21](#_Toc143726650)

[Fig. S16. Results of the meta-analysis of macular volume and cup-to-disc area ratio.	22](#_Toc143726651)

[Fig. S17. Leave-one-out analysis in macular average thickness.	23](#_Toc143726652)

[Fig. S18. Leave-one-out analysis in macular volume thickness.	24](#_Toc143726653)

[Fig. S19. GOSH plot showing the results of the combinatorial meta-analysis of macular average thickness.	25](#_Toc143726654)

[Fig. S20. GOSH plot showing the results of the combinatorial meta-analysis of macular volume.	26](#_Toc143726655)

[Fig. S21. Contour-enhanced funnel plot of macular average thickness and macular volume.	27](#_Toc143726656)

[Fig. S22. Subgroup analyses between the studies in which diabetes mellitus and hypertension were excluded (exclusion) and not (non-exclusion) in macular volume.	28](#_Toc143726657)

[Fig. S23. Subgroup analyses between the right and left eyes in macular average thickness and macular volume.	29](#_Toc143726658)

[Fig. S24. Results of the meta-analysis of macular thickness in the ETDRS grid.	30](#_Toc143726659)

[Fig. S25. Leave-one-out analysis in macular central foveal thickness.	31](#_Toc143726660)

[Fig. S26. GOSH plot showing the results of the combinatorial meta-analysis of macular central foveal thickness.	32](#_Toc143726661)

[Fig. S27. Contour-enhanced funnel plot of macular thickness in the ETDRS grid.	33](#_Toc143726662)

[Fig. S28. Subgroup analyses between the studies in which diabetes mellitus and hypertension were excluded (exclusion) and not (non-exclusion) in macular thickness in the ETDRS grid.	34](#_Toc143726663)

[Fig. S29. Subgroup analyses between the right and left eyes in macular thickness in the ETDRS grid.	35](#_Toc143726664)

[Fig. S30. Leave-one-out analysis in macular superior thickness in the outer ring.	36](#_Toc143726665)

[Fig. S31. Leave-one-out analysis in macular inferior thickness in the outer ring.	37](#_Toc143726666)

[Fig. S32. Leave-one-out analysis in macular temporal thickness in the outer ring.	38](#_Toc143726667)

[Fig. S33. Leave-one-out analysis in macular nasal thickness in the outer ring.	39](#_Toc143726668)

[Fig. S34. GOSH plot showing the results of the combinatorial meta-analysis of macular superior thickness in the outer ring.	40](#_Toc143726669)

[Fig. S35. GOSH plot showing the results of the combinatorial meta-analysis of macular inferior thickness in the outer ring.	41](#_Toc143726670)

[Fig. S36. GOSH plot showing the results of the combinatorial meta-analysis of macular temporal thickness in the outer ring.	42](#_Toc143726671)

[Fig. S37. GOSH plot showing the results of the combinatorial meta-analysis of macular nasal thickness in the outer ring.	43](#_Toc143726672)

[Fig. S38. Contour-enhanced funnel plot of macular thickness in the ETDRS grid (2).	44](#_Toc143726673)

[Fig. S39. Leave-one-out analysis in macular superior thickness in the inner ring.	45](#_Toc143726674)

[Fig. S40. Leave-one-out analysis in macular inferior thickness in the inner ring.	46](#_Toc143726675)

[Fig. S41. Leave-one-out analysis in macular temporal thickness in the inner ring.	47](#_Toc143726676)

[Fig. S42. Leave-one-out analysis in macular nasal thickness in the inner ring.	48](#_Toc143726677)

[Fig. S43. GOSH plot showing the results of the combinatorial meta-analysis of macular superior thickness in the inner ring.	49](#_Toc143726678)

[Fig. S44. GOSH plot showing the results of the combinatorial meta-analysis of macular inferior thickness in the inner ring.	50](#_Toc143726679)

[Fig. S45. GOSH plot showing the results of the combinatorial meta-analysis of macular temporal thickness in the inner ring.	51](#_Toc143726680)

[Fig. S46. GOSH plot showing the results of the combinatorial meta-analysis of macular nasal thickness in the inner ring.	52](#_Toc143726681)

[Fig. S47. Contour-enhanced funnel plot of macular thickness in the ETDRS grid (3) and mGCL-IPL thickness.	53](#_Toc143726682)

[Fig. S48. Leave-one-out analysis in macular GCL-IPL.	54](#_Toc143726683)

[Fig. S49. GOSH plot showing the results of the combinatorial meta-analysis of macular GCL-IPL.	55](#_Toc143726684)

[Fig. S50. Subgroup analyses between the right and left eyes not in macular GCL-IPL, optic cup volume, and cup to disc area.	56](#_Toc143726685)

[Fig. S51. Leave-one-out analysis in optic cup volume.	57](#_Toc143726686)

[Fig. S52. Contour-enhanced funnel plot of the optic cup volume and cup-to-disc ratio.	58](#_Toc143726687)

[Fig. S53. Subgroup analyses between the studies in which diabetes mellitus and hypertension were excluded (exclusion) and not (non-exclusion) in cup to disc area.	59](#_Toc143726688)

[Fig. S54. Results of the meta-analysis of OCTA.	60](#_Toc143726689)

[Fig. S55. Contour-enhanced funnel plot of the foveal avascular zone (FAZ) and superficial foveal vessel density (VD).	61](#_Toc143726690)

[Fig. S56. Leave-one-out analysis in a-wave amplitude of photopic fERG.	62](#_Toc143726691)

[Fig. S57. Leave-one-out analysis in a-wave amplitude of scotopic fERG.	63](#_Toc143726692)

[Fig. S58. Contour-enhanced funnel plot of the a- and b-wave amplitude of the fERG.	64](#_Toc143726693)

[Fig. S59. Leave-one-out analysis in b-wave amplitude of photopic fERG.	65](#_Toc143726694)

[Fig. S60. Leave-one-out analysis in b-wave amplitude of scotopic fERG.	66](#_Toc143726695)

[Fig. S61. Results of the meta-analysis of a- and b-wave latency of fERG in photopic and scotopic conditions.	67](#_Toc143726696)

[Fig. S62. Leave-one-out analysis in a-wave latency of photopic fERG.	68](#_Toc143726697)

[Fig. S63. Contour-enhanced funnel plot of the a- and b-wave latency of the fERG.	69](#_Toc143726698)

[Table S1. OCT study characteristics in a systematic review and meta-analysis.	70](#_Toc143726699)

[Table S2. OCTA Study characteristics in a systematic review and meta-analysis.	140](#_Toc143726700)

[Table S3. ERG study characteristics in a systematic review and meta-analysis.	149](#_Toc143726701)

[Table S4. Results of the meta-analysis after removing the potential outliers identified by GOSH analysis in pRNFL.	165](#_Toc143726702)

[Table S5. Results of publication bias and publication bias-adjusted meta-analysis in OCT parameters.	167](#_Toc143726703)

[Table S6. Results of GRADE for rating the quality of a body of evidence.	170](#_Toc143726704)

[Table S7. Results of the meta-analysis after removing the potential outliers identified by GOSH analysis in the macula.	176](#_Toc143726705)

[Table S8. Results of the meta-analysis after removing the potential outliers identified by GOSH analysis in macular GCL-IPL and optic cup.	179](#_Toc143726706)

[Table S9. Results of the meta-analysis after removing the potential outliers identified by GOSH analysis in FAZ and superficial foveal vessel density.	180](#_Toc143726707)

[Table S10. Results of the meta-analysis after removing the potential outliers identified by GOSH analysis in fERG.	181](#_Toc143726708)

[Table S11. Results of publication bias-adjusted meta-analysis in fERG parameters.	182](#_Toc143726709)

[Table S12. Meta-regression analysis of the association between the explanatory variables and pooled estimates of fERG thicknesses.	184](#_Toc143726710)

[PRISMA 2020 checklist.	189](#_Toc143726711)

[Reference	194](#_Toc143726712)

# **Supplementary methods**

*Statistical analysis*

We performed a meta-analysis (MA) for retinal parameters that included three or more studies. An MA was performed to investigate the difference in optical coherence tomography (OCT) retinal parameters (peripapillary retinal nerve fiber layer [pRNFL] average thickness, pRNFL thickness in four quadrants, macular average thickness [MAT], macular volume [MV], macular thickness [MT] in Early Treatment Diabetic Retinopathy Study [ETDRS] grid,^1^ macular GCL-IPL thickness [mGCL-IPL], optic cup volume [OCV], cup-to-disk area ratio [CDR], foveal avascular zone [FAZ], and superficial foveal vessel density [VD]) among participants with schizophrenia spectrum disorders (SSDs) and healthy controls (HCs). In addition, we also evaluated the differences in amplitude and latency of flash electroretinogram (fERG) a- and b-waves under scotopic and photopic conditions and photopic negative response (PhNR) amplitude among the two groups in an MA. With regard to the OCT data, in studies in which the mean and standard deviation (sd) were described for the left and right eye, respectively, we performed an MA by calculating the mean and sd for both eyes, as previously reported.^2^ We used the mean, sd, and total number of eyes for participants with SSDs and HCs as outcomes for continuous variables and calculated the standardized mean difference (SMD) and 95% confidence intervals (CIs).

We assessed the heterogeneity among the studies included in the MA using Cochran’s Q statistic and Higgin’s inconsistency index (I^2^).^3,4^ A random-effects model was adopted to calculate the pooled SMD.^5^ If the I² and *P* values were ≥50% or <0.05, respectively, indicating high heterogeneity, we performed an influence analysis using leave-one-out analysis^6^ and created a graphical display of study heterogeneity (GOSH) analysis to identify outlier studies influencing statistical heterogeneity.^7^ In the GOSH analysis, we used the Gaussian mixture model, the DBSCAN algorithm, and the k-means algorithm to identify outliers. To assess publication bias, we plotted a contour-enhanced funnel plot, and if there were 10 or more studies included in the MA, we also performed Egger’s regression analysis.^8^ If publication bias was suspected, we recalculated the pooled SMD after adjusting for publication bias using the trim-and-fill method^9^ after removing outliers.

We performed meta-regression analysis to evaluate the effects of SSD participant age, duration of illness, percentage of male SSD participants, OCT device type (time domain [TD]–OCT, spectral domain [SD]–OCT, or swept source [SS]–OCT), psychiatric symptoms, antipsychotic dosage (chlorpromazine equivalent [mg/day]), Newcastle–Ottawa Scale (NOS), smoking (%), and body mass index (kg/m^2^) on the pooled estimates when there were five or more studies that included each explanatory variable. For psychiatric symptoms, the Positive and Negative Syndrome Scale (PANSS) total score, PANSS positive scale score, PANSS negative scale score, and PANSS general psychopathology scale score, if available, were extracted from each study.^10^

In the subgroup analysis, we divided the OCT data included in the MA into data for the right eye and data for the left eye, and we assessed whether there was a difference in retinal thickness in the pooled estimates between the right eye and the left eye. We further performed an MA only for studies that excluded diabetes and hypertension (exclusion group) and those that did not exclude diabetes and hypertension (non-exclusion group).

We used “meta,”^11^ “metafor,”^12^ and “dmetar”^13^ packages in R version 4.2.0 for the MA. Statistical significance was set at *P* < 0.05 for all analyses.

# **Fig. S1. Results of the meta-analysis of pRNFL thickness in four quadrants.**

(A) pRNFL superior thickness. (B) pRNFL inferior thickness. (C) pRNFL temporal thickness. (D) pRNFL nasal thickness.

C)

D)

A)

B)

**Fig. S2. Leave-one-out analysis in pRNFL average thickness.**

**Fig. S3. Leave-one-out analysis in pRNFL superior thickness.**

**Fig. S4. Leave-one-out analysis in pRNFL inferior thickness.**

**Fig. S5. Leave-one-out analysis in pRNFL temporal thickness.**

**Fig. S6. Leave-one-out analysis in pRNFL nasal thickness.**

**Fig. S7. GOSH plot showing the results of the combinatorial meta-analysis of pRNFL thickness.**

(A–C) Potential outliers identified by three unsupervised machine learnings (Gaussian mixture model, DBSCAN algorithm, and k-means algorithm). (D) GOSH plots with corresponding subsets containing an identified outlier colored in aqua green.

**Fig. S8. GOSH plot showing the results of the combinatorial meta-analysis of pRNFL superior thickness.**

(A–C) Potential outliers identified by three unsupervised machine learnings. (D) GOSH plots with corresponding subsets containing the identified outliers colored in aqua green.

**Fig. S9. GOSH plot showing the results of the combinatorial meta-analysis of pRNFL inferior thickness**

(A–C) Potential outliers identified by three unsupervised machine learnings. (D) GOSH plots with corresponding subsets containing the identified outliers colored in aqua green.

**Fig. S10. GOSH plot showing the results of the combinatorial meta-analysis of pRNFL temporal thickness.**

(A–C) Potential outliers identified by three unsupervised machine learnings. (D) GOSH plots with corresponding subsets containing the identified outliers colored in aqua green.

**Fig. S11. GOSH plot showing the results of the combinatorial meta-analysis of pRNFL nasal thickness.**

(A–C) Potential outliers identified by three unsupervised machine learnings. (D) GOSH plots with corresponding subsets containing the identified outliers colored in aqua green.

**Fig. S12. Contour-enhanced funnel plot of pRNFL average thickness.**

**Fig. S13. Contour-enhanced funnel plot of pRNFL thickness in four quadrants.**

(A) pRNFL superior thickness. (B) pRNFL inferior thickness. (C) pRNFL temporal thickness. (D) pRNFL nasal thickness.

C)

A)

B)

D)

**Fig. S14. Subgroup analyses between the studies in which diabetes mellitus and hypertension were excluded (exclusion) and not (non-exclusion) in pRNFL thickness in four quadrants.**

(A) pRNFL superior thickness. (B) pRNFL inferior thickness. (C) pRNFL temporal thickness. (D) pRNFL nasal thickness.

D)

A)

C)

B)

**Fig. S15. Subgroup analyses between the right and left eyes in pRNFL average thickness and thickness in three quadrants.**

(A) pRNFL average thickness. (B) pRNFL superior thickness. (C) pRNFL inferior thickness. (D) pRNFL nasal thickness.

D)

A)

B)

C)

**Fig. S16. Results of the meta-analysis of macular volume and cup-to-disc area ratio.**

(A) Macular volume. (B) Cup-to-disc area ratio.

B)

**Fig. S17. Leave-one-out analysis in macular average thickness.**

**Fig. S18. Leave-one-out analysis in macular volume thickness.**

**Fig. S19. GOSH plot showing the results of the combinatorial meta-analysis of macular average thickness.**

(A–C) Potential outliers identified by three unsupervised machine learnings. (D) GOSH plots with corresponding subsets containing the identified outliers colored in aqua green.

**Fig. S20. GOSH plot showing the results of the combinatorial meta-analysis of macular volume.**

(A–C) Potential outliers identified by three unsupervised machine learnings. (D) GOSH plots with corresponding subsets containing the identified outliers colored in aqua green.

**Fig. S21. Contour-enhanced funnel plot of macular average thickness and macular volume.**

(A) Macular average thickness. (B) Macular volume.

A)

B)

# **Fig. S22. Subgroup analyses between the studies in which diabetes mellitus and hypertension were excluded (exclusion) and not (non-exclusion) in macular volume.**

**Fig. S23. Subgroup analyses between the right and left eyes in macular average thickness and macular volume.**

(A) Macular average thickness. (B) Macular volume.

A)

B)

**Fig. S24. Results of the meta-analysis of macular thickness in the ETDRS grid.**

(A) Macular central foveal thickness. (B) Macular superior thickness in the outer ring. (C) Macular inferior thickness in the outer ring. (D) Macular temporal thickness in the outer ring. (E) Macular nasal thickness in the outer ring. (F) Macular superior thickness in the inner ring. (G) Macular inferior thickness in the inner ring. (H) Macular temporal thickness in the inner ring. (I) Macular nasal thickness in the inner ring.

G)

D)

A)

H)

I)

F)

E)

B)

C)

**Fig. S25. Leave-one-out analysis in macular central foveal thickness.**

**Fig. S26. GOSH plot showing the results of the combinatorial meta-analysis of macular central foveal thickness.**

(A–C) Potential outliers identified by three unsupervised machine learnings. (D) GOSH plots with corresponding subsets containing the identified outliers colored in aqua green.

**Fig. S27. Contour-enhanced funnel plot of macular thickness in the ETDRS grid.**

(A) Macular central foveal thickness. (B) Macular superior thickness in the outer ring. (C) Macular inferior thickness in the outer ring. (D) Macular temporal thickness in the outer ring.

C)

B)

A)

D)

**Fig. S28. Subgroup analyses between the studies in which diabetes mellitus and hypertension were excluded (exclusion) and not (non-exclusion) in macular thickness in the ETDRS grid.**

(A) Macular central foveal thickness. (B) Macular superior thickness in the outer ring. (C) Macular inferior thickness in the outer ring. (D) Macular temporal thickness in the outer ring. (E) Macular nasal thickness in the outer ring. (F) Macular superior thickness in the inner ring. (G) Macular inferior thickness in the inner ring. (H) Macular temporal thickness in the inner ring. (I) Macular nasal thickness in the inner ring.

G)

D)

A)

H)

E)

B)

I)

F)

C)

**Fig. S29. Subgroup analyses between the right and left eyes in macular thickness in the ETDRS grid.**

(A) Macular central foveal thickness. (B) Macular superior thickness in the outer ring. (C) Macular inferior thickness in the outer ring. (D) Macular temporal thickness in the outer ring. I Macular nasal thickness in the outer ring. (F) Macular superior thickness in the inner ring. (G) Macular inferior thickness in the inner ring. (H) Macular temporal thickness in the inner ring. (I) Macular nasal thickness in the inner ring.

D)

A)

G)

H)

E)

B)

I)

F)

C)

**Fig. S30. Leave-one-out analysis in macular superior thickness in the outer ring.**

**Fig. S31. Leave-one-out analysis in macular inferior thickness in the outer ring.**

**Fig. S32. Leave-one-out analysis in macular temporal thickness in the outer ring.**

**Fig. S33. Leave-one-out analysis in macular nasal thickness in the outer ring.**

**Fig. S34. GOSH plot showing the results of the combinatorial meta-analysis of macular superior thickness in the outer ring.**

(A–C) Potential outliers identified by three unsupervised machine learnings. (D) GOSH plots with corresponding subsets containing the identified outliers colored in aqua green.

**Fig. S35. GOSH plot showing the results of the combinatorial meta-analysis of macular inferior thickness in the outer ring.**

(A–C) Potential outliers identified by three unsupervised machine learnings. (D) GOSH plots with corresponding subsets containing the identified outliers colored in aqua green.

**Fig. S36. GOSH plot showing the results of the combinatorial meta-analysis of macular temporal thickness in the outer ring.**

(A–C) Potential outliers identified by three unsupervised machine learnings. (D) GOSH plots with corresponding subsets containing the identified outliers colored in aqua green.

**Fig. S37. GOSH plot showing the results of the combinatorial meta-analysis of macular nasal thickness in the outer ring.**

(A–C) Potential outliers identified by three unsupervised machine learnings. (D) GOSH plots with corresponding subsets containing the identified outliers colored in aqua green.

**Fig. S38. Contour-enhanced funnel plot of macular thickness in the ETDRS grid (2).**

(A) Macular nasal thickness in the outer ring, (B) Macular superior thickness in the inner ring. (C) Macular inferior thickness in the inner ring. (D) Macular temporal thickness in the inner ring.

A)

C)

D)

B)

**Fig. S39. Leave-one-out analysis in macular superior thickness in the inner ring.**

**Fig. S40. Leave-one-out analysis in macular inferior thickness in the inner ring.**

**Fig. S41. Leave-one-out analysis in macular temporal thickness in the inner ring.**

**Fig. S42. Leave-one-out analysis in macular nasal thickness in the inner ring.**

**Fig. S43. GOSH plot showing the results of the combinatorial meta-analysis of macular superior thickness in the inner ring.**

(A–C) Potential outliers identified by three unsupervised machine learnings. (D) GOSH plots with corresponding subsets containing the identified outliers colored in aqua green.

**Fig. S44. GOSH plot showing the results of the combinatorial meta-analysis of macular inferior thickness in the inner ring.**

(A–C) Potential outliers identified by three unsupervised machine learnings. (D) GOSH plots with corresponding subsets containing the identified outliers colored in aqua green.

**Fig. S45. GOSH plot showing the results of the combinatorial meta-analysis of macular temporal thickness in the inner ring.**

(A–C) Potential outliers identified by three unsupervised machine learnings. (D) GOSH plots with corresponding subsets containing the identified outliers colored in aqua green.

**Fig. S46. GOSH plot showing the results of the combinatorial meta-analysis of macular nasal thickness in the inner ring.**

(A–C) Potential outliers identified by three unsupervised machine learnings. (D) GOSH plots with corresponding subsets containing the identified outliers colored in aqua green.

**Fig. S47. Contour-enhanced funnel plot of macular thickness in the ETDRS grid (3) and mGCL-IPL thickness.**

(A) Macular nasal thickness in the inner ring. (B) mGCL-IPL thickness.

A)

B)

**Fig. S48. Leave-one-out analysis in macular GCL-IPL.**

**Fig. S49. GOSH plot showing the results of the combinatorial meta-analysis of macular GCL-IPL.**

(A–C) Potential outliers identified by three unsupervised machine learnings. (D) GOSH plots with corresponding subsets containing the identified outliers colored in aqua green.

**Fig. S50. Subgroup analyses between the right and left eyes not in macular GCL-IPL, optic cup volume, and cup to disc area.**

(A) Macular GCL-IPL. (B) Optic cup volume. (C) Cup to disc area.

B)

A)

C)

**Fig. S51. Leave-one-out analysis in optic cup volume.**

**Fig. S52. Contour-enhanced funnel plot of the optic cup volume and cup-to-disc ratio.**

(A) Optic cup volume. (B) Cup-to-disc ratio.

A)

B)

**Fig. S53. Subgroup analyses between the studies in which diabetes mellitus and hypertension were excluded (exclusion) and not (nonexclusion) in cup to disc area.**

**Fig. S54. Results of the meta-analysis of OCTA.**

(A) Superficial foveal VD. (B) FAZ.

A)

B)

**Fig. S55. Contour-enhanced funnel plot of the foveal avascular zone (FAZ) and superficial foveal vessel density (VD).**

(A) FAZ. (B) Superficial foveal VD.

A)

B)

**Fig. S56. Leave-one-out analysis in a-wave amplitude of photopic fERG.**

**Fig. S57. Leave-one-out analysis in a-wave amplitude of scotopic fERG.**

**Fig. S58. Contour-enhanced funnel plot of the a- and b-wave amplitude of the fERG.**

(A) The a-wave amplitude in the photopic condition. (B) The b-wave amplitude in the photopic condition. (C) The a-wave amplitude in the scotopic condition. (D) The b-wave amplitude in the scotopic condition.

C)

A)

D)

B)

**Fig. S59. Leave-one-out analysis in b-wave amplitude of photopic fERG.**

**Fig. S60. Leave-one-out analysis in b-wave amplitude of scotopic fERG.**

**Fig. S61. Results of the meta-analysis of a- and b-wave latency of fERG in photopic and scotopic conditions.**

(A) The b-wave latency of fERG in the photopic condition. (B) The a-wave latency of fERG in the scotopic condition. (C) The b-wave latency of fERG in the scotopic condition.

A)

B)

C)

**Fig. S62. Leave-one-out analysis in a-wave latency of photopic fERG.**

**Fig. S63. Contour-enhanced funnel plot of the a- and b-wave latency of the fERG.**

(A) The a-wave latency in the photopic condition. (B) The b-wave latency in the photopic condition. (C) The a-wave latency in the scotopic condition. (D) The b-wave latency in the scotopic condition.

C)

B)

D)

A)

# **Table S1. OCT study characteristics in a systematic review and meta-analysis.**

|  |  |  |  | SSD participants |  | Controls |  |  |  |
| --- | --- | --- | --- | --- | --- | --- | --- | --- | --- |
| Study | Study design | Participants | No. of eyes for OCT studies | Men (%) | Age (years, sd) | Men (%) | Age (years, sd) | Duration of illness (months, sd) | Country |
| Carriello et al. 2023^14^ | Cases and control | 35 SZ and 35 age- and sex-matched HCs | 69 eyes in SZ and 70 eyes in HCs (both eyes) | 65.7 | 36.51 (12.41) | 57.14 | 36.74 (11.94) | NA | Brazil |
| Kurtulmus et al. 2023^15^ | Cases and control | 43 chronic SZ and 40 age- and sex-matched HCs | 86 eyes in SZ and 80 eyes in HCs (both eyes) | 60 | 42.38 (10.95) | 56.7 | 38.90 (11.91) | 221.28 (118.08) | Turkey |
| Shoham et al. 2023^16^ | Cohort study | 159 SSDs (F20-29) and 97,713 HCs | 318 eyes in SSDs and 195,426 eyes in HCs (both eyes) | 56.6 | 54 (47–61) | 47.2 | 58 (50–63) | NA | UK |
| Wagner et al. 2023^17^ | Retrospective cohort study | 485 SZ and 100,931 HCs | 747 eyes in SZ and 165,400 eyes in HCs (both eyes) | 46.8 | 64.9 (12.2) | 47.2 | 65.9 (13.7) | NA | UK |
| Boudriot et al. 2022^18^ | Cases and control | 65 SSDs (47 SZ, 17 SZA, 1 BPD) and 89 HCs | 126 eyes in SSDs and 143 eyes in HCs (both eyes) | 64.6 | 39.29 (10.8) | 50 | 34.65 (11.35) | 164.28 (93.72) | Germany |
| Bozali et al. 2022^19^ | Cases and control | 57 SZ and 57 age- and sex-matched HCs | 114 eyes in SZ and in HCs (both eyes) | 59.6 | 37.2 (9.9) | 57.9 | 36.8 (9.6) | NA | Germany |
| Friedel et al. 2022^20^ | Cases and control | 25 paranoid SZ and 25 age- and sex-matched HCs | 44 eyes in SZ and 47 eyes in HCs (both eyes) | 40 | 39 (12) | 40 | 37 (12) | 108 (96) | Germany |
| Kango et al. 2022^21^ | Cases and control | 35 SZ in remission, 35 TRS, 21 first-episode antipsychotic-naïve SZ, and 36 age- and sex-matched HCs | 70 eyes in SZ in remission, and in TRS, 42 eyes in first-episode antipsychotic-naïve SZ, and 72 eyes in HCs (both eyes) | 59.3 | 30.47 (7.13) | 61.9 | 31.00 (3.68) | 72.59 (67.60) | India |
| Kaya et al. 2022^22^ | Cases and control | 46 SZ, their 46 healthy siblings, and 46 age- and sex-matched HCs | 92 eyes in SZ, in healthy siblings, and in HCs (both eyes) | 69.6 | 31.54 (8.09) | 56.5 | 30.35 (8.98) | 143.64 (82.68) | Turkey |
| Khalila et al. 2022^23^ | Cases and control | 30 SZ and 30 age-matched male HCs | 60 eyes in SZ and 60 eyes in HCs (both eyes) | 100 | 30.16 (5.6) | 100 | 29.73 (5.18) | 88.2 (54) | Egypt |
| Hanifi Kokaçya et al. 2022^24^ | Cases and control | 47 SZ and 50 age- and sex-matched HCs | 47 eyes in SZ and 50 eyes in HCs (one eye) | 76.6 | 34.74 (10.68) | 70 | 34.02 (10.30) | NA | Turkey |
| Kurt et al. 2022^25^ | Cases and control | 44 SSDs (37 SZ, 7 SZA) and 41 age- and sex-matched HCs | 88 eyes in SSDs and 82 in HCs (both eyes) | 68.2 | 47.82 (9.44) | 68.3 | 45.59 (9.92) | 297.48 (182.64) | Turkey |
| Asanad et al. 2022^26^ | Cases and control | 58 SSDs (45 SZ, 13 SZA) and 35 age- and sex-matched HCs | 116 eyes in SSDs and 70 in HCs (both eyes) | 79 | 37.2 (12.3) | 46 | 41.1 (15.2) | NA | USA |
| Alizadeh et al. 2021^27^ | Cases and control | 30 male SSDs (acute phase: N = 15, chronic phase: N = 15) and 15 age-matched male HCs | 60 eyes in SSDs and 30 in HCs (both eyes) | 100 | 39.96 (8.45) | 100 | 36.4 (9.51) | 165.84 (108.72) | Iran |
| Gandu et al. 2021^28^ | Cases and control | 30 SZ, and 22 age- and sex-matched HCs | 60 eyes in SZ and 44 eyes in HCs (both eyes) | 70 | 36.2 (12.7) | 68.1 | 37.4 (11.5) | 149.9 (148.9) | USA |
| Koman-Wierdak et al. 2021^29^ | Cases and control | 12 SZ, 8 BDs, and 15 age- and sex-matched HCs | 24 eyes in SZ, 16 eyes in BDs, and 30 eyes in HCs (both eyes) | 50 | 26.33 (5.26) | 46.67 | 26.8 (-) | 42.58 (42.01) | Poland |
| Jerotic et al. 2021^30^ | Cases and control | 42 participants with psychosis spectrum disorders (F20. x-F29) and 39 age- and sex-matched HCs | 84 eyes in psychosis spectrum disorders and 78 eyes in HCs (both eyes) | 52.38 | 32.3 (6.89) | 48.72 | 32.31 (9.52) | 72.72 (52.68) | Serbia |
| Liu et al. 2021^31^ | Cases and control | 138 SZ and 160 age- and sex-matched HCs | 276 eyes in SZ and 320 eyes in HCs (both eyes) | 52.17 | 45.09 (12.47) | 33.75 | 41.43( 9.50) | 260.88 (162.12) | China |
| Murav'eva et al. 2021^32^ | Cases and control | 14 paranoid SZ and 30 HCs | 28 eyes in SZ and 60 eyes in HCs (both eyes) | NA | NA | NA | NA | 6.7 (3.4) | Russia |
| Sarkar et al. 2021^33^ | Cases and control | 20 SZ and 20 age- and sex-matched HCs | 20 (right) eyes in SZ and 20 (right) eyes in HCs (one eye) | 70 | 28.60 (6.30) | 60 | 30.25 (9.22) | 30.21 (10.42) | India |
| Silverstein et al. 2021^34^ | Cases and control | 28 SZ or SZA and 37 HCs | 49-46 eyes in SSDs and 70 eyes in HCs (both eye) | 86 | 32.21 (11.27) | 65 | 32.22 (12.63) | NA | USA |
| Zhuo et al. 2021a^35^ | Cases and control | 97 first episode untreated SZ with combined auditory hallucinations and visual hallucinations and 30 HCs | 194 eyes in SZ and 60 eyes in HCs (both eyes) | 53.61 | 25.55 (3.77) | 50 | 25.4 (0.5) | 4.16 (2.47) | China |
| Zhuo et al. 2021b^36^ | Cases and control | 60 first episode untreated SZ who experience visual disturbances with or without auditory hallucinations and 30 HCs | 120 eyes in SZ who experience visual disturbances with or without auditory hallucinations and 60 eyes in HCs (both eyes) | 45 | 24.1 (2.82) | 50 | 24.0 (3.8) | NA | China |
| Altun et al. 2020^37^ | Cases and control | 35 SZ, 46 BDs, and 31 age- and sex-matched HCs | 70 eyes in SZ, 92 eyes in BDs, and 62 eyes in HCs (both eyes) | 75 | 44.20 (10.02) | 64 | 39.35 (4.27) | 243.6 (106.92) | Turkey |
| Budakoglu et al. 2021^38^ | Cases and control | 22 participants with SZ and 26 age- and sex-matched HCs | 22 eyes in SZ and 26 eyes in HCs (one eye) | 64 | 40.8 (7.9) | 69 | 45.2 (8.1) | 99.6 (39.6) | Turkey |
| Hosák et al. 2020^39^ | Cases and control | 39 SZ, 39 healthy first-degree relatives, and 32 age- and sex-matched HCs | 78 eyes in SZ, 78 eyes in relatives HCs, and 64 eyes in unrelated HCs (both eyes) | 69.2 | 30.5 (7.1) | 68.8 | 33.4 (9.1) | 116.4 (85.2) | Czech Republic |
| Huang et al. 2020^40^ | Cases and control | 100 first episode untreated SZ and 100 age- and sex-matched HCs | 194 eyes in SZ and 60 eyes in HCs (both eyes) | 56 | 23.0 (2.5) | 56 | 23.5 (3.0) | 2.5 (0.7) | China |
| Jerotic et al. 2020^41^ | Cases and control | 33 participants with psychosis spectrum disorders (F20.x, F25.x, and F29), and 35 age- and sex-matched HCs | 66 eyes in psychosis spectrum disorders and 70 eyes in HCs (both eyes) | 53.1 | 33.1 (6.1) | 51.4 | 32.5 (9.9) | 70.8 (46.8) | Serbia |
| Kozub et al. 2020^42^ | Cases and control | 11 paranoid SZ and one with schizotypal personality disorder, and 12 age- and sex-matched HCs | 24 eyes in paranoid SZ and schizotypal personality disorder, and 24 eyes in controls (both eyes) | 66.67 | 27.6 (NA) | 50 | 28.3 (NA) | 102 (64.8) | Russia |
| Kurtulmus et al. 2020^43^ | Cases and control | 38 chronic and stable participants with SZ, 38 unaffected first-degree relatives (siblings, parents, or children) of participants with SZ, and 38 normal controls | 76 eyes in SZ, 76 eyes in unaffected first-degree relatives, and 76 eyes in controls (both eyes) | 52.6 | 41.08 (11.26) | 52.6 | 38.47 (11.21) | 219.72 (116.88) | Turkey |
| Lai et al. 2020^44^ | Cases and control | 33 SSDs (SZ, SZA) (15 first episode and 18 chronic state) and 38 age- and sex-matched HCs | 66 eyes in SSDs and 76 eyes in HCs (both eyes) | 84.85 | 32.82 (11.87) | 63.16 | 32.0 (12.53) | 213.15 (257.97) | USA |
| Liu et al. 2020^45^ | Cases and control | 221 SZ and 149 HCs | 442 eyes in SZ and 298 eyes in HCs (both eyes) | 55.66 | 44.20 (12.02) | 63.76 | 41.59 (9.37) | 241.32 (149.4) | China |
| Miller et al. 2020^46^ | Cases and control | 12 SSDs (8 SZ, 3 SZA, 1 DD), and 12 age- and sex-matched HCs | 24 eyes in SSDs and 24 eyes in HCs (both eyes) | 66.7 | 48.25 (10.29) | 66.7 | 48.29 (10.64) | 327 (172.92) | USA |
| Orum et al. 2020^47^ | Cases and control | 26 SZ using clozapine, 34 SZ using other SGAs, 22 SZ using FGAs (44 TRS and 38 non-TRS), and 50 HCs | 164 eyes in SZ and 100 eyes in HCs (both eyes) | 78.05 | 36.76 (10.63) | 64 | 41.02 (13.69) | 173.76 (114.6) | Turkey |
| Schönfeldt-Lecuona et al. 2020^48^ | Cases and control | 26 SZ (n = 17) or SZA (n = 9) and 23 HCs | 52 eyes in SZ and SZA and 46 eyes in HCs (both eyes) | 65.38 | 37.0 (10.9) | 52.17 | 40.3 (11.6) | 122.4 (122.4) | Germany |
| Zhuo et al. 2020a^49^ | Follow-up design | 48 first-episode SZ with visual disturbance and 50 HCs | 96 eyes in first-episode SZ with visual disturbance and 100 eyes in HCs (both eyes) | 47.92 | 21.5 (1.7) | 50 | 22.0 (1.7) | NA | China |
| Zhuo et al. 2020b^50^ | Follow-up design | 97 first-episode untreated SZ with combined auditory hallucinations and visual hallucinations and 30 normal controls | 194 eyes in SZ and 60 eyes in HCs (both eyes) | 53.6 | 25.55 (3.77) | 50 | 25.4 (0.5) | 4.16 (2.47) | China |
| Topcu-Yilmaz et al. 2019^51^ | Cases and control | 59 SZ and 37 age- and sex-HCs | 59 eyes in SZ and 37 eyes in HCs (one eye) | 54.2 | 34.64 (9.49) | 40.5 | 32.08 (12.33) | 123.96 (NA) | Turkey |
| Delibas et al. 2018^52^ | Cases and control | 31 SZ with good eyesight, 32 SZ with poor eyesight, and 39 age- and sex-matched HCs | 63 eyes in SZ and 39 in HCs (one eye) | 53.97 | 40.97 (10.86) | 64.1 | 40.13 (9.03) | 175.68 (118.8) | Turkey |
| Samani et al. 2018^53^ | Cases and control | 35 SZ and 50 age- and sex-matched HCs | 70 eyes in SZ and 100 in HCs (both eyes) | 71.43 | 40.6 (12.9) | 70 | 40.6 (12.7) | 195.6 (109.2) | UK |
| Silverstein et al. 2018^54^ | Cases and control | 32 SZ and 32 HCs | 64 eyes in SZ and 64 in HCs (both eyes) | 59.38 | 40.46 (12.09) | 56.25 | 39.19 (11.03) | NA | USA |
| Celik et al. 2016^55^ | Cases and control | 40 TRS, 41 non-TRS, and 41 HCs | 80 eyes in TRS, 82 eyes in non-TRS, and 82 in HCs (both eyes) | 70.01 | 35.56 (10.14) | 68.3 | 35.49 (16.00) | 159.36 (109.8) | Turkey |
| Yılmaz et al. 2016^56^ | Cases and control | 34 SZ and 30 HCs | 68 eyes in SZ and 60 eyes in HCs (both eyes) | 55.6 | 39.85 (10.28) | 44.4 | 38.59 (9.58) | NA | Turkey |
| Ascaso et al. 2015^57^ | Cases and control | 30 SZ (10 recent illness episode participants and 20 nonrecent illness episode participants) and 30 age- and sex-matched HCs | 60 eyes in SZ and 60 eyes in HCs (both eyes) | 76.67 | 45.1 (10.4) | 73.33 | 44.5 (10.9) | 195.6 (134.4) | Spain |
| Mota et al. 2015^58^ | Cases and control | 20 SZ (10 participants with less than 5 years of illness duration and 10 participants with more than 5 years of illness duration) and 20 age- and sex-matched HCs | 40 eyes in SZ (20 eyes in participants with less than 5 years of illness duration and 20 eyes in participants with more than 5 years of illness duration) and 40 eyes in HCls (both eyes) | 85 | 32.9 (11.9) | 80 | 33.4 (11.2) | NA | Portugal |
| Lee et al. 2013^59^ | Cases and control | 30 SZ and 30 age- and sex-matched HCs | 30 (right) eyes in SZ and 30 (right) eyes in HCs (one eye) | 60 | 37.17 (10.67) | 46.7 | 35.97 (9.10) | NA | Malaysia |
| Chu et al. 2012^60^ | Cases and control | 49 SZ (n = 38) or SZA (n = 11) and 40 age- and sex-matched HCs | 98 eyes (76 eyes in SZ or 22 eyes in SZA) and 80 eyes in HCs (both eyes) | 73.47 | 29.9 (8.74) | 62.5 | 29.5 (6.12) | 52.8 (43.2) | UK |
| Ascaso et al. 2010^61^ | Cases and control | 10 SZ and 10 age- and sex-matched HCs | 20 eyes in SZ and 20 eyes in HZs (both eyes) | 80 | 39.2 (13.5) | 80 | 39.5 (13.6) | NA | Spain |

**Table S1. OCT study characteristics in a systematic review and meta-analysis (continued).**

| Study | OCT device type | OCT device model | OCT manufacturer | Diagnostic criteria | Exclusion criteria | Measurements | Main findings | NOS scores (S/C/E) |
| --- | --- | --- | --- | --- | --- | --- | --- | --- |
| Carriello et al. 2023 | SD-OCT | Spectralis HRA+OCT | Heidelberg Engineering Inc | DSM-5 | IOP ≥ 21 mm Hg; refractive error ≥ ±6 SPH D; intellectual disability; substance abuse; reported severe head trauma; uncontrolled medical condition; neurologic, rheumatologic, or autoimmune conditions; chronic infectious diseases, such as HIV/AIDS and hepatitis; nearsightedness exceeding 6 degrees; glaucoma; or any other ocular conditions that would impair the OCT examination | pRNFL average thickness, macular volume, and macular average thickness; volume and thickness of GCL, RNFL, IPL, GCL-IPL, INL, OPL, ONL, RPE, IPL, and ORL in inner ring, outer ring, and central region | Thinner macular volume and thickness in SZ participants; association between PANSS positive, general, and total scores with pRNFL | 8 (4/1/3) |
| Kurtulmus et al. 2023 | SD-OCT | Spectralis OCT | Heidelberg Engineering Inc | DSM-IV-TR | Glaucoma, uveitis, cataract, vitreous opacities, macular degeneration, or diabetic retinopathy, any previous ocular surgery or trauma, any neurologic or systemic disease known to affect the visual system, previous or concurrent autoimmune diseases, spherical equivalent of refractive error of <−6 D or >+6 D, mental retardation, illiteracy, acute psychosis, comorbid axis 1 psychiatric disorder, and substance or alcohol misuse | pRNFL average thickness and pRNFL thickness in 6 segments (superotemporal, temporal, inferotemporal, superonasal, nasal, and inferonasal); macular thickness and macular GCL, macular IPL, and choroidal thickness in three regions (temporal, subfoveal, and nasal) | Thinner right IPL in participants with SZ. In the overall sample, a negative association between IL-6, IL-1β, and TNF-α levels and left macular thickness, and IL-6 and right IPL and left choroid thickness. Negative association between thinning of the right IPL and left macula and executive functioning and attention. In participants with SZ, associations between IPL thinning with increased BMI and decreased HDL levels. In addition, association between decreased TNF-α levels and IPL thinning, especially in the left eye | 7 (2/2/3) |
| Shoham et al. 2023 | SD-OCT | NA | NA | ICD-10 | NA | Macular thickness, macular volume, and retinal pigment epithelium | No association between reduced thickness of retinal structures and subsequently measured psychotic experiences | 6 (3/1/2) |
| Wagner et al. 2023 | SD-OCT, SS-OCT | Five different Topcon devices (1000-MK2, 3DOCT-2000, 3DOCT-2000SA, FD-OCT, and Triton plus) | Topcon Inc | ICD-10 | Mental illness other than SZ, unknown sex, poor image quality, and nonmacular fixation | mGCL-IPL and mRNFL | Thinner mRNFL and mGCL-IPL in SZ participants | 6 (3/1/2) |
| Boudriot et al. 2022 | SD-OCT | Cirrus HD-OCT 5000 | Carl Zeiss Inc | DSM-5 | Age <18 years or >65 years; a concurrent clinically relevant central nervous system disorder; a history of encephalitis, meningitis, or stroke; retinal pathology; IOP ≥21 mm Hg; pregnancy; refractive error ≥±6 SPH D | pRNFL average thickness, pRNFL thickness in four quadrants, macular thickness in five segments (central foveal, superior inner, nasal inner, inferior inner, and temporal inner), mRNFL, and mGCL-IPL | Thinner parafoveal macular, mGCL-IPL, and mRNFL thickness and mean and superior pRNFL in SSD participants; negative association between duration of illness and chlorpromazine equivalent doses and parafoveal macular and mRNFL thickness | 9 (4/2/3) |
| Bozali et al. 2022 | SD-OCT | RS-3000 Advance OCT | NIDEK Inc | DSM-5 | A refractive error >±3.0 D; IOP ≥21 mm Hg; ocular pathologies including macular degeneration, any retinopathies, optic neuropathies, glaucoma, or history of ocular trauma or any ocular surgery that might interfere with the OCT measurements; known systemic or inflammatory diseases such as diabetes mellitus; media opacities intercepting ophthalmic examination; or OCT scans | pRNFL thickness in four quadrants, macular central foveal, central thickness, and ORL | Thinner ORL thickness and pRNFL thickness in the inferior and superior in SZ participants | 6 (2/2/2) |
| Fridel et al. 2022 | SD-OCT | Spectralis OCT | Heidelberg Engineering Inc | ICD-10 | Age <18 years or >65 years; body mass index <18 or >28 kg/m^2^; low-quality images (signal strengths <6/10 points); severe psychiatric issues such as substance abuse; severe ophthalmological issues such as glaucoma, age-related macular degeneration; media opacity with poor quality index in OCT printout; refractive error >4 SPH D; ophthalmic pathologies with BCVA <5/10, or ophthalmic pathologies that could affect the nerve fiber layer and macular thickness measurement; severe metabolic and endocrinologic issues such as diabetes, particularly high or low cortisol, cholestenone, or insulin concentrations; a participant with low compliance over the course of the testing period | pRNFL average thickness, pRNFL thickness in five segments (nasal, superonasal, inferonasal, superotemporal, temporal), and macular thickness, GCL, IPL, INL, OPL, and ONL in nine segments (central foveal, superior parafoveal, superior perifoveal, nasal parafoveal, nasal perifoveal, inferior parafoveal, inferior perifoveal, temporal parafoveal, and temporal perifoveal) | Thinning of the para- and perifoveal ONL in SZ participants | 7 (3/2/2) |
| Kango et al. 2022 | SS-OCT | DRI Topcon Triton | Topcon Inc | DSM-5 | Obscured clarity of media due to the presence of cataract, vitreous haze, or any other such coexistent pathology that did not allow the acquisition of good images; refractive errors ≥±3 D; any ocular pathology that could affect the retinal/choroidal thickness or vascularity such as diabetic retinopathy, macular degeneration, central serous chorioretinopathy, optic atrophy, glaucoma, and uveitis; congenital retinal pathologies; Parkinson’s disease; organic brain syndrome; intellectual disability; diabetes mellitus; hypertension; dementia; multiple sclerosis; HIV; head injury; epilepsy; and encephalopathy | mRNFL average thickness, ganglion cell thickness, outer retinal thickness, central foveal thickness, subfoveal choroidal thickness, and macular volume | Thinning subfoveal choroidal thickness in participants with psychotic illnesses compared with the controls. Significant lower subfoveal choroidal thickness in TRS compared with first-episode SZ, considering various confounders (such as age, gender, duration of treatment, smoking) | 7 (3/2/2) |
| Kaya et al. 2022 | SD-OCT | Optopol REVO OCT | Optopol Technology Sp. Z O.O. Inc | DSM-5 | Ophthalmic diseases (e.g., glaucoma, age-related macular degeneration, degenerative myopia, cataract, corneal leukoma, or vitreous hemorrhage) and diseases affecting the retina such as any type of diabetes mellitus, high blood pressure, epilepsy, or history of serious head injury, and with substance or alcohol use disorders | pRNFL average thickness, pRNFL thickness in four quadrants (superior, inferior, nasal, temporal), mGCL-IPL average thickness, mGCL-IPL thickness in two segments (superior and inferior), and macular volume | Thinner GCL+IPLs of SZ participants than controls. Macular volume was lower in SZ participants compared with siblings. A negative correlation between duration of untreated psychosis and both eyes’ pRNFL average thickness. The number of antipsychotics was positively correlated with left pRNFL temporal thickness. The number of hospitalizations and duration of illness were positively correlated with right pRNFL nasal thickness. The daily number of cigarettes was positively correlated with mean and right pRNFL thickness. Age was positively correlated with right nasal pRNFL, and BMI was positively correlated with right nasal and left superior RNFL thickness in SZ participants. | 6 (2/2/2) |
| Khalila et al. 2022 | SD-OCT | Optovue RTVue OCT | Optovue Inc | DSM-IV | Diseases that may affect OCT parameters, such as diabetes mellitus, hypertension, myopia >2.0 D, glaucoma, optic neuropathies, macular degeneration, and eyes with media opacities that may preclude OCT measurement or interfere with ocular examination, previous hypotensive crisis, ocular surgery within <3 months, or intraorbital or intracranial lesions that may interrupt the visual pathway | pRNFL average thickness, pRNFL thickness in four quadrants (superior, inferior, nasal, temporal), mGCC average thickness and mGCC in the superior and inferior segments | Thinning of the pRNFL average, superior, and nasal thickness in SZ participants compared with HCs regarding the average, superior, and nasal quadrants. Negative correlation between duration of illness and RNFL average, superior, and inferior thickness. | 7 (3/2/2) |
| Hanifi Kokaçya et al. 2022 | SD-OCT | Optovue RTVue-XR Avanti OCT | Optovue Inc | DSM-5 | Age <18 years, visual acuity <20/25 and refractive error ≥2 D, history of pregnancy, substance use, systemic disease, ocular disease, and previous ocular trauma and intraocular surgery | Average, superior hemisphere, and inferior hemisphere thickness in the whole macula, macular foveal thickness, and superior hemisphere, inferior hemisphere, average, temporal, superior, nasal, inferior thickness in parafoveal and perifoveal macula, pRNFL average thickness, and pRNFL thickness in four quadrants | Overall thinning of the macula in SZ participants compared with HCs | 6 (2/2/2) |
| Kurt et al. 2022 | SD-OCT | Cirrus HD-OCT | Carl Zeiss Inc | DSM-5 | History of uveitis, glaucoma, or invasive retinal interventions, diabetic retinopathy, or hypertensive retinopathy and refractive error >± 3.0 SPH D and/or >± 2.0 CYL D, IOP >21 mm Hg, and low-quality OCT scans (signal strength ≤6) | mRNFL and nasal, temporal, and subfoveal choroidal thickness | Thinning of all choroidal measures (nasal, subfoveal, and temporal) and mRNFL thickness in SSD participants. Negative correlation between illness duration and choroidal diameter. | 6 (2/2/2) |
| Asanad et al. 2022 | SD-OCT | Cirrus HD-OCT 5000 | Carl Zeiss Inc | DSM-5 | Macular pathologies such as age-related macular degeneration, epiretinal membrane, or macular hole; other retinopathies such as retinal vascular occlusion or retinal dystrophy; ocular diseases such as glaucoma, optic neuropathy, or uveitis; previous intraocular surgery except for uncomplicated cataract surgery; and penetrating ocular trauma | Average, superior, superior–nasal, superior–temporal, inferior, inferior–nasal, and inferior–temporal thickness of mRNFL, of GCL-IPL, and of photoreceptor complex. Macular central, inner-right, inner-superior, inner-left, inner-inferior, outer-right, outer-superior, outer-left, and outer-inferior thickness | Thinner pRNFL in SSD participants relative to HCs, most pronounced in the temporal and inferior quadrants | 8 (3/2/3) |
| Alizadeh et al. 2021 | SD-OCT | Cirrus HD-OCT 5000 | Carl Zeiss Inc | DSM-5 | Severe psychiatric issues such as substance abuse; age <18 years or >65 years, body mass index <18 or >28.8 kg/m^2^, and low-quality OCT scans (signal strength ≤6); severe ophthalmologic issues such as glaucoma, age-related macular degeneration, media opacity with poor quality index in OCT printout, and refractive error >4 SPH D; all ophthalmic pathologies with BCVA <5/10; ocular pathologies that could potentially affect the nerve fiber layer and macular thickness measurement; severe metabolic and endocrinologic issues such as diabetes, particularly high or low cortisol, cholestenone, or insulin concentrations (cutoff values: fasting blood sugar >140 mg/dL; cortisol level: range between 6 and 23 μg/dL; cholesterol >200 mg/dL; low compliance across the testing period, and presence of suicidal ideations, or worsening of general health unexpectedly | pRNFL average thickness, pRNFL thickness in four quadrants, pRNFL symmetry, neuroretinal rim area, optic disc area, average cup to disc ratio. Vertical cup to disc ratio, and cup volume. Macular central foveal thickness, and parafoveal and perifoveal thickness in four quadrants, and macular volume. | The lowest thickness in the central subfield of the right eye in participants with acute SSD compared with HCs. The largest thinning of pRNFL inferior thickness of right eye in acute SSD participants. Nonlinear associations between macula central foveal thickness and volume, and positive and negative symptoms. A longer disease duration associated with thicker pRNFL in participants with acute SSD, while a longer disease duration associated with a thinner pRNFL in participants with chronic SSD. | 8 (4/2/2) |
| Gandu et al. 2021 | SS-OCT, SD-OCT | Spectralis OCT, DRI Topcon Triton SS-OCT | Heidelberg Engineering Inc, Topcon Inc | DSM-IV TR | History of substance dependence or abuse within the past 6 months, glaucoma, macular degeneration, retinal occlusions, ocular trauma, or myopia >4.0 D, current pregnancy/breastfeeding, head injury with neurologic sequelae, intellectual disability, or neurologic disorder | Peripapillary overall retina, GCL+(GCL-IPL), GCL++(RNFL, GCL, and IPL complex) thickness, and macular overall retina, GCL+(GCL-IPL), GCL++(RNFL, GCL, and IPL complex) thickness | Positive association between GAF scores with macular layers and overall peripapillary retinal thickness. Negative association the severity of mania symptoms with peripapillary GCL-IPL thickness. Positive correlation between overall cognition with overall peripapillary retinal thickness. | 7 (3/2/2) |
| Koman-Wierdak et al. 2021 | SD-OCT | Optovue OCT | Optovue Inc | ICD-10 | Systemic diseases (diabetes mellitus, cardiovascular disorders), alcohol and other psychoactive substances dependence, any eye disease (glaucoma, macular abnormalities, high myopia, ocular trauma, retinal detachment), other diseases that may affect OCT or OCTA measurements. | pRNFL average thickness and pRNFL thickness in four quadrants. Macular whole and foveal thickness. | Lower macular thickness in the whole and the fovea in participants with SZ and BD than in the control group. | 7 (3/2//2) |
| Jerotic et al. 2021 | SD-OCT | Cirrus HD-OCT 4000 | Carl Zeiss Inc | ICD-10 | Hypertension, diabetes, or other systemic or neurologic disorders known to affect the visual system, refractive error ≥±2 SPH D; any primary ophthalmologic disorder (e.g., glaucoma, macular degeneration); previous head injury with loss of consciousness; history of substance or alcohol abuse | pRNFL average thickness, pRNFL thickness in four quadrants (superior, inferior, nasal, temporal), pRNFL symmetry, macular GCL-IPL thickness, macular central subfield thickness, macular average thickness, macular volume, optic cup volume, and cup-to-disc area ratio | Lower macular central subfield thickness and cup-to-disc area ratio in females than in males. An illness × sex interaction effect was observed for left macular thickness, indicating greater thinning in female participants with psychosis spectrum disorders. | 6 (2/2/2) |
| Liu et al. 2021 | SD-OCT | Topcon 2002 type SD-OCT | Topcon Inc | DSM-IV | Central nervous system diseases, head trauma, hypertension, diabetes mellitus, drug/alcohol abuse or addiction, glaucoma, cataract, high astigmatism (>4 SPH D and >2 CYL D), high myopia (>6.0 D), visual field defect, optic media opacities, chromatodysopia, or other significant medical illnesses (e.g., cancer, ongoing infection) | pRNFL average thickness and pRNFL thickness in the four quadrants. Macular average thickness and macular thickness in 9 segments. Disc area and cup volume. | Thinner pRNFLs in SZ participants compared with controls. Negative association between pRNFL thickness with the Stroop color and word test scores. Positive correlation of total and inferior pRNFL thicknesses of right eyes with vascular endothelial growth factor level. Negative association between total RNFL thicknesses with duration of illness. | 8 (4/2/2) |
| Murav'eva et al. 2021 | SD-OCT | Topcon 3D OCT 2000 | Topcon Inc | NA | Extreme myopia, any macular pathology, or any neuro-opticopathy | Macular average thickness, macular thickness in nine segments, macular volume, and foveal length | Increased total macular volume, average retinal thickness, retinal thickness in the superior inner macula, in the superior outer macula, and in the temporal outer macula in participants with SZ when compared with the control | 5 (2/1/2) |
| Sarkar et al. 2021 | SD-OCT | Spectral OCT/SLO version 4.0, combination imaging system | OTI-OPKO Inc | ICD-10 | Associated systemic comorbidities, diabetes, hypertension, and coronary artery disease, and ocular diseases that could affect the macula and optic nerve, such as glaucoma, optic atrophy, optic neuropathies, retinal vascular diseases, macular dystrophies, and degenerations. A history of ocular trauma, previous intraocular surgeries, refractive error >2 SPH D, and significant media opacities precluding good imaging by OCT | pRNFL average thickness, pRNFL thickness in four quadrants, and macular thickness in nine segments (central foveal, superior inner, superior outer, nasal inner, nasal outer, inferior inner, inferior outer, temporal inner, and temporal outer) | Reduction of macular central foveal, temporal inner, nasal inner, temporal outer, inferior outer, and nasal outer thickness in participants with SZ compared with HCs | 7 (3/2/2) |
| Silverstein et al. 2021 | SD-OCT | Cirrus HD-OCT 5000 | Carl Zeiss Inc | NA | A history of eye injury or disease (eg, cataracts, macular degeneration, diabetic retinopathy, glaucoma); neurological, intellectual, mood, or developmental disorders; a history of head injury with loss of consciousness of more than 10 minutes; electroconvulsive therapy within the last 8 weeks; amblyopia (lazy eye) or a squint; and diabetes or high blood pressure. | Superficial perfusion density and vessel density in macular region and FAZ. Macula CSF thickness, macular volume, and pRNFL thickness. | For the entire samples, a positive association between perfusion density or vessel density and macula CSF thickness and macula cube volume for the left eye. The weak association between perfusion density and macular CSF thickness for the right eye. For the SZ group alone, a positive correlation between perfusion density or vessel density and macula CSF thickness for the left eye. For the entire samples, an association between perfusion density or vessel density and pRNFL thickness for the left eye. For the SZ group, the association between perfusion density or vessel density and pRNFL thickness for the left eye. | 6 (2/2/2) |
| Zhuo et al. 2021a | SD-OCT | Cirrus HD-OCT 4000 | Carl Zeiss Inc | DSM-IV | Moderate to severe physical disease (e.g., respiratory, cardiovascular, endocrine, neurologic, liver, or kidney disease), currently receiving electroconvulsive therapy, a history of loss of consciousness >5 minutes for any cause, left-handedness, as determined by the AHPQ, ophthalmic disease, high myopia, any MRI contraindication, including claustrophobia, and IQ <80 | Total retinal thickness and RNFL thickness in the foveal region, nasal parafoveal region, and temporal parafoveal region | Thinning of total retinal thickness in the macular foveal, nasal parafoveal, and temporal parafoveal regions in first-episode untreated participants with SZ with combined auditory hallucinations and visual hallucinations compared with HCs | 8 (4/2/2) |
| Zhuo et al. 2021b | SD-OCT | Cirrus HD-OCT 4000 | Carl Zeiss Inc | DSM-IV | Normal controls with any neuropsychiatric disorder or first-degree relative with any neuropsychiatric disorder. Moderate or severe liver disease, kidney disease, or comorbidity of the respiratory, cardiovascular, endocrine, or nervous system, currently being treated with electroconvulsive therapy, history of loss of consciousness for more than 5 minutes, left-handedness, ophthalmic disease or severe myopia, and any MRI contraindication | Total macular thickness and RNFL thickness in three macular regions (foveal region, nasal parafoveal region, and temporal parafoveal region) | Most first-episode untreated SZ participants with visual disturbances (85.0%; 51/60) had primary visual cortex–retina co-impairments. Significant retinal thickness reductions in the macular foveal, nasal parafoveal, and temporal parafoveal regions, in first-episode participants with untreated SZ with visual disturbances with auditory hallucinations compared with participants with first-episode untreated SZ with visual disturbances without auditory hallucinations and HCs | 8 (4/2/2) |
| Altun et al. 2020 | SD-OCT | Optovue RTVue Premier OCT | Optovue Inc | DSM-5 | An additional antipsychotic to risperidone and clozapine at a dose exceeding the equivalent of 300 mg chlorpromazine. Mental retardation; any additional medical, psychiatric, or neurologic disease; any ocular pathology (glaucoma, history of refractive error >±2 SPH/CYL D, ocular trauma, ocular surgery in the past 3 months); any condition such as hypertension or diabetes mellitus that could lead to macular degeneration or optic neuropathy; intracranial or intraocular lesions; history of brain surgery | pRNFL thickness in seven segments (nasal, superonasal, inferonasal, superotemporal, temporal, inferotemporal, and global). Macular thickness for the right and left eyes in nine segments (central foveal, superior inner, superior outer, nasal inner, nasal outer, inferior inner, inferior outer, temporal inner, and temporal outer) | Moderately positive correlation between antipsychotic doses (APDs) with right temporal and left temporal RNFL thickness. Moderately negative correlation between APDs with left nasal RNFL thickness. In addition, moderately negative correlation between risperidone doses with left temporal, left nasal, and left inferonasal RNFL thickness. | 7 (3/2/2) |
| Budakoglu et al. 2021 | SD-OCT | Optovue RTVue XR Avanti OCT | Optovue Inc | DSM IV-TR | Pathology in the anterior segment and/or posterior segment examination, corrected IOP >21 mm Hg, refractive error ≥±1 SPH/CYL D. Any systemic disease that will affect retinal vascular structures, such as diabetes mellitus or essential hypertension, media opacity that will affect imaging, participants who underwent ocular surgery, and those under 18 years of age. | Optic disc vascular density, cup- to-disc area ratio, pRNFL average thickness, and pRNFL thickness in four quadrants | Lower pRNFL thickness and the mean peripapillary vascular density values in the temporal quadrant in participants with SZ than in the control group | 6 (2/2/2) |
| Hosák et al. 2020 | SD-OCT | Cirrus HD-OCT | Carl Zeiss Inc | DSM-5 | Hypertension, heart disease or stroke, and disorders potentially influencing retinal microvasculature, such as age-related macular degeneration or major deficits in visual acuity | pRNFL average thickness, pRNFL thickness in four quadrants, macular GCL-IPL average thickness, macular GCL-IPL thickness in six segments, macular central subfield thickness, macular volume, and diameters of blood vessels | Thinning of pRNFL nasal and GCL-IPL thickness in the right eye in participants with SZ as compared with those in the control group | 6 (2/2/2) |
| Huang et al. 2020 | SD-OCT | Cirrus 4000 HD-OCT | Carl Zeiss Inc | DSM-IV | Substance abuse, diabetes or glycosylated hemoglobin level outside normal range, ophthalmic disease or concurrent OCT-related condition, concurrent systemic (e.g., respiratory, cardiovascular, endocrine, neurologic, liver, or kidney disease) or chronic disease, a history of severe head trauma, current electroconvulsive therapy, high-grade myopia (≥600 D), and IQ <80 | Total macular thickness in three macular regions (the foveal region, nasal parafoveal region, and temporal parafoveal region) | Positive association between the Bonn Scale for the Assessment of Basic Symptoms scores–based visual perception subjective experience scale with total retinal reduction in the macular foveal region | 7 (3/2/2) |
| Jerotic et al. 2020 | SD-OCT | Cirrus HD-OCT 4000 | Carl Zeiss Inc | ICD-10 | Hypertension, diabetes, or other systemic and neurologic disorders known to affect the visual system, refractive error ≥±2 SPH D, any primary ophthalmologic disorder (e.g., glaucoma, macular degeneration), previous head injury with loss of consciousness, history of substance or alcohol abuse, and mental retardation | pRNFL average thickness, pRNFL thickness in four quadrants, pRNFL symmetry, macular GCL-IPL thickness, macular central subfield thickness, macula average thickness, macular volume, optic cup volume, and cup-to-disc area ratio | Thinning of GCL-IPL thickness and macular volume of both eyes in participants with psychosis spectrum disorders. The thinning of macular central subfield thickness of the right eye and average macular thickness of the left eye in participants with psychosis spectrum disorders. Negative association between the duration of illness with RNFL average thickness in the right eye and the pRNFL superior thickness in both eyes. Negative association between the mean dose of antipsychotic medication with pRNFL inferior thickness of the left eye in participants with psychosis spectrum disorders. | 6 (2/2/2) |
| Kozub et al. 2020 | SD-OCT | Topcon 3D OCT 2000 | Topcon Inc | NA | High myopia with secondary changes in the fundus, any macular disorder, or optic neuropathy | Foveal diameter, optic disc area, cup-to-disc area ratio, neuroretinal rim area, pRNFL average thickness, macular average thickness, four quadrant thickness, foveal thickness, and macular volume | Increase in the macular volume, superior inner retinal thickness, and nasal inner retinal thickness for both eyes in participants with SZ compared with HCs | 5 (2/1/2) |
| Kurtulmus et al. 2020 | SD-OCT | Spectralis OCT | Heidelberg Engineering Inc | DSM-IV | Previous or concurrent systemic disease that may involve the eyes (including diabetes, hyperlipidemia, hypertension, and cardiovascular diseases), any preexisting retinal or ocular pathology including glaucoma, cataract, macular degeneration, diabetic retinopathy and uveitis, history of ocular surgery/trauma, any neurologic condition known to affect the visual pathway, refractive error <−4 SPH D or >+3 SPH D, and substance or alcohol misuse within the past year according to the history taken and medical health records | pRNFL average thickness and pRNFL thickness in six segments (superotemporal, temporal, inferotemporal, superonasal, nasal, and inferonasal). Macular central foveal thickness, macular GCL, and macular IPL. | Thinning of IPL thickness in participants compared with HCs | 6 (2/2/2) |
| Lai et al. 2020 | SD-OCT | Cirrus HD-OCT 5000 | Carl Zeiss Inc | DSM-5 | History of eye injury or disease (e.g., cataracts, macular degeneration, diabetic retinopathy, glaucoma); neurologic, intellectual, mood, or developmental disorders; a history of head injury with loss of consciousness of more than 10 minutes; electroconvulsive therapy within the past 8 weeks; amblyopia (lazy eye) or a squint; diabetes; or high blood pressure | pRNFL average thickness, cup-to-disc area ratio, macular central foveal thickness, macular GCL-IPL, and macular volume | Reduction of GCL-IPL thickness, overall macula thickness, and macular volume in participants with chronic SZ and schizoaffective disorder not in participants with first-episode psychosis | 5 (2/1/2) |
| Liu et al. 2020 | SD-OCT | Topcon 2002 type SD-OCT | Topcon Inc | DSM-IV | Acute and chronic medical conditions, including inflammation, pregnancy, hypertension, head trauma, diabetes mellitus, drug and alcohol abuse or addiction, glaucoma, cataracts, high astigmatism (refractive error >4 SPH D and >2 CYL D), and optic media opacities | pRNFL average thickness and pRNFL thickness in the four quadrants. Macular average thickness and macular thickness in nine segments. Disc area and cup volume. | Thinning of pRNFL in SZ participants compared with HCs. Negative association between pRNFL thickness with duration of illness and serum ciliary neurotrophic factor in SZ participants. Positive association between immediate memory and visuospatial functions and pRNFL thickness. | 8 (4/2/2) |
| Miller et al. 2020 | SD-OCT | Spectralis OCT, Cirrus 4000 OCT | Heidelberg Engineering Inc, Carl Zeiss Inc | DSM-5 | History of neurologic disorders or diseases of the eye (e.g., cataracts, glaucoma) | pRNFL average thickness, macular volume, and mGCL-IPL thickness | Only in HCs, negative associations between King–Devick performance and pRNFL thickness and macular volume and positive association between low-contrast visual acuity and mGCL-IPL thickness | 6 (2/2/2) |
| Orum et al. 2020 | SD-OCT | SpectralisTM OCT | Heidelberg Engineering Inc | DSM-5 | Comorbid first-axis diagnosis, hypertension, diabetes mellitus, severe neurologic disorder, drug use other than antipsychotic drugs, immunologic and systemic diseases, glaucoma, retinal diseases, or refractive error ≥1 SPH D | pRNFL thickness in seven segments (temporo-superior, temporo-inferior, temporal, nasal superior, nasal inferior, nasal, and global), choroid thicknesses, and pGCL and pIPL volumes | Thinning of pRNFL, pGCL, and pIPL volume in antipsychotic medicated SZ participants compared with HCs. Significant difference between the first-generation antipsychotic group, the second-generation antipsychotic group, and the clozapine group; between the control group and first-generation antipsychotic group in terms of choroidal thickness. Thinning of pGCL, pIPL, and choroidal thickness in TRS compared with non-TRS. | 6 (3/1/2) |
| Schönfeldt-Lecuona et al. 2020 | SD-OCT | Spectralis OCD | Heidelberg Engineering Inc | ICD-10 | Ophthalmologic disorders or systemic diseases that could affect the optic nerve or the retina (such as diabetes, glaucoma, or refractory arterial hypertension), neurologic diseases affecting the brain, clinically relevant cognitive impairment revealed by an MMSE score of <26, current substance abuse, high-dose steroid therapy, recent eye surgery, autoimmune diseases affecting the eye, IOP >21 mm Hg or, refractive error >6 diopter. | RNFL thickness and volume, GCL + IPL, INL, OPL, and ONL in the macular region in addition to pRNFL | Reduced macular volume, macular thickness, mRNFL, and INL in participants with SSDs compared with the HCs. Negative association between the duration of illness and the total volume of the mRNFL. | 8 (4/2/2) |
| Zhuo et al. 2020a | SD-OCT | Cirrus HD-OCT 4000 | Carl Zeiss Inc | DSM-IV-TR | Participants treated with antipsychotic therapeutics within 3 weeks prior to enrollment, contraindications for MRI, and with ophthalmic diseases, diseases affecting the retina, evidence of substance abuse, prevalence of any disease that could cause visual disturbances (e.g., hyperglycemia, hyperlipidemia, or muscae volitantes), other systemic diseases (e.g., respiratory, cardiovascular, endocrine, neurologic, liver, or kidney disease) chronic diseases, history of loss of consciousness for more than 5 minutes for any reason or previous head trauma, and IQ <80 | Total macular thickness and RNFL thickness in three macular regions (foveal region, nasal parafoveal region, and temporal parafoveal region) | Thinning of total retinal thickness at baseline in the foveal, temporal parafoveal, and nasal parafoveal regions in participants with first-episode SZ with visual disturbance compared with HCs. Reduction in total retinal thickness in the three regions after 3 years of antipsychotic treatment in the participants. Positive association between gray matter reduction and retinal thinning in the three regions in the participants. Negative association between total retinal thickness reduction in the three regions and the increase in the Bonn Scale for Assessment of Basic Symptoms scores. | 8 (4/2/2) |
| Zhuo et al. 2020_2 | SD-OCT | Cirrus HD-OCT 4000 | Carl Zeiss Inc | DSM-IV | Moderate to severe physical disease (e.g., respiratory, cardiovascular, endocrine, neurologic, liver, or kidney disease), current reception of electroconvulsive therapy, history of loss of consciousness >5 minutes for any cause, left-handedness, as determined by the AHPQ, ophthalmic disease, high myopia, any MRI contraindication, including claustrophobia, and IQ <80 | Total retinal thickness and RNFL thickness in the foveal region, nasal parafoveal region, temporal and parafoveal region | Thinning of total retinal thickness in the macular foveal, nasal parafoveal, and temporal parafoveal regions in first-episode untreated SZ participants with combined auditory hallucinations and visual hallucinations compared with HCs | 8 (4/2/2) |
| Topcu-Yilmaz et al. 2019 | SD-OCT | Spectralis OCT | Heidelberg Engineering Inc | DSM-IV | A history of an ophthalmologic, neurologic, or systemic conditions that could affect the RNFL or macula (such as amblyopia, glaucoma, age-related macular degeneration, diabetes mellitus, hypertension, optic neuropathy, ocular trauma, ocular surgery, IOP ≥21 mm Hg, etc.), refractive errors ≥± 2.0 SPH D, and media opacities that preclude OCT examination | pRNFL average thickness and pRNFL thickness in six segments (temporo-superior, temporo-inferior, temporal, nasal superior, nasal inferior, nasal). Macular thickness in nine segments. Choroidal thickness at the fovea and at positions 500, 1,000, and 1,500 µ nasal and temporal to the fovea. | Thinning of macular thickness in the superior inner, nasal inner, inferior inner, temporal inner, and temporal outer segments in SZ participants compared with HCs. Negative correlation between duration of illness and choroidal thickness. | 6 (2/2/2) |
| Delibas et al. 2018 | SD-OCT | Cirrus HD-OCT 4000 | Carl Zeiss Inc | DSM-IV | Medical conditions that may affect the CNS or retina (such as diabetes mellitus), alcohol and/or substance abuse in the past 6 months, previous ocular surgery or trauma, history of ocular diseases (such as mature cataract, corneal opacity, uveitis, vitreous hemorrhage, glaucoma, retinal vascular disease) | pRNFL thickness in four quadrants and macular GCL-IPL thickness in six segments (superior temporal, superior, superior nasal, inferior temporal, inferior, and inferior nasal) | Thinning of four quadrants of RNFL, superior temporal, superior, and inferior quadrants of GCL-IPL in participants with SZ compared with in HCs. Thinning of inferior, inferior nasal quadrants of GCL-IPL, and all quadrants of RNFL except the nasal quadrant in participants with poor eyesight than in participants with good eyesight and HCs. | 7 (3/2/2) |
| Samani et al. 2018 | SD-OCT | Leica Envisu hand-held SD-OCT | Bioptigen Inc | ICD-10 | Participants with any previously diagnosed ophthalmologic diseases, diabetes mellitus, ocular surgery/trauma, refractive error ≥±6 SPH D, and substance dependency | Average individual and combined layer thickness measured from three macular regions relative to the foveal center (foveal region, nasal parafoveal region, and temporal parafoveal region). Individual layer thickness includes RNFL, GCL, IPL, INL, OPL, ONL, ISL, OSL, cone outer segment tips, and retinal pigmented epithelium. Combined layer thickness includes total retina, photoreceptor complex, processing complex, and ganglion cell complex. | Reduced total retinal and photoreceptor complex thickness in three macular regions (foveal, temporal parafoveal, and nasal parafoveal region) in participants with SZ. Thinning of the ONL and ISL in three macular regions, as well as temporal parafoveal GCL thickness. Positive correlation between low spatial frequency contrast sensitivity correlated with temporal parafoveal ganglion cell complex thinning. Negative correlation between symptom severity with foveal photoreceptor complex thickness and ONL thickness. | 7 (3/2/2) |
| Silverstein et al. 2018 | SD-OCT | Cirrus HD-OCT 4000 | Carl Zeiss Inc | DSM-IV | Retinal or other eye injury or disease (e.g., cataracts, macular degeneration, diabetic retinopathy, glaucoma), neurologic disorders, intellectual and developmental disorders (e.g., autism), active substance abuse disorders for at least 6 months, or a history of head injury with loss of consciousness of >10 minutes | The pRNFL average thickness, pRNFL thickness in four quadrants, pRNFL symmetry, macula central subfield thickness, macula average thickness, and macular thickness in the four outer quadrants, macula volume, mGCL-IPL thickness, optic cup volume, and cup-to-disc area ratio. | No difference in pRNFL, macula, or GCL-IPL thickness among participants with SZ and HCs. Thinning of pRNFL, macular thickness, or mGCL-IPL thickness related to the presence of diabetes or hypertension. Enlarged cup volume and cup to-disc ratio in both eyes, which were unrelated to medical comorbidity, but were related to increased cognitive symptoms in participants. | 7 (3/2/2) |
| Celik et al. 2016 | SD-OCT | Spectralis OCT | Heidelberg Engineering Inc | DSM-IV | Degenerative neurologic, immunologic, systemic illnesses, primary ophthalmologic diseases (glaucoma, retinal diseases, etc.), refractive error >±1 SPH/CYL D | The pRNFL thickness in seven segments (temporo-superior, temporo-inferior, temporal, nasal superior, nasal inferior, nasal, and global) and choroidal thickness, GCL, and IPL volumes | Reduced global RNFL thickness and GCL and IPL volumes in participants with SZ compared with HCs. Reduced choroid thickness, GCL and IPL volumes in TRS participants compared with non-TRS participants. Negative correlations between the GCL and IPL volumes and PANSS total score, CGI, and number of hospitalizations in participants with SZ. Negative correlations between choroid thickness, and duration of illness, and number of hospitalizations in participants with SZ. | 8 (4/2/2) |
| Yılmaz et al. 2016 | SD-OCT | Cirrus 4000 HD-OCT | Carl Zeiss Inc | NA | Retinal, macular, optic disc disease, prominent cataract, and corneal pathology impair measurement quality, retinal laser, ocular surgery, systemic diseases such as systematic hypertension or diabetes mellitus that could affect the macular or retinal thickness, or smokers | Average pRNFL and pRNFL in four quadrants and macular layer thickness in eight segments (superior inner, superior outer, nasal inner, nasal outer, inferior inner, inferior outer, temporal inner, and temporal outer). | Thinning of pRNFL average and nasal thickness and macular nasal outer and inferior outer thickness in participants with SZ as compared with HCs. | 6 (2/2/2) |
| Ascaso et al. 2015 | TD-OCT | Stratus OCT | Carl Zeiss Inc | DSM-IV | Previous or concurrent systemic disease that may affect the eyes; history of ophthalmologic or neurologic disease known to affect the visual pathway (e.g., glaucoma, age-related macular degeneration, diabetic retinopathy, degenerative myopia); media opacification such as cataract, corneal leukoma, or vitreous hemorrhage that impede ocular and OCT examination; refractive error ≥± 2 SPH D; history of head injury with loss of consciousness; and drug or alcohol dependence | The pRNFL thickness in four quadrants (temporal, superior, nasal, and inferior) and overall pRNFL thickness. Total average macular thickness, macular thicknesses in the inner and outer rings, and foveal thickness, and macular volume. | Reduced pRNFL overall and nasal, superior, and inferior thickness; macular inner ring thickness; and macular volume in participants with SZ compared with HCs. Reduced RNFL overall, superior nasal thickness, macular volume, and macular inner ring thickness only in participant with SZ with nonrecent illness episode compared with HCs. | 6 (2/2/2) |
| Mota et al. 2015 | SD-OCT | Spectralis OCT | Heidelberg Engineering Inc | ICD-10 | Degenerative or neurologic diseases; general systemic diseases that could affect the eye, such as autoimmune or infectious diseases; or history of traumatic brain injury associated with loss of consciousness. Episodes of acute decompensation, current history of prominent addictive behavior (alcohol or other illegal drugs), history of macrostructural lesions of the central nervous system, and other relevant comorbid psychiatric disorders, particularly mental retardation, or dementia. Refractive error ≥−6.00 D, BCVA <8/10, IOP ≥22 mm Hg, opacification of the transparent media of the eye; change in excavation/upper disc >0.4, and pathology of the retinal posterior pole, such as diabetic retinopathy, hypertension, or another. | pRNFL average thickness and pRNFL thickness in six segments (temporo-superior, temporo-inferior, temporal, nasal superior, nasal inferior, and nasal). Overall macular thickness and volume, macular thickness and volume in outer and inner ring subdivided into four quadrants, and in the fovea. | Reduced macular thickness and volume in nine segments in addition to macular overall thickness and volume in participants with SZ compared with HCs. Negative correlation between duration of illness and pRNFL average thickness in participants with SZ. Reduced thickness of the temporal outer ring of the macula in the group with more than 5 years compared with SZ participants for less than 5 years and SZ participants with more than 5 years. | 6 (2/2/2) |
| Lee et al. 2013 | SD-OCT | Cirrus HD-OCT 4000 | Carl Zeiss Inc | DSM IV-TR | Conditions that might affect the retinal nerve fiber structures including any ocular pathology, such as macular degeneration or optic neuropathies, glaucoma, history of ocular trauma, ocular diseases, recent ocular surgery within the past 3 months, previously known history of hypotensive crisis, or with any history of intracranial or intraorbital space-occupying lesions that can affect the visual pathway. Diabetes mellitus, refractive error >−2.0 SPH D, and significant media opacities precluding ocular examination or OCT measurement. | pRNFL average and pRNFL thickness in four quadrants. The macular volume, average and central thickness, and macular thickness in eight segments (superior inner, superior outer, nasal inner, nasal outer, inferior inner, inferior outer, temporal inner, and temporal outer). | Reduction in pRNFL average thickness, pRNFL thickness in four quadrants, macular average thickness, macular thickness in nine segments, and macular volume in participants with SZ compared with HCs. Reduction in pRNFL thickness, macular average thickness, and macular volume in chronic and long-term chronic SZ participants compared with HCs. Negative correlation between pRNFL thickness, macular thickness, and macular volume with the duration of illness in SZ participants. | 8 (4/2/2) |
| May-Yin Chu et al. 2012 | TD-OCT | Stratus OCT | Carl Zeiss Inc | DIP-DM | Concurrent or previous systemic disease (e.g., diabetes or autoimmune disease) that could involve the eyes, a history of neurologic or ophthalmologic disease known to affect the visual pathway (e.g., glaucoma), refractive error >−6 SPH D, previous head injury with loss of consciousness, and drug or alcohol dependence | pRNFL average thickness, pRNFL thickness in four quadrants, and macular volume | Thinning of pRNFL in the right nasal quadrant in SZA participants than in SZ participants. Negative association between positive symptom severity and macular volume. | 6 (2/2/2) |
| Ascaso et al. 2010 | TD-OCT | Stratus OCT | Carl Zeiss Inc | DSM-IV | Ocular posterior pole pathology such as macular degeneration or diabetic retinopathy, glaucoma suspect, or media opacification such as cataract or vitreous hemorrhage | pRNFL average thickness and pRNFL thickness in four quadrants. Macular thickness in the inner ring and the outer ring, foveal thickness, and macular volume. Cup-to-disc area ratio, horizontal cup-to-disc area ratio, and vertical cup-to-disc area ratio. | Reduced overall pRNFL thickness and pRNFL thickness in the nasal quadrant in participants with SZ compared with HCs. | 6 (2/2/2) |

Abbreviations: AHs, auditory hallucinations; AHPQ, Annett Hand Preference Questionnaire; APD, ; BCVA, best-corrected visual acuity; BD, bipolar disorder; BMI, body mass index; CGI, ; CNS, central nervous system; CSF, central subfiled; CYL, optical cylindrical equivalent; D, diopter; DIP-DM, Diagnostic Interview for Psychosis–Diagnostic Module; DSM-IV, *Diagnostic and Statistical Manual of Mental Disorders–Fourth Edition*; DSM-IV-TR, *Diagnostic and Statistical Manual of Mental Disorders, Fourth Edition, Text Revision*; DSM-5, *Diagnostic and Statistical Manual of Mental Disorders,* *Fifth Edition*; FGAs, first-generation antipsychotics; GCL-IPL, ganglion cell layer with inner plexiform layer; HC, healthy control; ICD-10, *International Statistical Classification of Diseases and Related Health Problems*, 10th Revision; IQ, intelligence quotient; INL, inner nuclear layer; IOP, intraocular pressure; ISL, inner segment layer; logMAR, logarithm of the minimum angle of resolution; mGCC, macular ganglion cell complex; mGCL-IPL, macular GCL-IPL; MRI, magnetic resonance imaging; MMSE, Mini-Mental State Examination; mRNFL, macular RNFL; NOS, Newcastle–Ottawa Scale; OCT, optical coherence tomography; ONL, outer nuclear layer; OPL, outer plexiform layer; ORL, outer retinal layer thickness; OSL, outer segment layer; PANSS, Positive and Negative Syndrome Scale; pGCL, peripapillary GCL; pIPL, peripapillary IPL; pRNFL, peripapillary retinal nerve fiber layer; SCID-NP, Structured Clinical Interview for DSM-IV Axis I Disorders Non-Patient Version; sd, standard deviation; SGAs, second-generation antipsychotics; SPH, optical spherical equivalent; SSD, schizophrenia spectrum disorder; SZ, schizophrenia; SZA, schizophrenia affective disorder; BPD. brief psychotic disorder; TRS, treatment-resistant schizophrenia; DD, delusional disorder; VHs, visual hallucinations.

# **Table S2. OCTA Study characteristics in a systematic review and meta-analysis.**

|  |  |  |  | SSD participants |  | Controls |  |  |  |
| --- | --- | --- | --- | --- | --- | --- | --- | --- | --- |
| Study | Study design | Participants | No. of eyes for OCT studies | Men (%) | Age (sd) | Men (%) | Age (sd) | Duration of illness (months, sd) | Country |
| Bannai et al. 2022^62^ | Cases and controls | 26 participants with SZ (n = 18)/SZA (n = 8) and 21 HCs. Participants with SSDs were divided into early course (n = 8; duration of illness <5 years) and chronic (n = 15; duration of illness >5 years) | 52 eyes in SSDs, 42 eyes in HCs (both eyes) | 76.9 | 37.0 (12.7) | 66.7 | 38.0 (11.6) | 163.6 (153.1) | USA |
| Boudriot et al. 2022^18^ | Cases and controls | 65 participants with SSDs (47 SZ, 17 SZA, 1 BPD) and 89 HCs | 126 eyes in SSDs and 143 eyes in HCs (both eyes) | 64.6 | 39.29 (10.8) | 50 | 34.65 (11.35) | 164.28 (93.72) | Germany |
| Hanifi Kokaçya et al. 2022^24^ | Cases and controls | 47 participants with SZ and 50 age- and sex-matched HCs | 47 eyes in SZ and 50 eyes in HCs (one eye) | 76.6 | 34.74 (10.68) | 70 | 34.02 (10.30) | NA | Turkey |
| Koman-Wierdak et al. 2021^29^ | Cases and controls | 12 participants with SZ, 8 participants with BDs, and 15 age- and sex-matched HCs | 24 eyes in SZ, 16 eyes in BDs, and 30 eyes in HCs (both eyes) | 50 | 26.33 (5.26) | 46.67 | 26.8 (-) | 42.58 (42.01) | Poland |
| Budakoglu et al. 2021^38^ | Cases and controls | 22 participants with SZ and 26 age- and sex-matched HCs | 22 eyes in SZ and 26 eyes in HCs (one eye) | 64 | 40.8 (7.9) | 69 | 45.2 (8.1) | 99.6 (39.6) | Turkey |
| Silverstein et al. 2021^34^ | Cases and controls | 28 participants with SZ or SZA and 37 HCs | 49-46 eyes in SDDs and 70 eyes in HCs (both eye) | 86 | 32.21 (11.27) | 65 | 32.22 (12.63) | NA | USA |

**Table S2. OCTA Study characteristics in a systematic review and meta-analysis (continued).**

| Study | OCT device type | OCT device model | OCT manufacturer | Diagnostic criteria | Exclusion criteria | Measurements | Main findings | NOS scores (S/C/E) |
| --- | --- | --- | --- | --- | --- | --- | --- | --- |
| Bannai et al. 2022 | SS-OCT | DRI Topcon Triton | Topcon Inc | DSM-IV TR | History of substance dependence/abuse within the past 6 months, glaucoma, macular degeneration, retinal occlusions, ocular trauma or myopia >4.0 diopters, current pregnancy/breast-feeding, head injury with neurologic sequelae, intellectual disability, or a history of neurologic disorders | VD, SVD, VDI, FD in superficial, deep, and choriocapillaris vascular regions of macula and FAZ | Higher overall superficial SVD, choriocapillaris VD, and choriocapillaris SVD in right eye, primarily in the central, central and outer superior, and central and outer inferior/superior macula in participants with SZ. Higher superficial VD, choriocapillaris SVD, and choriocapillaris FD in the right eye in early-course (duration of illness <5 years) participants with SZ compared with HCs. Negative correlation between bilateral superficial VD with PANSS positive scores and positive correlation between deep VDI and PANSS negative scores in the left eye. | 6 (2/2/2) |
| Boudriot et al. 2022 | SD-OCT | Cirrus HD-OCT 5000 | Carl Zeiss Inc | DSM-5 | Age <18 years or >65 years; concurrent clinically relevant central nervous system disorder; history of encephalitis, meningitis, or stroke; retinal pathology; IOP ≥21 mm Hg; pregnancy; refractive error ≥±6 SPH D | Superficial PD and VD in the central and inner ring macular region, superficial perfusion density in peripapillary region, and FAZ | No significant difference between participants with SSDs and HCs in macular and peripapillary PD, macular VD, or size of FAZ | 9 (4/2/3) |
| Hanifi Kokaçya et al. 2022 | SD-OCT | Optovue RTVue-XR Avanti OCT | Optovue Inc | DSM-5 | Age <18 years, visual acuity <20/25 and refractive error ≥2 D, history of pregnancy, substance use, systemic disease, ocular disease, or previous ocular trauma and intraocular surgery | Superficial and deep VD in whole, foveal, parafoveal, and perifoveal macula. All and small VD in radial peripapillary capillary and FZA. | Lower VD of superficial and deep capillary plexus in whole and perifoveal region and lower VD of radial peripapillary capillaries in whole disc and superior hemisphere in participants with SZ compared with HCs. Lower VD of superficial capillary plexus in the whole and perifoveal region; of the deep capillary plexus in the whole, perifoveal, and foveal region; and lower VD of the radial peripapillary capillaries in the whole disc and inside disc in long-term SZ participants compared with short-term SZ participants. | 6 (2/2/2) |
| Koman-Wierdak et al. 2021 | SD-OCT | Optovue OCT | Optovue Inc | ICD-10 | Systemic diseases (diabetes mellitus, cardiovascular disorders), alcohol and other psychoactive substance dependence, any eye disease (glaucoma, macular abnormalities, high myopia, ocular trauma, retinal detachment), other diseases that may affect OCT or OCTA measurements | Superficial and deep VD in whole and foveal macula. Whole, superior, nasal, inferior, and temporal VD in the radial peripapillary capillary. | Lower VD of the deep vascular complex in the whole macula and lower VD of the radial peripapillary capillary in the whole disc in participants with SZ compared with BD participants and HCs. | 7 (3/2/2) |
| Budakoglu et al. 2021 | SD-OCT | Optovue RTVue XR Avanti OCT | Optovue Inc | DSM IV-TR | Pathology in the anterior segment and/or posterior segment examination, corrected IOP >21 mm Hg, refractive error ≥±1 SPH/CYL D. Any systemic disease that affects the retinal vascular structures, such as diabetes mellitus or essential hypertension, media opacity that will affect imaging, participants who underwent ocular surgery, and those younger than 18 years. | Whole, inside disk, peripapillary, superior nasal, inferior, and temporal VD in the radial peripapillary capillary | Lower peripapillary VD values and pRNFL thickness in the temporal quadrant in the participants with SZ than in HCs | 6 (3/1/2) |
| Silverstein et al. 2021 | SD-OCT | Cirrus HD-OCT 5000 | Carl Zeiss Inc | NA | History of eye injury or disease (e.g., cataracts, macular degeneration, diabetic retinopathy, glaucoma); neurologic, intellectual, mood, or developmental disorders; history of head injury with loss of consciousness of more than 10 minutes; electroconvulsive therapy within the past 8 weeks; amblyopia (lazy eye) or a squint; and diabetes or high blood pressure | Superficial PD and VD in the macular region and FAZ | In the 3 × 3 mm^2^ regions centered on FAZ, lower superficial VD in the left eye and PD and enlarged FAZ in both eyes in participants with SSDs. The microvascular abnormalities were generally associated with scores on a proxy measure of verbal IQ. Positive correlation between left PD, left VD, right PD, right VD and WTAR scores and negative correlation between left FAZ size and right FAZ size and WTAR scores. | 6 (2/2/2) |

Abbreviations: BD, bipolar disorder; CYL, optical cylindrical equivalent; D, diopter; DSM-IV, *Diagnostic and Statistical Manual of Mental Disorders, Fourth Edition*; DSM-IV-TR, *Diagnostic and Statistical Manual of Mental Disorders, Fourth Edition, Text Revision*; FAZ, foveal avascular zone; FD, fractal dimension; HC, healthy control; ICD-10, International Statistical Classification of Diseases and Related Health Problems-10; IQ, intelligence quotient; IOP, intraocular pressure; NOS, Newcastle–Ottawa Scale; OCT, optical coherence tomography; OCTA, OCT angiography; PANSS, Positive and Negative Syndrome Scale; PD, perfusion density; pRNFL, peripapillary retinal nerve fiber layer; sd, standard deviation; SD, spectrum domain; SDD, ; SPH, optical spherical equivalent; SS, swept source; SSD, schizophrenia spectrum disorder; SVD, skeletonized vessel density; SZ, schizophrenia; SZA, schizophrenia affective disorder; VD, vessel density; VDI, vessel diameter index; WTAR, Wechsler Test of Adult Reading.

# **Table S3. ERG study characteristics in a systematic review and meta-analysis.**

|  |  |  | SSD participants |  | Controls |  |  |  |
| --- | --- | --- | --- | --- | --- | --- | --- | --- |
| Study | Study design | Participants | Men (%) | Age (sd) | Men (%) | Age (sd) | Duration of illness (months, sd) | Country |
| Friedel et al. 2022^20^ | Cases and controls | 25 paranoid SZ and 25 age- and sex-matched HCs | 40 | 39 (12) | 40 | 37 (12) | 108 (96) | Germany |
| Bernardin et al. 2022^63^ | Cases and controls | 29 SZ and 29 age- and sex-matched HCs | 79 | 28 (8.2) | 83 | 25 (5.5) | 85.5 (100.2) | France |
| Bernardin et al. 2021a^64^ | Cases and controls | 12 participants with SZ with visual hallucinations (VH+) and 17 participants with SZ with auditory hallucinations or no hallucinations (VH−) | 79.7 | 28.1 (9.3) | NA | NA | 85.5 (104.7) | France |
| Bernardin et al. 2021b^65^ | Cases and controls | 29 participants with SZ with the presence of visual hallucinations (VH) or with AHNH, one young adult with CHR, and 29 healthy matched controls | NA | NA | NA | NA | NA | France |
| Moghimi et al. 2020^66^ | Cases and controls | 30 SZ and 34 age- and sex-matched HCs | 53.3 | 40.27 (11.99) | 51.4 | 39.97 (13.0) | NA | USA |
| Hébert et al. 2020^67^ | Cases and controls | 150 SZ, 151 BD, and 200 HCs | 81 | 39.4 (9.9) | 62 | 39.4 (10.2) | 165.6 (110.4) | Canada |
| Fradkin et al. 2020^68^ | Cases and controls | 25 SZ and 24 HCs | 84 | 36.8 (10.83) | 75 | 32.13 (11.92) | NA | USA |
| Demmin et al. 2020a^69^ | Cases and controls | 25 SZ, 25 MDD, and 25 HCs | 84 | 36.80 (10.83) | 72 | 32.60 (11.91) | NA | USA |
| Demmin et al. 2020b^70^ | Cases and controls | 15 SZ or SZA and 15 HCs | 73.3 | 38.20 (10.19) | 86.7 | 38.13 (14.05) | NA | USA |
| Bernardin et al. 2020^71^ | Cases and controls | 29 SZ with VH (n = 12) or with AHNH (n = 17) and 29 sex-matched HCs | 79 | 28 (8.24) | 83 | 25 (5.50) | NA | France |
| Demmin et al. 2018^72^ | Cases and controls | 25 SZ and 25 HCs | 84 | 36.80 (10.83) | 72 | 32.60 (11.91) | NA | USA |
| Hébert et al. 2015^73^ | Cases and controls | 105 SZ and 150 HCs | 79 | 40.1 (9.9) | 62 | 40.6 (9.5) | 170.4 (111.6) | Canada |
| Balogh et al. 2008^74^ | Follow-up design | 26 SZ, 17 BD type I, and 20 HCs | 73 | 39.4 (8.2) | 75 | 38.6 (9.4) | 152.4 (60) | Hungary |
| Warner et al. 1999^75^ | Cases and controls | 9 SZ and 9 age- and sex-matched HCs | 55.6 | 52 (—) | 55.6 | 52 (—) | NA | UK |
| Gerbaldo et al. 1992^76^ | Cases and controls | 9 SZ and 13 HCs | NA | 36 (6) | NA | 39 (8) | NA | USA |
| Marmor et al. 1988^77^ | Cases and controls | 12 male SZ and 9 male HCs | 100 | NA | 100 | NA | NA | USA |
| Raese et al. 1982^78^ | Cases and controls | 11 male SZ or SZA and 11 HCs | 100 | NA | 100 | NA | NA | USA |

**Table S3. ERG study characteristics in a systematic review and meta-analysis (continued).**

| Study | ERG device type | ERG device model | ERG manufacturer | Diagnostic criteria | Exclusion criteria | ERG | Measurements | Main findings | NOS scores (S/C/E) |
| --- | --- | --- | --- | --- | --- | --- | --- | --- | --- |
| Friedel et al. 2022 | Portable | The RETeval | LKC Technologies, Inc | ICD-10 | Age <18 or > 65 years, neurologic or ophthalmologic diseases, myopia <−7 D or hyperopia >+7 D, substance abuse, or diabetes mellitus | Photopic fERG and PhNR-fERG | Amplitude of a- and b-wave and PhNR in photopic condition | Reduced amplitude of a-wave and PhNR in participants with SZ | 7 (3/2/2) |
| Bernardin et al. 2022 | Nonportable | The MonPackOne system | Metrovision | DSM IV-TR | Participants with abnormal findings on visual acuity test or fundus examination, ophthalmologic symptoms, or media opacity | Photopic and scotopic fERG and photopic Flicker ERG and mfERG | Amplitude and implicit time of N1, P1, and N2 in mfERG and amplitude and implicit time of OP1-4 in the scotopic condition | In participants with SZ, reduced amplitudes of P1 and N2 in the <2◦ region, N1 and P1 in the 2–5◦ region, and P1 in the 5–10◦ region in the center of the retina. Reduced amplitude of the O1, O2, O3, and overall O1, O2, and O3 in the scotopic condition. Prolonged implicit time of N2 in the <2◦ region, P1 in the 5–10◦ region, and N1 and P1 in the 10–15◦ region in the center of the retina. | 6 (3/1/2) |
| Bernardin et al. 2021a | Nonportable | The MonPackOne system | Metrovision | DSM IV-TR | History of neurologic disease | PERG | Amplitude and implicit time of P50 and N95 | Associations between the amplitudes of the P50 and the N95 and visual cognition. No difference between participants with VH+ and participants with VH− in the amplitudes and implicit time of both P50 and the N95. | 4 (2/0/2) |
| Bernardin et al. 2021b | Nonportable | The MonPackOne system | Metrovision | DSM IV-TR, CAARMS | History of neurologic disease | Photopic and scotopic fERG and PERG | Implicit time of b-wave in the scotopic condition | CHR showed prolonged b-wave implicit time compared with HCs and an intermediate level in the implicit time between participants with VH and participants with AHNH | 6 (3/1/2) |
| Moghimi et al, 2020 | Nonportable | Diagnosys LLC system | Diagnosys LLC | DSM-IV | History of eye disease that would affect the ERG data | PhNR-fERG and PERG | Amplitude and implicit time of a- and b-wave and PhNR in the photopic condition. Amplitude of N35, P50, and N95 in PERG. | Increased amplitude of PhNR and N72 at the highest flash (7 cds/m^2^) in participants with SZ. Increased power of high-frequency OPs (100–300 Hz) and combined OPs (75–300 Hz) in males. | 6 (3/1/2) |
| Hébert et al. 2020 | Nonportable | Espion (E2, E3) systems | Diagnosys LLC | DSM-IV | Participants with no normal vision with ocular pathology or brain and metabolic disorders, being pregnant, having used drugs including cannabis in the past 24 hours, or having traveled two time zones within 1 month before the experiment or working night shifts | Photopic and scotopic fERG | Amplitude and implicit time of a- and b-wave in the photopic and scotopic conditions | In photopic ERG, reduced amplitude of cone a-wave and prolonged b-wave implicit time were observed in both SZ and BD. Whereas a reduced amplitude of cone b-wave was present in schizophrenia. In scotopic ERG, reduced mixed rod-cone a- and b-wave amplitude were observed in both disorders. | 7 (4/1/2) |
| Fradkin et al. 2020 | Portable | The RETeval | LKC Technologies, Inc | DSM-IV | Age <18 years or >60 years; presence of an active substance use disorder in the past 6 months; history of diseases known to affect vision (e.g., diabetes, hypertension); history of a visual condition or disease (e.g., strabismus, nystagmus, glaucoma) or an eye injury; history of serious head injury with loss of consciousness >10 minutes; or history of neurologic disease | Photopic and scotopic fERG | Intra-individual variability of amplitude of a- and b-wave in photopic and scotopic conditions | No difference between participants with SZ and HCs in intra-individual variability in fERG responses in either the photopic or scotopic conditions | 8 (4/2/2) |
| Demmin et al. 2020a | Portable | The RETeval | LKC Technologies, Inc | DSM–IV | Age <18 years or >60 years, active substance use disorder within the past 6 months, history of ocular disease or injury, or medical conditions known to affect vision (e.g., diabetes, hypertension) | Photopic fERG (P1, P2, P3, Pf), scotopic fERG (S1, S2, S3), and PhNR-fERG | Amplitude and implicit time of a-wave and b-wave in photopic and scotopic conditions and PhNR | Participants with SZ showed attenuated photopic and scotopic a-wave amplitude in comparison with HCs (i.e., P1, P2, P3, S3). In participants with SZ, photopic and scotopic b-wave amplitudes were reduced when compared with both HCs and participants with MDD. Prolonged photopic b-wave implicit time was observed in the participants with SZ relative to HCs (P3). The SZ participants showed reduced PhNR amplitudes in comparison with the HCs. | 8 (4/2/2) |
| Demmin et al. 2020b | Portable | The RETeval | LKC Technologies, Inc | DSM-5 | Substance use disorders within the past 6 months, diseases known to affect the eye or vision (such as diabetes, glaucoma, hypertension, macular degeneration), problems with fixation (e.g., strabismus, nystagmus), or dislike of the food item | Photopic fERG | Amplitude and implicit time of a-wave and b-wave under reword processing (baseline, anticipatory, and reward) in the photopic condition | Reduced a-wave and b-wave amplitude during baseline, anticipatory and reward conditions in participants with SZ or SZA | 8 (4/2/2) |
| Bernardin et al. 2020 | Nonportable | The MonPackOne system | Metrovision | DSM IV-TR | History of neurologic disease, participants with visual symptoms or any media opacities, drug or alcohol dependence | Photopic and scotopic fERG, PERG, and photopic Flicker ERG | Implicit time of P50 and N95 in PERG, a- and b-wave amplitude in the scotopic condition, and a-wave amplitude and implicit time in the photopic condition | Prolonged P50 and N95 implicit time in participants with SZ. In participants with SZ, reduced amplitude of a-wave and b-wave in the scotopic condition and reduced a-wave amplitude in the photopic condition. | 6 (3/1/2) |
| Demmin et al. 2018 | Portable | The RETeval | LKC Technologies, Inc | DSM-IV | Active substance use disorder within the past 6 months, diseases known to affect vision (such as diabetes, hypertension, macular degeneration), or problems with fixation (e.g., strabismus, nystagmus) | Photopic fERG (P1, P2, Pf), scotopic fERG (S1, S2, S3), and PhNR-fERG | Implicit time and amplitude of a- and b-wave in photopic condition and PhNR. Implicit time and amplitude of a- and b-wave in the scotopic condition. | Participants with SZ showed attenuated photopic and scotopic a-wave amplitude in comparison with the HCs (P1, S3). In participants with SZ, photopic and scotopic b-wave amplitudes were reduced when compared with HCs (P1, P2, S2, S3). The participants with SZ showed reduced Pf and PhNR amplitudes in comparison with the HCs. Prolonged implicit time of b-wave in SZ participants in comparison with the HCs (P2). | 8 (4/2/2) |
| Hébert et al. 2015 | Nonportable | Espion (E2, E3) systems | Diagnosys LLC | DSM-IV | NA | Photopic and scotopic fERG | Amplitude and implicit time of a- and b-wave in photopic and scotopic conditions | In photopic ERG, reduced amplitude of cone a-wave and b-wave and prolonged b-wave implicit time in participants with SZ. In scotopic ERG, reduced mixed rod-cone b-wave amplitude in participants with SZ. | 4 (2/0/2) |
| Balogh et al. 2008 | Nonportable | Nicolet Spirit software | Nicolet Biomedical, Inc | DSM-IV | Neurologic disorders, head injury, and psychoactive substance dependence | Photopic fERG | Amplitude and implicit time of a-wave and amplitude of b-wave in photopic condition | In the acute stage of the illness, participants with SZ exhibited decreased a-wave amplitude as compared with HCs. After an 8-week follow-up period, a-wave amplitude significantly increased in SZ participants, as clinical symptoms improved. | 8 (4/2/2) |
| Warner et al. 1999 | Nonportable | Nicolet Spirit software | Nicolet Biomedical, Inc | DSM-IV | NA | Photopic and scotopic fERG and photopic Flicker ERG | Amplitude and implicit time of a- and b-wave in the photopic and scotopic conditions | SZ participants exhibited reduced a- and b-wave amplitude the in scotopic condition and a-wave amplitude in the photopic condition | 6 (3/1/2) |
| Gerbaldo et al. 1992 | Nonportable | UTAS System | LKC Technologies, Inc | DSM-III-R | Major medical disorder | Photopic and scotopic fERG | b-wave amplitude in the photopic condition | Participants with SZ who had a history of sun gazing showed reduced b-wave amplitude in the photopic condition | 5 (3/0/2) |
| Marmor et al. 1988 | Nonportable | UTAS System | LKC Technologies, Inc | RDC | Known ocular pathology | Photopic and scotopic fERG | Amplitude and implicit time of a-wave, b-wave, and OPs in photopic and scotopic conditions | No difference between SZ participants and HCs in the amplitudes and implicit time of OPs, a- and b-wave in both photopic and scotopic conditions | 4 (2/0/2) |
| Raese et al. 1982 | Nonportable | Storage oscilloscope | Tektronics, Inc | RDC | NA | Scotopic fERG | OPs in the scotopic condition | Reduced variance of O[1], O[2] RA and increased variance of N[1] and N[3] RA in participants with SZ. Increased variance of ΔO[2] RLs and ΔN[2] and ΔN[3] RLs in participants with SZ | 4 (2/0/2) |

Abbreviations: AHNH, auditory hallucinations or no hallucinations; BD, bipolar disorder; CAARMS, Comprehensive Assessment of At Risk Mental State; CHR, clinical high risk for psychosis; DSM-Ⅲ-R, *Diagnostic and Statistical Manual of Mental Disorders, Third Edition*; DSM-IV, *Diagnostic and Statistical Manual of Mental Disorders, Fourth Edition*; DSM IV-TR, *Diagnostic and Statistical Manual of Mental Disorders, Fourth Edition, Text Revision*; DSM-5, *Diagnostic and Statistical Manual of Mental Disorders, Fifth Edition*; ERG, electroretinogram; fERG, flash ERG; MDD, major depressive disorder; HC, healthy control; ICD-10, *International Statistical Classification of Diseases and Related Health Problems, Tenth Revision*; mfERG, multifocal fERG; NA, not applicable; NOS, Newcastle–Ottawa Scale; OP, oscillatory potential; PERG, pattern ERG; PhNR, photopic negative response; RA, relative amplitude; RDC, research diagnostic criteria; RL, relative latency; SSD, schizophrenia spectrum disorder; SZ, schizophrenia; SZA, schizophrenia affective disorder; VH, visual hallucination.

# **Table S4. Results of the meta-analysis after removing the potential outliers identified by GOSH analysis in pRNFL.**

|  |  |  |  |  |  | Heterogeneity | | |
| --- | --- | --- | --- | --- | --- | --- | --- | --- |
| Retinal thickness | Analysis | No. of studies | Pooled estimates | 95% CI | *p* value | Q | I^2^ | *p* value |
| pRNFL average thickness | Main analysis | 26 | −0.295 | [−0.4500; −0.1400] | 0.0002 | 127.68 | 80.40% | <0.0001 |
|  | Inf. cases removed^1^ | 25 | −0.2616 | [−0.3886; −0.1346] | <0.0001 | 70.88 | 66.10% | <0.0001 |
|  |  |  |  |  |  |  |  |  |
| pRNFL thickness in four quadrants |  |  |  |  |  |  |  |  |
| Superior thickness | Main analysis | 17 | −0.3016 | [−0.4558; −0.1474] | 0.0001 | 58.17 | 72.50% | <0.0001 |
|  | Inf. cases removed^2^ | 13 | −0.2933 | [−0.4254; −0.1612] | <0.0001 | 21.02 | 42.90% | 0.0501 |
|  |  |  |  |  |  |  |  |  |
| Inferior thickness | Main analysis | 17 | −-0.3038 | [−0.4533; −0.1543] | <0.0001 | 54.76 | 70.80% | <0.0001 |
|  | Inf. cases removed^3^ | 14 | −0.2692 | [−0.4062; −0.1321] | 0.0001 | 24.82 | 47.60% | 0.0244 |
|  |  |  |  |  |  |  |  |  |
| Temporal thickness | Main analysis | 21 | −0.1977 | [−0.3189; −0.0765] | 0.0014 | 47.86 | 58.20% | 0.0004 |
|  | Inf. cases removed^4^ | 15 | −0.2609 | [−0.3924; −0.1294] | 0.0001 | 27.21 | 48.50% | 0.0181 |
|  |  |  |  |  |  |  |  |  |
| Nasal thickness | Main analysis | 22 | −0.1563 | [−0.2994; −0.0131] | 0.0324 | 72.92 | 71.20% | <0.0001 |
|  | Inf. cases removed^5^ | 20 | −0.0775 | [−0.1849; 0.0299] | 0.1572 | 33.9 | 43.90% | 0.0189 |

^1^Removed as outliers: Liu et al. 2021.

^2^Removed as outliers: Khalila et al. 2022, Jerotic et al. 2021, Liu et al. 2021, Silverstein et al. 2018.

^3^Removed as outliers: Bozali et al. 2022, Kaya et al. 2022, Liu et al. 2021.

^4^Removed as outliers: Kurtulmus et al. 2023, Hosak et al. 2020, Topcu-Yilmaz et al. 2019, Delibas et al. 2018, Silverstein et al. 2018, May-Yin Chu et al. 2012.

^5^Removed as outliers: Khalila et al. 2022, Yilmaz et al. 2016.

Abbreviations: CI, confidence interval; GOSH: Graphical display of study heterogeneity; pRNFL: Peripapillary retinal nerve fiber layer.

# **Table S5. Results of publication bias and publication bias-adjusted meta-analysis in OCT parameters.**

|  |  | Egger’s regression test |  |  |  |  | Trim and fill method |  |  |  |  |
| --- | --- | --- | --- | --- | --- | --- | --- | --- | --- | --- | --- |
| OCT parameter | No. of studies | Intercept | SE | *t* value | *p* value |  | No. of outliers removed | No. of imputed studies | Pooled estimates | 95% CI | *p* value |
| pRNFL average thickness | 26 | −0.765 | 0.2403 | 1.79 | 0.0858 |  | — | — | — | — | — |
|  |  |  |  |  |  |  |  |  |  |  |  |
| pRNFL thickness in four quadrants |  |  |  |  |  |  |  |  |  |  |  |
| Superior thickness | 17 | −0.62 | 0.2222 | 1.3 | 0.2128 |  | — | — | — | — | — |
| Inferior thickness | 17 | −0.7527 | 0.2052 | 2 | 0.0645 |  | — | — | — | — | — |
| Temporal thickness | 21 | 0.1989 | 0.2539 | -1.52 | 0.1456 |  | — | — | — | — | — |
| Nasal thickness | 22 | 0.0331 | 0.3205 | -0.56 | 0.5824 |  | — | — | — | — | — |
|  |  |  |  |  |  |  |  |  |  |  |  |
| Macular average thickness | 11 | 0.1033 | 0.1200 | −3.62 | 0.0056 |  | 1 | 4 | −0.2509 | [−0.5479; 0.0461] | 0.0978 |
| Macular volume | 14 | −0.3992 | 0.1423 | −0.33 | 0.747 |  | — | — | — | — | — |
|  |  |  |  |  |  |  |  |  |  |  |  |
| Macular thickness in ETDRS grid |  |  |  |  |  |  |  |  |  |  |  |
| Central foveal thickness | 20 | 0.3123 | 0.2227 | −2.62 | 0.0172 |  | 3 | 5 | −0.2172 | [−0.3406; −0.0939] | 0.0006 |
| Outer ring |  |  |  |  |  |  |  |  |  |  |  |
| Superior thickness | 11 | −0.2498 | 0.2687 | −0.99 | 0.3472 |  | 1 | 1 | −0.417 | [−0.6096; −0.2244] | <0.0001 |
| Inferior thickness | 11 | −0.1309 | 0.2095 | −1.6 | 0.1441 |  | 1 | 3 | −0.4317 | [−0.6277; −0.2358] | <0.0001 |
| Temporal thickness | 9 | — |  |  |  |  | 1 | 1 | -0.3465 | [-0.5157; -0.1773] | < 0.0001 |
| Nasal thickness | 10 | -0.1963 | 0.226 | -1.36 | 0.2119 |  | 2 | 2 | −0.4507 | [−0.6060; −0.2953] | <0.0001 |
| Inner ring |  |  |  |  |  |  |  |  |  |  |  |
| Superior thickness | 11 | −0.5024 | 0.2085 | −0.23 | 0.8239 |  | 1 | 0 | −0.6036 | [−0.7421; −0.4650] | <0.0001 |
| Inferior thickness | 11 | −0.3446 | 0.1711 | −1.04 | 0.3251 |  | 1 | 2 | −0.5072 | [−0.6585; −0.3560] | <0.0001 |
| Temporal thickness | 10 | −0.3345 | 0.2023 | −0.92 | 0.3844 |  | 1 | 2 | −0.5153 | [−0.6851; −0.3454] | <0.0001 |
| Nasal thickness | 10 | −0.0849 | 0.1922 | −1.95 | 0.0863 |  | 2 | 3 | −0.3827 | [−0.5629; −0.2024] | <0.0001 |
|  |  |  |  |  |  |  |  |  |  |  |  |
| Macular GCL-IPL | 9 | — | — | — | — |  | 1 | 0 | −0.3613 | [−0.5036; −0.2191] | <0.0001 |
|  |  |  |  |  |  |  |  |  |  |  |  |
| Optic cup |  |  |  |  |  |  |  |  |  |  |  |
| Optic cup volume | 4 | — | — | — | — |  | 0 | 1 | 0.1753 | [−0.0557; 0.4063] | 0.137 |
| Cup-to-disc area ratio | 5 | — | — | — | — |  | 0 | 0 | 0.2719 | [−0.0565; 0.6004] | 0.1046 |
|  |  |  |  |  |  |  |  |  |  |  |  |
| FAZ | 3 | — | — | — | — |  | 0 | 0 | 0.2355 | [−0.1105; 0.5814] | 0.1822 |
| Superficial foveal vessel density | 3 | — | — | — | — |  | 0 | 2 | -0.6327 | [−1.3389; 0.0735] | 0.0791 |

Abbreviations: CI, confidence interval; ETDRS, Early Treatment Diabetic Retinopathy Study; FAZ, foveal avascular zone; GCL-IPL, ganglion cell layer–inner plexiform layer; OCT, optical coherence tomography; pRNFL, peripapillary retinal nerve fiber layer.

# **Table S6. Results of GRADE for rating the quality of a body of evidence.**

| **Quality assessment** | | | | | | | |
| --- | --- | --- | --- | --- | --- | --- | --- |
| **Outcome** | **Participants (studies)** | **Risk of bias** | **Inconsistency** | **Indirectness** | **Imprecision** | **Publication bias** | **Overall quality of evidence** |
| pRNFL average thickness | 2,078 (26 studies) | No serious risk of bias | Serious^1^ | No serious indirectness | No serious imprecision | Undetected | ⊕⊝⊝⊝ **VERY LOW**^1^ due to inconsistency |
| pRNFL superior thickness | 1,488 (17 studies) | No serious risk of bias | Serious^2^ | No serious indirectness | No serious imprecision | Undetected | ⊕⊝⊝⊝ **VERY LOW**^2^ due to inconsistency |
| pRNFL inferior thickness | 1,488 (17 studies) | No serious risk of bias | Serious^3^ | No serious indirectness | No serious imprecision | Undetected | ⊕⊝⊝⊝ **VERY LOW**^3^ due to inconsistency |
| pRNFL temporal thickness | 1,597 (21 studies) | No serious risk of bias | Serious^4^ | No serious indirectness | No serious imprecision | Undetected | ⊕⊝⊝⊝ **VERY LOW**^4^ due to inconsistency |
| pRNFL nasal thickness | 1,694 (22 studies^1^) | No serious risk of bias | Serious^5^ | No serious indirectness | No serious imprecision | Undetected | ⊕⊝⊝⊝ **VERY LOW**^5^ due to inconsistency |

^1^High heterogeneity (I^2^ = 80.4%).

^2^High heterogeneity (I^2^ = 72.5%).

^3^High heterogeneity (I^2^ = 70.8%).

^4^High heterogeneity (I^2^ = 58.2%).

^5^High heterogeneity (I^2^ = 71.2%).

|  | **Quality assessment** | | | | | | |
| --- | --- | --- | --- | --- | --- | --- | --- |
| **Outcome** | **Participants (studies)** | **Risk of bias** | **Inconsistency** | **Indirectness** | **Imprecision** | **Publication bias** | **Overall quality of evidence** |
| Macular average thickness | 98,697 (11 studies) | No serious risk of bias | Serious^1^ | No serious indirectness | No serious imprecision | Reporting bias strongly suspected | ⊕⊝⊝⊝ **VERY LOW**^1^ due to inconsistency, publication bias, large effect |
| Macular superior thickness in the outer ring | 963 (11 studies) | No serious risk of bias | Serious^2^ | No serious indirectness | No serious imprecision | Undetected | ⊕⊝⊝⊝ **VERY LOW**^2^ due to inconsistency |
| Macular inferior thickness in the outer ring | 963 (11 studies) | No serious risk of bias | Serious^3^ | No serious indirectness | No serious imprecision | Undetected | ⊕⊝⊝⊝ **VERY LOW**^3^ due to inconsistency |
| Macular temporal thickness in the outer ring | 773 (9 studies) | No serious risk of bias | Serious^4^ | No serious indirectness | No serious imprecision | Undetected | ⊕⊝⊝⊝ **VERY LOW**^4^ due to inconsistency |
| Macular nasal thickness in the outer ring | 870 (10 studies) | No serious risk of bias | Serious^5^ | No serious indirectness | No serious imprecision | Undetected | ⊕⊝⊝⊝ **VERY LOW**^5^ due to inconsistency |
| Macular superior thickness in the inner ring | 1036 (11 studies) | No serious risk of bias | Serious^6^ | No serious indirectness | No serious imprecision | Undetected | ⊕⊝⊝⊝ **VERY LOW**^6^ due to inconsistency |
| Macular inferior thickness in the inner ring | 1,036 (11 studies) | No serious risk of bias | Serious^7^ | No serious indirectness | No serious imprecision | Undetected | ⊕⊝⊝⊝ **VERY LOW**^7^ due to inconsistency |
| Macular temporal thickness in the inner ring | 943 (10 studies) | No serious risk of bias | Serious^8^ | No serious indirectness | No serious imprecision | Undetected | ⊕⊝⊝⊝ **VERY LOW**^8^ due to inconsistency |
| Macular nasal thickness in the inner ring | 943 (10 studies) | No serious risk of bias | Serious^9^ | No serious indirectness | No serious imprecision | Undetected | ⊕⊝⊝⊝ **VERY LOW**^9^ due to inconsistency |
| Macular central foveal thickness | 2,603 (20 studies) | No serious risk of bias | Serious^10^ | No serious indirectness | No serious imprecision | Reporting bias strongly suspected^11^ | ⊕⊝⊝⊝ **VERY LOW**^10,11^ due to inconsistency, publication bias |

^1^High heterogeneity (I^2^ = 84.1%).

^2^High heterogeneity (I^2^ = 75.7%).

^3^High heterogeneity (I^2^ = 66%).

^4^High heterogeneity (I^2^ = 54.1%).

^5^High heterogeneity (I^2^ = 68.6%).

^6^High heterogeneity (I^2^ = 58.4%).

^7^High heterogeneity (I^2^ = 45.6%).

^8^High heterogeneity (I^2^ = 59.8%).

^9^High heterogeneity (I^2^ = 67.9%).

^10^High heterogeneity (I^2^ = 81.9%).

^11^Egger’s regression test showed significant publication bias (*t* = −2.62, *p* = 0.0172).

| **Quality assessment** | | | | | | | |
| --- | --- | --- | --- | --- | --- | --- | --- |
| **Outcome** | **Participants (studies)** | **Risk of bias** | **Inconsistency** | **Indirectness** | **Imprecision** | **Publication bias** | **Overall quality of evidence** |
| Macular volume | 98,793 (14 studies) | No serious risk of bias | Serious^1^ | No serious indirectness | No serious imprecision | Undetected | ⊕⊝⊝⊝ **VERY LOW**^1^ due to inconsistency |

^1^High heterogeneity (I^2^ = 68.1%).

| **Quality assessment** | | | | | | | |
| --- | --- | --- | --- | --- | --- | --- | --- |
| **Outcome** | **Participants (studies)** | **Risk of bias** | **Inconsistency** | **Indirectness** | **Imprecision** | **Publication bias** | **Overall quality of evidence** |
| mGCL-IPL | 102,027 (9 studies) | No serious risk of bias | Serious^1^ | No serious indirectness | No serious imprecision | Undetected | ⊕⊝⊝⊝ **VERY LOW**^1^ due to inconsistency |

^1^High heterogeneity (I^2^ = 68.1%).

| **Quality assessment** | | | | | | | |
| --- | --- | --- | --- | --- | --- | --- | --- |
| **Outcome** | **Participants (studies) Follow up** | **Risk of bias** | **Inconsistency** | **Indirectness** | **Imprecision** | **Publication bias** | **Overall quality of evidence** |
| Optic cup | 536 (4 studies) | No serious risk of bias | Serious^1^ | No serious indirectness | No serious imprecision | Undetected | ⊕⊝⊝⊝ **VERY LOW**^1^ due to inconsistency |

^1^High heterogeneity (I^2^ = 57.1%).

| **Quality assessment** | | | | | | | |
| --- | --- | --- | --- | --- | --- | --- | --- |
| **Outcome** | **Participants (studies)** | **Risk of bias** | **Inconsistency** | **Indirectness** | **Imprecision** | **Publication bias** | **Overall quality of evidence** |
| Photopic fERG a-wave amplitude | 568 (6 studies) | No serious risk of bias | Serious^1^ | No serious indirectness | No serious imprecision | Undetected | ⊕⊕⊝⊝ **LOW**^1,2^ due to inconsistency, large effect |
| Photopic fERG b-wave amplitude | 624 (7 studies) | No serious risk of bias | No serious inconsistency | No serious indirectness | No serious imprecision | Undetected | ⊕⊕⊝⊝ **LOW** |
| Photopic fERG a-wave latency | 568 (6 studies) | No serious risk of bias | No serious inconsistency | No serious indirectness | No serious imprecision | Undetected | ⊕⊕⊝⊝ **LOW** |

^1^High heterogeneity (I^2^ = 76.5%).

^2^SMD > 0.7.

| **Quality assessment** | | | | | | | |
| --- | --- | --- | --- | --- | --- | --- | --- |
| **Outcome** | **Participants (studies)** | **Risk of bias** | **Inconsistency** | **Indirectness** | **Imprecision** | **Publication bias** | **Overall quality of evidence** |
| Scotopic fERG a-wave amplitude | 468 (4 studies) | No serious risk of bias | Serious^1^ | No serious indirectness | No serious imprecision | Undetected | ⊕⊕⊝⊝ **LOW**^1,2^ due to inconsistency, large effect |
| Scotopic fERG b-wave amplitude | 468 (4 studies) | No serious risk of bias | No serious inconsistency | No serious indirectness | No serious imprecision | Undetected | ⊕⊕⊕⊝ **MODERATE**^2^ due to large effect |

^1^High heterogeneity (I^2^ = 74.1%).

^2^SMD > 0.7.

Abbreviations: fERG, flash electroretinogram; GRADE, Grading of Recommendations, Assessment, Development, and Evaluations; mGCL-IPL, macular ganglion cell layer with inner plexiform layer; pRNF, peripapillary retinal nerve fiber layer.

# **Table S7. Results of the meta-analysis after removing the potential outliers identified by GOSH analysis in the macula.**

|  |  |  |  |  |  | Heterogeneity | | |
| --- | --- | --- | --- | --- | --- | --- | --- | --- |
| Retinal thickness | Analysis | No. of studies | Pooled estimates | 95% CI | *p* value | Q | I^2^ | *p* value |
| Macular average thickness | Main analysis | 11 | −0.5153 | [−0.7426; −0.2881] | <0.0001 | 62.77 | 84.10% | <0.0001 |
|  | Inf. cases removed^1^ | 10 | −0.5802 | [−0.8494; −0.3110] | <0.0001 | 49.26 | 81.70% | <0.0001 |
|  |  |  |  |  |  |  |  |  |
| Macular volume | Main analysis | 14 | −0.4489 | [−0.5971; −0.3006] | <0.0001 | 40.81 | 68.1% | 0.0001 |
|  | Inf. cases removed^2^ | 11 | −0.4811 | [−0.5645; −0.3976] | <0.0001 | 10.52 | 4.90% | 0.3961 |
|  |  |  |  |  |  |  |  |  |
| Macular thickness in ETDRS grid |  |  |  |  |  |  |  |  |
| Central foveal thickness | Main analysis | 20 | −0.3298 | [−0.5006; −0.1590] | 0.0002 | 105.24 | 82% | <0.0001 |
|  | Inf. cases removed^3^ | 17 | −0.3176 | [−0.4286; −0.2065] | <0.0001 | 33.43 | 52.10% | 0.0065 |
| Outer ring |  |  |  |  |  |  |  |  |
| Superior thickness | Main analysis | 11 | −0.5607 | [−0.7837; −0.3377] | <0.0001 | 41.21 | 75.70% | <0.0001 |
|  | Inf. cases removed^4^ | 10 | −0.4651 | [−0.6444; −0.2859] | <0.0001 | 21.3 | 57.70% | 0.0114 |
|  |  |  |  |  |  |  |  |  |
| Inferior thickness | Main analysis | 11 | −0.5091 | [−0.6980; −0.3202] | <0.0001 | 29.42 | 66.00% | 0.0011 |
|  | Inf. cases removed^5^ | 10 | −0.5609 | [−0.7426; −0.3792] | <0.0001 | 20.89 | 56.90% | 0.0131 |
|  |  |  |  |  |  |  |  |  |
| Temporal thickness | Main analysis | 9 | −0.4435 | [−0.6271; −0.2599] | <0.0001 | 17.44 | 54.10% | 0.0258 |
|  | Inf. cases removed^6^ | 8 | −0.3803 | [−0.5360; −0.2247] | <0.0001 | 11.08 | 36.80% | 0.1353 |
|  |  |  |  |  |  |  |  |  |
| Nasal thickness | Main analysis | 10 | −0.5495 | [−0.7616; −0.3373] | <0.0001 | 28.67 | 68.60% | 0.0007 |
|  | Inf. cases removed^7^ | 8 | −0.5068 | [−0.6509; −0.3627] | <0.0001 | 9.1 | 23.10% | 0.2455 |
|  |  |  |  |  |  |  |  |  |
| Inner ring |  |  |  |  |  |  |  |  |
| Superior thickness | Main analysis | 11 | −0.5509 | [−0.7156; −0.3862] | <0.0001 | 24.02 | 58% | 0.0075 |
|  | Inf. cases removed^8^ | 10 | −0.6036 | [−0.7421; −0.4650] | <0.0001 | 13.56 | 33.60% | 0.1387 |
|  |  |  |  |  |  |  |  |  |
| Inferior thickness | Main analysis | 11 | −0.5312 | [−0.6749; −0.3875] | <0.0001 | 18.38 | 46% | 0.0489 |
|  | Inf. cases removed^9^ | 10 | −0.5682 | [−0.6922; −0.4442] | <0.0001 | 11.39 | 21.00% | 0.2501 |
|  |  |  |  |  |  |  |  |  |
| Temporal thickness | Main analysis | 10 | −0.54 | [−0.7195; −0.3606] | <0.0001 | 22.41 | 60% | 0.0077 |
|  | Inf. cases removed^10^ | 9 | −0.5891 | [−0.7336; −0.4447] | <0.0001 | 11.74 | 31.90% | 0.1632 |
|  |  |  |  |  |  |  |  |  |
| Nasal thickness | Main analysis | 10 | −0.5155 | [−0.7155; −0.3156] | <0.0001 | 28.02 | 68% | 0.0009 |
|  | Inf. cases removed^11^ | 8 | −0.4979 | [−0.6583; −0.3376] | <0.0001 | 12.37 | 43.40% | 0.089 |

^1^Removed as outliers: Shoham et al. 2023.

^2^Removed as outliers: Hosak et al. 2020, Lee et al. 2013, May-Yin Chu et al. 2012.

^3^Removed as outliers: Asanad et al. 2022, Sarkar et al. 2021, Altun et al. 2020.

^4^Removed as outliers: Asanad et al. 2022.

^5^Removed as outliers: Asanad et al. 2022.

^6^Removed as outliers: Sarkar et al. 2021.

^7^Removed as outliers: Altun et al. 2020, Lee et al. 2013.

^8^Removed as outliers: Asanad et al. 2022.

^9^Removed as outliers: Asanad et al. 2022.

^10^Removed as outliers: Altun et al. 2020.

^11^Removed as outliers: Sarkar et al. 2021, Altun et al. 2020.

Abbreviations: CI, confidence interval. ETDRS: Early Treatment Diabetic Retinopathy Study; GOSH, graphical display of study heterogeneity.

# **Table S8. Results of the meta-analysis after removing the potential outliers identified by GOSH analysis in macular GCL-IPL and optic cup.**

|  |  |  |  |  |  | Heterogeneity | | |
| --- | --- | --- | --- | --- | --- | --- | --- | --- |
| Retinal thickness | Analysis | No. of studies | Pooled estimates | 95% CI | *p* value | Q | I^2^ | *p* value |
| Macular GCL-IPL | Main analysis | 9 | −0.4137 | [−0.5744; −0.2531] | <0.0001 | 25.09 | 68.10% | 0.0015 |
|  | Inf. cases removed^1^ | 8 | −0.3613 | [−0.5036; −0.2191] | <0.0001 | 14.14 | 50.50% | 0.0488 |
|  |  |  |  |  |  |  |  |  |
| Optic cup |  |  |  |  |  |  |  |  |
| Optic cup volume | Main analysis | 4 | 0.2577 | [0.0513; 0.4641] | 0.0144 | 7 | 57.1% | 0.0719 |
|  | Inf. cases removed | No outliers detected |  |  |  |  |  |  |
|  |  |  |  |  |  |  |  |  |
| Cup-to-disc area ratio | Main analysis | 5 | 0.2719 | [−0.0565; 0.6004] | 0.1046 | 13.45 | 0.703 | 0.1046 |
|  | Inf. cases removed | No outliers detected |  |  |  |  |  |  |

^1^Removed as outliers: Boudriot et al. 2022.

Abbreviations: CI, confidence interval; GCL-IPL, ganglion cell layer-inner plexiform layer; GOSH, graphical display of study heterogeneity.

# **Table S9. Results of the meta-analysis after removing the potential outliers identified by GOSH analysis in FAZ and superficial foveal vessel density.**

|  |  |  |  |  |  | Heterogeneity | |  |
| --- | --- | --- | --- | --- | --- | --- | --- | --- |
| OCTA parameter | Analysis | No. of studies | Pooled estimates | 95% CI | *p* value | Q | I^2^ | *p* value |
| FAZ | Main analysis | 3 | 0.2355 | [−0.1105; 0.5814] | 0.1822 | 6.04 | 66.90% | 0.0488 |
|  | Inf. cases removed | No outliers detected | |  |  |  |  |  |
|  |  |  |  |  |  |  |  |  |
| Superficial foveal vessel density | Main analysis | 3 | -0.0771 | [−0.6987; 0.5445] | 0.8079 | 12.24 | 84% | 0.0022 |
|  | Inf. cases removed | No outliers detected | |  |  |  |  |  |

Abbreviations: CI: confidence interval; FAZ, foveal avascular zone; OCTA, optical coherence tomography angiography.

# **Table S10. Results of the meta-analysis after removing the potential outliers identified by GOSH analysis in fERG.**

|  |  |  |  |  |  | Heterogeneity | |  |
| --- | --- | --- | --- | --- | --- | --- | --- | --- |
| fERG parameter | Analysis | No. of studies | Pooled estimates | 95% CI | *p* value | Q | I^2^ | *p* value |
| Amplitude |  |  |  |  |  |  |  |  |
| Photopic fERG |  |  |  |  |  |  |  |  |
| a-wave amplitude | Main analysis | 6 | −1.0292 | [−1.4894; −0.5691] | <0.0001 | 21.32 | 76.50% | 0.0007 |
|  | Inf. cases removed | No outliers detected |  |  |  |  |  |  |
|  |  |  |  |  |  |  |  |  |
| b-wave amplitude | Main analysis | 7 | −0.2785 | [−0.4373; −0.1197] | 0.0006 | 1.08 | 0% | 0.9826 |
|  | Inf. cases removed | No outliers detected |  |  |  |  |  |  |
|  |  |  |  |  |  |  |  |  |
| PhNR amplitude | Main analysis | 3 | −0.3175 | [−0.9465; 0.3115] | 0.3226 | 7.64 | 73.8% | 0.0219 |
|  | Inf. cases removed | No outliers detected |  |  |  |  |  |  |
|  |  |  |  |  |  |  |  |  |
| Scotopic fERG |  |  |  |  |  |  |  |  |
| a-wave amplitude | Main analysis | 4 | −0.8045 | [−1.3379; −0.2712] | 0.0031 | 11.59 | 74.1% | 0.0089 |
|  | Inf. cases removed | No outliers detected |  |  |  |  |  |  |
|  |  |  |  |  |  |  |  |  |
| b-wave amplitude | Main analysis | 4 | −0.7294 | [−1.0655; −0.3933] | <0.0001 | 5.13 | 41.6% | 0.1622 |
|  | Inf. cases removed | No outliers detected |  |  |  |  |  |  |
|  |  |  |  |  |  |  |  |  |
| Latency |  |  |  |  |  |  |  |  |
| Photopic fERG |  |  |  |  |  |  |  |  |
| a-wave latency | Main analysis | 6 | −0.2537 | [−0.4755; −0.0319] | 0.025 | 6.42 | 22.1% | 0.2676 |
|  | Inf. cases removed | No outliers detected |  |  |  |  |  |  |
|  |  |  |  |  |  |  |  |  |
| b-wave latency | Main analysis | 4 | 0.4474 | [−0.1553; 1.0501] | 0.1457 | 21.66 | 86.1% | <0.0001 |
|  | Inf. cases removed | No outliers detected |  |  |  |  |  |  |
|  |  |  |  |  |  |  |  |  |
| Scotopic fERG |  |  |  |  |  |  |  |  |
| a-wave latency | Main analysis | 4 | −0.0561 | [−0.2390; 0.1268] | 0.5477 | 2.78 | 0% | 0.4274 |
|  | Inf. cases removed | No outliers detected |  |  |  |  |  |  |
|  |  |  |  |  |  |  |  |  |
| b-wave latency | Main analysis | 3 | 0.1652 | [−0.1326; 0.4631] | 0.2769 | 3.16 | 36.6% | 0.2063 |
|  | Inf. cases removed | No outliers detected |  |  |  |  |  |  |

Abbreviations: CI, confidence interval; fERG, flash electroretinogram; GOSH, graphical display of study heterogeneity; PhNR, photopic negative response.

# **Table S11. Results of publication bias-adjusted meta-analysis in fERG parameters.**

|  |  | Trim-and-fill method |  |  |  |  |
| --- | --- | --- | --- | --- | --- | --- |
| fERG parameter | No. of studies | No. of outliers removed | No. of imputed studies | Pooled estimates | 95% CI | *p* value |
| Amplitude |  |  |  |  |  |  |
| Photopic fERG |  |  |  |  |  |  |
| a-wave amplitude | 6 | 0 | 3 | −0.6108 | [−1.0839; −0.1376] | 0.0114 |
| b-wave amplitude | 7 | 0 | 0 | −0.2785 | [−0.4373; −0.1197] | 0.0006 |
| PhNR amplitude | 3 | 0 | 0 | −0.3175 | [−0.9465; 0.3115] | 0.3226 |
| Scotopic fERG |  |  |  |  |  |  |
| a-wave amplitude | 4 | 0 | 1 | −0.6237 | [−1.1608; −0.0865] | 0.0229 |
| b-wave amplitude | 4 | 0 | 2 | −0.5486 | [−0.8946; −0.2025] | 0.0019 |
|  |  |  |  |  |  |  |
|  |  |  |  |  |  |  |
| Latency |  |  |  |  |  |  |
| Photopic fERG |  |  |  |  |  |  |
| a-wave latency | 6 | 0 | 1 | −0.2221 | [−0.4639; 0.0198] | 0.0719 |
| b-wave latency | 4 | 0 | 2 | 0.9253 | [0.3170; 1.5336] | 0.0029 |
| Scotopic fERG |  |  |  |  |  |  |
| a-wave latency | 4 | 0 | 0 | −0.0561 | [−0.2390; 0.1268] | 0.5477 |
| b-wave latency | 3 | 0 | 0 | 0.1652 | [−0.1326; 0.4631] | 0.2769 |

Abbreviations: CI, confidence interval; fERG, flash electroretinogram; PhNR, photopic negative response.

# **Table S12. Meta-regression analysis of the association between the explanatory variables and pooled estimates of fERG thicknesses.**

| Retinal parameters | No. of studies | Explanatory variables | Cofficients | 95% CI | SE | *p* value |
| --- | --- | --- | --- | --- | --- | --- |
| Amplitude |  |  |  |  |  |  |
| Photopic fERG |  |  |  |  |  |  |
| a-wave amplitude | 6 | SSD participant age | −0.0322 | [−0.0998; 0.0354] | 0.0345 | 0.3503 |
|  | 6 | Sex (% of male SSD participants) | 0.0095 | [−0.0207; 0.0397] | 0.0154 | 0.5381 |
|  | 3 | Duration of illness (M) | — |  |  |  |
|  | 3 | PANSS total score | — |  |  |  |
|  | 3 | PANSS positive scale score | — |  |  |  |
|  | 3 | PANSS negative scale score | — |  |  |  |
|  | 3 | PANSS general psychopathology scale score | — |  |  |  |
|  | 4 | Antipsychotic dose (chlorpromazine equivalent [mg/day]) | — |  |  |  |
|  | 6 | NOS | -0.1509 | [-0.7780; 0.4762] | 0.3200 | 0.6372 |
|  |  |  |  |  |  |  |
|  |  |  |  |  |  |  |
| b-wave amplitude | 7 | SSD participant age | 0.0071 | [−0.0246; 0.0388] | 0.0162 | 0.661 |
|  | 7 | Sex (% of male SSD participants) | −0.0003 | [−0.0122; 0.0116] | 0.0061 | 0.961 |
|  | 3 | Duration of illness (M) | — |  |  |  |
|  | 3 | PANSS total score | — |  |  |  |
|  | 3 | PANSS positive scale score | — |  |  |  |
|  | 3 | PANSS negative scale score | — |  |  |  |
|  | 3 | PANSS general psychopathology scale score | — |  |  |  |
|  | 3 | Antipsychotic dose (chlorpromazine equivalent [mg/day]) | — |  |  |  |
|  | 7 | NOS | -0.0814 | [-0.3459; 0.1832] | 0.1350 | 0.5466 |
|  |  |  |  |  |  |  |
| PhNR amplitude | 3 | SSD participant age | — |  |  |  |
|  | 3 | Sex (% of male SSD participants) | — |  |  |  |
|  | 1 | Duration of illness (M) | — |  |  |  |
|  | 1 | PANSS total score | — |  |  |  |
|  | 1 | PANSS positive scale score | — |  |  |  |
|  | 1 | PANSS negative scale score | — |  |  |  |
|  | 1 | PANSS general psychopathology scale score | — |  |  |  |
|  | 1 | Antipsychotic dose (chlorpromazine equivalent [mg/day]) | — |  |  |  |
|  | 3 | NOS | — |  |  |  |
|  |  |  |  |  |  |  |
| Scotopic fERG |  |  |  |  |  |  |
| a-wave amplitude | 4 | SSD participant age | — |  |  |  |
|  | 4 | Sex (% of male SSD participants) | — |  |  |  |
|  | 1 | Duration of illness (M) | — |  |  |  |
|  | 1 | PANSS total score | — |  |  |  |
|  | 1 | PANSS positive scale score | — |  |  |  |
|  | 1 | PANSS negative scale score | — |  |  |  |
|  | 1 | PANSS general psychopathology scale score | — |  |  |  |
|  | 2 | Antipsychotic dose (chlorpromazine equivalent [mg/day]) | — |  |  |  |
|  | 4 | NOS | — |  |  |  |
|  |  |  |  |  |  |  |
| b-wave amplitude | 4 | SSD participant age | — |  |  |  |
|  | 4 | Sex (% of male SSD participants) | — |  |  |  |
|  | 1 | Duration of illness (M) | — |  |  |  |
|  | 1 | PANSS total score | — |  |  |  |
|  | 0 | PANSS positive scale score | — |  |  |  |
|  | 0 | PANSS negative scale score | — |  |  |  |
|  | 0 | PANSS general psychopathology scale score | — |  |  |  |
|  | 1 | Antipsychotic dose (chlorpromazine equivalent [mg/day]) | — |  |  |  |
|  | 4 | NOS | — |  |  |  |
|  |  |  |  |  |  |  |
| Latency |  |  |  |  |  |  |
| Photopic fERG |  |  |  |  |  |  |
| a-wave latency | 6 | SSD participant age | 0.0118 | [−0.0257; 0.0493] | 0.0191 | 0.5386 |
|  | 6 | Sex (% of male SSD participants) | 0.0028 | [−0.0144; 0.0200] | 0.0088 | 0.7482 |
|  | 3 | Duration of illness (M) | — |  |  |  |
|  | 3 | PANSS total score | — |  |  |  |
|  | 3 | PANSS positive scale score | — |  |  |  |
|  | 3 | PANSS negative scale score | — |  |  |  |
|  | 3 | PANSS general psychopathology scale score | — |  |  |  |
|  | 4 | Antipsychotic dose (chlorpromazine equivalent [mg/day]) | — |  |  |  |
|  | 6 | NOS | 0.3244 | [0.0154; 0.6333] | 0.1576 | 0.0396 |
|  |  |  |  |  |  |  |
| b-wave latency | 4 | SSD participant age | — |  |  |  |
|  | 4 | Sex (% of male SSD participants) | — |  |  |  |
|  | 2 | Duration of illness (M) | — |  |  |  |
|  | 2 | PANSS total score | — |  |  |  |
|  | 2 | PANSS positive scale score | — |  |  |  |
|  | 2 | PANSS negative scale score | — |  |  |  |
|  | 2 | PANSS general psychopathology scale score | — |  |  |  |
|  | 3 | Antipsychotic dose (chlorpromazine equivalent [mg/day]) | — |  |  |  |
|  | 4 | NOS | — |  |  |  |
|  |  |  |  |  |  |  |
| Scotopic fERG |  |  |  |  |  |  |
| a-wave latency | 4 | SSD participant age | — |  |  |  |
|  | 4 | Sex (% of male SSD participants) | — |  |  |  |
|  | 1 | Duration of illness (M) | — |  |  |  |
|  | 1 | PANSS total score | — |  |  |  |
|  | 1 | PANSS positive scale score | — |  |  |  |
|  | 1 | PANSS negative scale score | — |  |  |  |
|  | 1 | PANSS general psychopathology scale score | — |  |  |  |
|  | 2 | Antipsychotic dose (chlorpromazine equivalent [mg/day]) | — |  |  |  |
|  | 4 | NOS | — |  |  |  |
|  |  |  |  |  |  |  |
| b-wave latency | 3 | SSD participant age | — |  |  |  |
|  | 3 | Sex (% of male SSD participants) | — |  |  |  |
|  | 1 | Duration of illness (M) | — |  |  |  |
|  | 1 | PANSS total score | — |  |  |  |
|  | 0 | PANSS positive scale score | — |  |  |  |
|  | 0 | PANSS negative scale score | — |  |  |  |
|  | 0 | PANSS general psychopathology scale score | — |  |  |  |
|  | 1 | Antipsychotic dose (chlorpromazine equivalent [mg/day]) | — |  |  |  |
|  | 3 | NOS | — |  |  |  |

Abbreviations: CI: confidence interval; fERG, flash electroretinogram; NOS, Newcastle–Ottawa Scale; PANSS: Positive and Negative Symptom Scale; SD-OCT, spectral domain–optical coherence tomography; SS-OCT, swept source–optical coherence tomography; TD-OCT: time domain–optical coherence tomography.

# **PRISMA 2020 checklist.**

| **Section and Topic** | **Item No.** | **Checklist item** | **Location where item is reported** |
| --- | --- | --- | --- |
| **TITLE** | | |  |
| Title | 1 | Identify the report as a systematic review. | P.1 |
| **ABSTRACT** | | |  |
| Abstract | 2 | See the PRISMA 2020 for Abstracts checklist. | P.4-5 |
| **INTRODUCTION** | | |  |
| Rationale | 3 | Describe the rationale for the review in the context of existing knowledge. | P.6-9 |
| Objectives | 4 | Provide an explicit statement of the objective(s) or question(s) the review addresses. | P.6-9 |
| **METHODS** | | |  |
| Eligibility criteria | 5 | Specify the inclusion and exclusion criteria for the review and how studies were grouped for the syntheses. | P.10-11 |
| Information sources | 6 | Specify all databases, registers, websites, organizations, reference lists, and other sources searched or consulted to identify studies. Specify the date when each source was last searched or consulted. | P.9-11 |
| Search strategy | 7 | Present the full search strategies for all databases, registers, and websites, including any filters and limits used. | P.9-11 |
| Selection process | 8 | Specify the methods used to decide whether a study met the inclusion criteria of the review, including how many reviewers screened each record and each report retrieved, whether they worked independently, and, if applicable, details of the automation tools used in the process. | P.9-11. Fig.1 |
| Data collection process | 9 | Specify the methods used to collect data from reports, including how many reviewers collected data from each report, whether they worked independently, any processes for obtaining or confirming data from study investigators, and, if applicable, details of the automation tools used in the process. | P.9-10. Fig.1 |
| Data items | 10a | List and define all outcomes for which data were sought. Specify whether all results that were compatible with each outcome domain in each study were sought (e.g., for all measures, time points, analyses) and, if not, the methods used to decide which results to collect. | P.11-12. Supplementary information P.6 |
|  | 10b | List and define all other variables for which data were sought (e.g., participant and intervention characteristics, funding sources). Describe any assumptions made about any missing or unclear information. | P.10-12. Supplementary  information P.6 |
| Study risk of bias assessment | 11 | Specify the methods used to assess risk of bias in the included studies, including details of the tool(s) used, how many reviewers assessed each study and whether they worked independently, and, if applicable, details of the automation tools used in the process. | P.11 |
| Effect measures | 12 | Specify for each outcome the effect measure(s) (e.g., risk ratio, mean difference) used in the synthesis or presentation of results. | P.11-12. Supplementary  information P.6 |
| Synthesis methods | 13a | Describe the processes used to decide which studies were eligible for each synthesis (e.g., tabulating the study intervention characteristics and comparing against the planned groups for each synthesis [item #5]). | P.11-12. Supplementary  information P.6 |
|  | 13b | Describe any methods required to prepare the data for presentation or synthesis, such as handling of missing summary statistics or data conversions. | P.10-12. |
|  | 13c | Describe any methods used to tabulate or visually display the results of individual studies and syntheses. | P.11-12. Supplementary  information P.6 |
|  | 13d | Describe any methods used to synthesize the results, and provide a rationale for the choice(s). If meta-analysis was performed, describe the model(s), method(s) to identify the presence and extent of statistical heterogeneity, and software package(s) used. | P.11-12. Supplementary  information P.6 |
|  | 13e | Describe any methods used to explore possible causes of heterogeneity among study results (e.g., subgroup analysis, meta-regression). | P.11-12. Supplementary  information P.6 |
|  | 13f | Describe any sensitivity analyses conducted to assess the robustness of the synthesized results. | P.11-12. Supplementary  information P.6. |
| Reporting bias assessment | 14 | Describe any methods used to assess risk of bias due to missing results in a synthesis (arising from reporting biases). | P.11. Supplementary  information P.6. Table S1-3. |
| Certainty assessment | 15 | Describe any methods used to assess certainty (or confidence) in the body of evidence for an outcome. | P.11. Supplementary  information P.6. Table S6. |
| **RESULTS** | | |  |
| Study selection | 16a | Describe the results of the search and selection process, from the number of records identified in the search to the number of studies included in the review, ideally using a flow diagram. | P.13. Fig.1 |
|  | 16b | Cite studies that might appear to meet the inclusion criteria but that were excluded, and explain why they were excluded. | P.13. Fig.1 |
| Study characteristics | 17 | Cite each included study and present its characteristics. | Table S1-3. |
| Risk of bias in studies | 18 | Present assessments of risk of bias for each included study. | Table S1-3. |
| Results of individual studies | 19 | For all outcomes, present, for each study: (a) summary statistics for each group (where appropriate) and (b) an effect estimate and its precision (e.g., confidence/credible interval), ideally using structured tables or plots. | Fig.2,4, Fig.S1,16,24,54,61 |
| Results of syntheses | 20a | For each synthesis, briefly summarize the characteristics and risk of bias among contributing studies. | P.13- 21. Table S1-3, S6. |
|  | 20b | Present the results of all statistical syntheses conducted. If meta-analysis was done, present for each the summary estimate and its precision (e.g., confidence/credible interval) and measures of statistical heterogeneity. If comparing groups, describe the direction of the effect. | P.13- 21. Fig.2,4. Fig.S1,16,24,54,61. |
|  | 20c | Present results of all investigations of possible causes of heterogeneity among study results. | P.13- 21. Table 1-2, Table S12. |
|  | 20d | Present results of all sensitivity analyses conducted to assess the robustness of the synthesized results. | P.13- 21. Fig.S2-11, 17-20, 25-26, 30-37, 39-46, 48-49, 51, 56-57, 59-60, 62. Table S4,7-10. |
| Reporting biases | 21 | Present assessments of risk of bias due to missing results (arising from reporting biases) for each synthesis assessed. | P.13- 21. Fig.S12-13, 21, 27, 38, 47, 52, 55, 58, 63. Table S5, 11. |
| Certainty of evidence | 22 | Present assessments of certainty (or confidence) in the body of evidence for each outcome assessed. | P.13- 21. Table.S6. |
| **DISCUSSION** | | |  |
| Discussion | 23a | Provide a general interpretation of the results in the context of other evidence. | P.22-31. |
|  | 23b | Discuss any limitations of the evidence included in the review. | P.22-31. |
|  | 23c | Discuss any limitations of the review processes used. | P.22-31. |
|  | 23d | Discuss implications of the results for practice, policy, and future research. | P.22-31. |
| **OTHER INFORMATION** | | |  |
| Registration and protocol | 24a | Provide registration information for the review, including register name and registration number, or state that the review was not registered. | P.10 |
|  | 24b | Indicate where the review protocol can be accessed, or state that a protocol was not prepared. | P.10 |
|  | 24c | Describe and explain any amendments to information provided at registration or in the protocol. | P.10 |
| Support | 25 | Describe sources of financial or nonfinancial support for the review and the role of the funders or sponsors in the review. | P.31-32 |
| Competing interests | 26 | Declare any competing interests of the review authors. | P.31-32 |
| Availability of data, code, and other materials | 27 | Report which of the following are publicly available and where they can be found: template data collection forms, data extracted from included studies, data used for all analyses, analytic code, and any other materials used in the review. | P.31 |

# **Reference**

1.	Xu Q, Li Y, Cheng Y, Qu Y. Assessment of the effect of age on macular layer thickness in a healthy Chinese cohort using spectral-domain optical coherence tomography. *BMC Ophthalmol* 2018; **18**(1)**:** 169.

2.	Komatsu H, Onoguchi G, Jerotic S, Kanahara N, Kakuto Y, Ono T *et al.* Retinal layers and associated clinical factors in schizophrenia spectrum disorders: a systematic review and meta-analysis. *Mol Psychiatry* 2022; **27**(9)**:** 3592-3616.

3.	Higgins JP, Thompson SG, Deeks JJ, Altman DG. Measuring inconsistency in meta-analyses. *Bmj* 2003; **327**(7414)**:** 557-560.

4.	Cochran WG. The Combination of Estimates from Different Experiments. *Biometrics* 1954; **10**(1)**:** 101-129.

5.	DerSimonian R, Kacker R. Random-effects model for meta-analysis of clinical trials: an update. *Contemp Clin Trials* 2007; **28**(2)**:** 105-114.

6.	Viechtbauer W, Cheung MW. Outlier and influence diagnostics for meta-analysis. *Res Synth Methods* 2010; **1**(2)**:** 112-125.

7.	Olkin I, Dahabreh IJ, Trikalinos TA. GOSH - a graphical display of study heterogeneity. *Res Synth Methods* 2012; **3**(3)**:** 214-223.

8.	Egger M, Davey Smith G, Schneider M, Minder C. Bias in meta-analysis detected by a simple, graphical test. *Bmj* 1997; **315**(7109)**:** 629-634.

9.	Duval S, Tweedie R. Trim and fill: A simple funnel-plot-based method of testing and adjusting for publication bias in meta-analysis. *Biometrics* 2000; **56**(2)**:** 455-463.

10.	Kay SR, Fiszbein A, Opler LA. The positive and negative syndrome scale (PANSS) for schizophrenia. *Schizophr Bull* 1987; **13**(2)**:** 261-276.

11.	Balduzzi S, Rücker G, Schwarzer G. How to perform a meta-analysis with R: a practical tutorial. *Evid Based Ment Health* 2019; **22**(4)**:** 153-160.

12.	Viechtbauer W. Conducting Meta-Analyses in R with the metafor Package. *Journal of Statistical Software* 2010; **36**(3)**:** 1 - 48.

13.	Harrer M, Cuijpers P, Furukawa T, Ebert D. Doing Meta-Analysis with R: A Hands-On Guide (1st ed.). 2021.

14.	Carriello MA, Costa DFB, Alvim PHP, Pestana MC, Bicudo DDS, Gomes EMP *et al.* Retinal layers and symptoms and inflammation in schizophrenia. *Eur Arch Psychiatry Clin Neurosci* 2023.

15.	Kurtulmus A, Sahbaz C, Elbay A, Guler EM, Sonmez Avaroglu G, Kocyigit A *et al.* Clinical and biological correlates of optical coherence tomography findings in schizophrenia. *Eur Arch Psychiatry Clin Neurosci* 2023.

16.	Shoham N, Lewis G, Hayes JF, Silverstein SM, Cooper C. Association between visual impairment and psychosis: A longitudinal study and nested case-control study of adults. *Schizophr Res* 2023; **254:** 81-89.

17.	Wagner SK, Cortina-Borja M, Silverstein SM, Zhou Y, Romero-Bascones D, Struyven RR *et al.* Association Between Retinal Features From Multimodal Imaging and Schizophrenia. *JAMA Psychiatry* 2023.

18.	Boudriot E, Schworm B, Slapakova L, Hanken K, Jäger I, Stephan M *et al.* Optical coherence tomography reveals retinal thinning in schizophrenia spectrum disorders. *Eur Arch Psychiatry Clin Neurosci* 2022.

19.	Bozali E, Yalinbas D. Analysis of the Thickness of the Outer Retinal Layer Using Optical Coherence Tomography - A Predictor of Visual Acuity in Schizophrenia. *Klin Monbl Augenheilkd* 2022; **239**(10)**:** 1232-1238.

20.	Friedel EBN, Hahn HT, Maier S, Küchlin S, Reich M, Runge K *et al.* Structural and functional retinal alterations in patients with paranoid schizophrenia. *Transl Psychiatry* 2022; **12**(1)**:** 402.

21.	Kango A, Grover S, Gupta V, Sahoo S, Nehra R. A comparative study of retinal layer changes among patients with schizophrenia and healthy controls. *Acta Neuropsychiatr* 2022**:** 1-12.

22.	Kaya H, Ayık B, Tasdelen R, Sevimli N, Ertekin E. Comparing retinal changes measured by optical coherence tomography in patients with schizophrenia and their siblings with healthy controls: Are retinal findings potential endophenotype candidates? *Asian J Psychiatr* 2022; **72:** 103089.

23.	Khalil DH, Aziz K, Khalil M, Khowyled A. Optical coherence tomography in Egyptian schizophrenics and its correlation to disease parameters. *Delta Journal of Ophthalmology* 2022; **23**(3)**:** 198-205.

24.	Kokacya MH, Cakmak AI. Optical Coherence Tomography Angiography in Schizophrenia. *Alpha Psychiatry* 2022; **23**(5)**:** 253-261.

25.	Kurt A, Zor KR, Kucuk E, Yildirim G, Ersan EE. An Optical Coherence Tomography Study that Supports the Neurovascular Basis of Schizophrenia Disease. *Alpha Psychiatry* 2022; **23**(1)**:** 12-17.

26.	Asanad S, O'Neill H, Addis H, Chen S, Wang J, Goldwaser E *et al.* Neuroretinal Biomarkers for Schizophrenia Spectrum Disorders. *Transl Vis Sci Technol* 2021; **10**(4)**:** 29.

27.	Alizadeh M, Delborde Y, Ahmadpanah M, Seifrabiee MA, Jahangard L, Bazzazi N *et al.* Non-linear associations between retinal nerve fibre layer (RNFL) and positive and negative symptoms among men with acute and chronic schizophrenia spectrum disorder. *J Psychiatr Res* 2021; **141:** 81-91.

28.	Gandu S, Bannai D, Adhan I, Kasetty M, Katz R, Zang R *et al.* Inter-device reliability of swept source and spectral domain optical coherence tomography and retinal layer differences in schizophrenia. *Biomarkers in Neuropsychiatry* 2021; **5**.

29.	Koman-Wierdak E, Róg J, Brzozowska A, Toro MD, Bonfiglio V, Załuska-Ogryzek K *et al.* Analysis of the Peripapillary and Macular Regions Using OCT Angiography in Patients with Schizophrenia and Bipolar Disorder. *J Clin Med* 2021; **10**(18).

30.	Jerotic S, Lalovic N, Pejovic S, Mihaljevic M, Pavlovic Z, Britvic D *et al.* Sex differences in macular thickness of the retina in patients with psychosis spectrum disorders. *Prog Neuropsychopharmacol Biol Psychiatry* 2021; **110:** 110280.

31.	Liu Y, Chen J, Huang L, Yan S, Bian Q, Yang F. Relationships Among Retinal Nerve Fiber Layer Thickness, Vascular Endothelial Growth Factor, and Cognitive Impairment in Patients with Schizophrenia. *Neuropsychiatr Dis Treat* 2021; **17:** 3597-3606.

32.	Murav'eva SV, Kozub KE, Pronin SV. Optical and electrophysiological techniques for functional assessment of vision system neuronal networks. *Journal of Optical Technology* 2021; **88**(12)**:** 710-715.

33.	Sarkar S, Rajalakshmi AR, Avudaiappan S, Eswaran S. Exploring the role of macular thickness as a potential early biomarker of neurodegeneration in acute schizophrenia. *Int Ophthalmol* 2021; **41**(8)**:** 2737-2746.

34.	Silverstein SM, Lai A, Green KM, Crosta C, Fradkin SI, Ramchandran RS. Retinal Microvasculature in Schizophrenia. *Eye Brain* 2021; **13:** 205-217.

35.	Zhuo C, Xiao B, Chen C, Jiang D, Li G, Ma X *et al.* Abberant inverted U-shaped brain pattern and trait-related retinal impairment in schizophrenia patients with combined auditory and visual hallucinations: a pilot study. *Brain Imaging Behav* 2021; **15**(2)**:** 738-747.

36.	Zhuo C, Xiao B, Ji F, Lin X, Jiang D, Tian H *et al.* Patients with first-episode untreated schizophrenia who experience concomitant visual disturbances and auditory hallucinations exhibit co-impairment of the brain and retinas-a pilot study. *Brain Imaging Behav* 2021; **15**(3)**:** 1533-1541.

37.	Altun IK, Turedi N, Aras N, Atagun MI. Psychopharmacological Signatures in the Retina in Schizophrenia and Bipolar Disorder: An Optic Coherence Tomography Study. *Psychiatr Danub* 2020; **32**(3-4)**:** 351-358.

38.	Budakoglu O, Ozdemir K, Safak Y, Sen E, Taskale B. Retinal nerve fibre layer and peripapillary vascular density by optical coherence tomography angiography in schizophrenia. *Clin Exp Optom* 2021; **104**(7)**:** 788-794.

39.	Hosák L, Zeman T, Studnička J, Stepanov A, Ustohal L, Michalec M *et al.* Retinal arteriolar and venular diameters are widened in patients with schizophrenia. *Psychiatry Clin Neurosci* 2020; **74**(11)**:** 619-621.

40.	Huang JJ, Song XQ, Xu Y, Wang LN, Li YC, Tian HJ *et al.* Reliability and Diagnostic Validity of A Novel Visual Disturbance Subjective Experience Scale in Chinese Patients with Schizophrenia. *Psychiatry and Clinical Psychopharmacology* 2020; **30**(3)**:** 109-114.

41.	Jerotic S, Ristic I, Pejovic S, Mihaljevic M, Pavlovic Z, Britvic D *et al.* Retinal structural abnormalities in young adults with psychosis spectrum disorders. *Prog Neuropsychopharmacol Biol Psychiatry* 2020; **98:** 109825.

42.	Kozub KE, Shelepin IE, Chomskii AN, Sharybin EA, Ivanova EA. A structural and functional study of the retina in patients with schizophrenia. *Oftalmologicheskii Zhurnal* 2020; (4)**:** 38-44.

43.	Kurtulmus A, Elbay A, Parlakkaya FB, Kilicarslan T, Ozdemir MH, Kirpinar I. An investigation of retinal layer thicknesses in unaffected first-degree relatives of schizophrenia patients. *Schizophr Res* 2020; **218:** 255-261.

44.	Lai A, Crosta C, Loftin M, Silverstein SM. Retinal structural alterations in chronic versus first episode schizophrenia spectrum disorders. *Biomarkers in Neuropsychiatry* 2020; **2**.

45.	Liu Y, Huang L, Tong Y, Chen J, Gao D, Yang F. Association of retinal nerve fiber abnormalities with serum CNTF and cognitive functions in schizophrenia patients. *PeerJ* 2020; **8:** e9279.

46.	Miller M, Zemon V, Nolan-Kenney R, Balcer LJ, Goff DC, Worthington M *et al.* Optical coherence tomography of the retina in schizophrenia: Inter-device agreement and relations with perceptual function. *Schizophr Res* 2020; **219:** 13-18.

47.	Orum MH, Bulut M, Karadag AS, Dumlupinar E, Kalenderoglu A. Comparison of OCT findings of schizophrenia patients using FGA, Clozapine, and SGA other than Clozapine. *Archives of Clinical Psychiatry* 2020; **47**(6)**:** 165-175.

48.	Schönfeldt-Lecuona C, Kregel T, Schmidt A, Kassubek J, Dreyhaupt J, Freudenmann RW *et al.* Retinal single-layer analysis with optical coherence tomography (OCT) in schizophrenia spectrum disorder. *Schizophr Res* 2020; **219:** 5-12.

49.	Zhuo CJ, Ji F, Xiao B, Lin XD, Chen C, Jiang DG *et al.* Antipsychotic agent-induced deterioration of the visual system in first-episode untreated patients with schizophrenia maybe self-limited: Findings from a secondary small sample follow-up study based on a pilot follow-up study. *Psychiatry Research* 2020; **286**.

50.	Zhuo CJ, Xiao B, Chen C, Jiang DG, Li GY, Ma XY *et al.* Antipsychotic agents deteriorate brain and retinal function in schizophrenia patients with combined auditory and visual hallucinations: A pilot study and secondary follow-up study. *Brain and Behavior* 2020; **10**(6).

51.	Topcu-Yilmaz P, Aydin M, Ilhan BC. Evaluation of retinal nerve fiber layer, macular, and choroidal thickness in schizophrenia: spectral optic coherence tomography findings. *Psychiatry and Clinical Psychopharmacology* 2019; **29**(1)**:** 28-33.

52.	Delıbaş DH, Karti Ö, Erdoğan E, Şahın T, Bılgıç Ö, Erol A. Decreases in retinal nerve fiber layer and ganglion cell-inner plexiform layer thickness in schizophrenia, relation to insight: A controlled study. *Anadolu Psikiyatri Dergisi* 2018; **19**(3)**:** 264-273.

53.	Samani NN, Proudlock FA, Siram V, Suraweera C, Hutchinson C, Nelson CP *et al.* Retinal Layer Abnormalities as Biomarkers of Schizophrenia. *Schizophr Bull* 2018; **44**(4)**:** 876-885.

54.	Silverstein SM, Paterno D, Cherneski L, Green S. Optical coherence tomography indices of structural retinal pathology in schizophrenia. *Psychol Med* 2018; **48**(12)**:** 2023-2033.

55.	Celik M, Kalenderoglu A, Sevgi Karadag A, Bekir Egilmez O, Han-Almis B, Şimşek A. Decreases in ganglion cell layer and inner plexiform layer volumes correlate better with disease severity in schizophrenia patients than retinal nerve fiber layer thickness: Findings from spectral optic coherence tomography. *Eur Psychiatry* 2016; **32:** 9-15.

56.	Yilmaz U, Kucuk E, Ulgen A, Ozkose A, Demircan S, Ulusoy DM *et al.* Retinal nerve fiber layer and macular thickness measurement in patients with schizophrenia. *European Journal of Ophthalmology* 2016; **26**(4)**:** 375-378.

57.	Ascaso FJ, Rodriguez-Jimenez R, Cabezón L, López-Antón R, Santabárbara J, De la Cámara C *et al.* Retinal nerve fiber layer and macular thickness in patients with schizophrenia: Influence of recent illness episodes. *Psychiatry Res* 2015; **229**(1-2)**:** 230-236.

58.	Mota M, Pêgo P, Klut C, Coutinho I, Santos C, Pires G *et al.* Evaluation of Structural Changes in the Retina of Patients with Schizophrenia. *Ophthalmology Research* 2015; **4**(2)**:** 45-52.

59.	Lee WW, Tajunisah I, Sharmilla K, Peyman M, Subrayan V. Retinal nerve fiber layer structure abnormalities in schizophrenia and its relationship to disease state: evidence from optical coherence tomography. *Invest Ophthalmol Vis Sci* 2013; **54**(12)**:** 7785-7792.

60.	Chu EMY, Kolappan M, Barnes TRE, Joyce EM, Ron MA. A window into the brain: An in vivo study of the retina in schizophrenia using optical coherence tomography. *Psychiatry Research-Neuroimaging* 2012; **203**(1)**:** 89-94.

61.	Ascaso FJ, Cabezon L, Quintanilla MA, Galve LG, Lopez-Anton R, Cristobal JA *et al.* Retinal nerve fiber layer thickness measured by optical coherence tomography in patients with schizophrenia: A short report. *European Journal of Psychiatry* 2010; **24**(4)**:** 227-235.

62.	Bannai D, Adhan I, Katz R, Kim LA, Keshavan M, Miller JB *et al.* Quantifying Retinal Microvascular Morphology in Schizophrenia Using Swept-Source Optical Coherence Tomography Angiography. *Schizophr Bull* 2022; **48**(1)**:** 80-89.

63.	Bernardin F, Schwitzer T, Schwan R, Angioi-Duprez K, Ligier F, Bourion-Bedes S *et al.* Altered central vision and amacrine cells dysfunction as marker of hypodopaminergic activity in treated patients with schizophrenia. *Schizophr Res* 2022; **239:** 134-141.

64.	Bernardin F, Schwitzer T, Angioi-Duprez K, Giersch A, Ligier F, Bourion-Bedes S *et al.* Retinal ganglion cell dysfunction is correlated with disturbed visual cognition in schizophrenia patients with visual hallucinations. *Psychiatry Res* 2021; **298:** 113780.

65.	Bernardin F, Schwitzer T, Angioi-Duprez K, Giersch A, Ligier F, Bourion-Bedes S *et al.* Retinal dysfunctions in a patient with a clinical high risk for psychosis and severe visual disturbances: A single case report. *Early Interv Psychiatry* 2021; **15**(6)**:** 1784-1788.

66.	Moghimi P, Torres Jimenez N, McLoon LK, Netoff TI, Lee MS, MacDonald A, 3rd *et al.* Electoretinographic evidence of retinal ganglion cell-dependent function in schizophrenia. *Schizophr Res* 2020; **219:** 34-46.

67.	Hébert M, Mérette C, Gagné AM, Paccalet T, Moreau I, Lavoie J *et al.* The Electroretinogram May Differentiate Schizophrenia From Bipolar Disorder. *Biol Psychiatry* 2020; **87**(3)**:** 263-270.

68.	Fradkin SI, Erickson MA, Demmin DL, Silverstein SM. Absence of Excess Intra-Individual Variability in Retinal Function in People With Schizophrenia. *Front Psychiatry* 2020; **11:** 543963.

69.	Demmin DL, Netser R, Roché MW, Thompson JL, Silverstein SM. People with current major depression resemble healthy controls on flash Electroretinogram indices associated with impairment in people with stabilized schizophrenia. *Schizophr Res* 2020; **219:** 69-76.

70.	Demmin DL, Mote J, Beaudette DM, Thompson JL, Silverstein SM. Retinal functioning and reward processing in schizophrenia. *Schizophr Res* 2020; **219:** 25-33.

71.	Bernardin F, Schwitzer T, Angioi-Duprez K, Giersch A, Jansen C, Schwan R *et al.* Retinal ganglion cells dysfunctions in schizophrenia patients with or without visual hallucinations. *Schizophr Res* 2020; **219:** 47-55.

72.	Demmin DL, Davis Q, Roché M, Silverstein SM. Electroretinographic anomalies in schizophrenia. *J Abnorm Psychol* 2018; **127**(4)**:** 417-428.

73.	Hébert M, Mérette C, Paccalet T, Émond C, Gagné AM, Sasseville A *et al.* Light evoked potentials measured by electroretinogram may tap into the neurodevelopmental roots of schizophrenia. *Schizophr Res* 2015; **162**(1-3)**:** 294-295.

74.	Balogh Z, Benedek G, Kéri S. Retinal dysfunctions in schizophrenia. *Prog Neuropsychopharmacol Biol Psychiatry* 2008; **32**(1)**:** 297-300.

75.	Warner R, Laugharne J, Peet M, Brown L, Rogers N. Retinal function as a marker for cell membrane omega-3 fatty acid depletion in schizophrenia: a pilot study. *Biol Psychiatry* 1999; **45**(9)**:** 1138-1142.

76.	Gerbaldo H, Thaker G, Tittel PG, Layne-Gedge J, Moran M, Demisch L. Abnormal electroretinography in schizophrenic patients with a history of sun gazing. *Neuropsychobiology* 1992; **25**(2)**:** 99-101.

77.	Marmor MF, Hock P, Schechter G, Pfefferbaum A, Berger PA, Maurice R. Oscillatory potentials as a marker for dopaminergic disease. *Doc Ophthalmol* 1988; **69**(3)**:** 255-261.

78.	Raese JD, King RJ, Barnes D. Retinal oscillatory potentials in schizophrenia: Implications for the assessment of dopamine transmission in man. *Psychopharmacology Bulletin* 1982; **18**(3)**:** 72-78.
